# Caveolin-1 Modulates Mechanotransduction Responses to Substrate Stiffness through Actin-Dependent Control of YAP

**DOI:** 10.1016/j.celrep.2018.10.024

**Published:** 2018-11-06

**Authors:** Roberto Moreno-Vicente, Dácil María Pavón, Inés Martín-Padura, Mauro Català-Montoro, Alberto Díez-Sánchez, Antonio Quílez-Álvarez, Juan Antonio López, Miguel Sánchez-Álvarez, Jesús Vázquez, Raffaele Strippoli, Miguel A. del Pozo

**Affiliations:** 1 Mechanoadaptation & Caveolae Biology Lab, Cell and Developmental Biology Area, Centro Nacional de Investigaciones Cardiovasculares (CNIC), Madrid 28029, Spain; 2 Cardiovascular Proteomics Unit, CNIC, Madrid 28029, Spain; 3 Section of Molecular Genetics, Department of Cellular Biotechnologies and Hematology, Istituto Pasteur-Fondazione Cenci Bolognetti, Sapienza University of Rome, Rome 00161, Italy

## Abstract

The transcriptional regulator YAP orchestrates many cellular functions, including tissue homeostasis, organ growth control, and tumorigenesis. Mechanical stimuli are a key input to YAP activity, but the mechanisms controlling this regulation remain largely uncharacterized. We show that CAV1 positively modulates the YAP mechanoresponse to substrate stiffness through actin-cytoskeleton-dependent and Hippo-kinase-independent mechanisms. RHO activity is necessary, but not sufficient, for CAV1-dependent mechanoregulation of YAP activity. Systematic quantitative interactomic studies and image-based small interfering RNA (siRNA) screens provide evidence that this actin-dependent regulation is determined by YAP interaction with the 14-3-3 protein YWHAH. Constitutive YAP activation rescued phenotypes associated with CAV1 loss, including defective extracellular matrix (ECM) remodeling. CAV1-mediated control of YAP activity was validated *in vivo* in a model of pancreatitis-driven acinar-to-ductal metaplasia. We propose that this CAV1-YAP mechanotransduction system controls a significant share of cell programs linked to these two pivotal regulators, with potentially broad physiological and pathological implications.

## Highlights

• CAV1 modulates YAP activity in response to changes in ECM stiffness
• CAV1 regulates YAP through the control of actin dynamics and YAP phosphorylation
• YWHAH-YAP interaction blunts YAP activity in CAV1-deficient cells
• CAV1-YAP mechanotransduction drives ECM remodeling and pancreatic ADM

## Graphical Abstract

**Figure:**
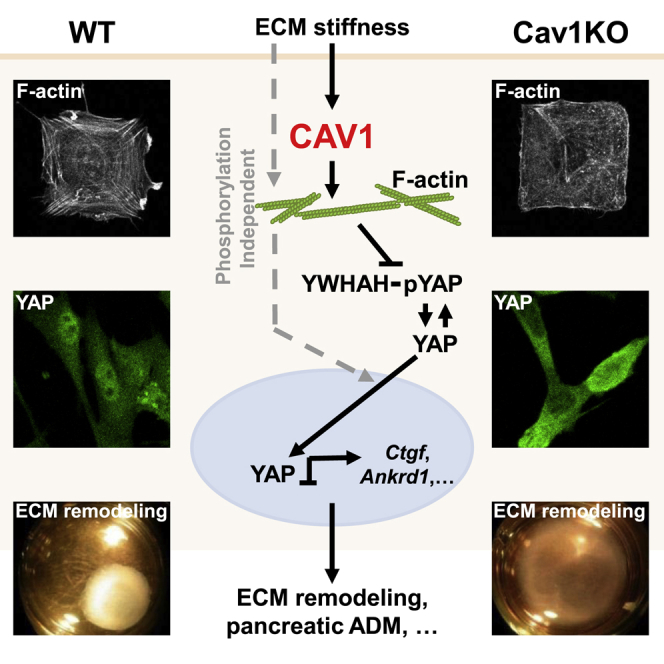

## Teaser

Moreno-Vicente et al. report that CAV1, a key component of PM mechanosensing caveolae, mediates adaptation to ECM rigidity by modulating YAP activity through the control of actin dynamics and phosphorylation-dependent interaction of YAP with the 14-3-3-domain protein YWHAH. Cav1-dependent YAP regulation drives two pathophysiological processes: ECM remodeling and pancreatic ADM.

## Introduction

The integral membrane protein Caveolin-1 (CAV1) engages in crosstalk with the actin cytoskeleton and connects directly to actin cables through the protein FLNA (Muriel et al., 2011, Stahlhut and van Deurs, 2000). CAV1 controls focal adhesion stability, actin organization, and actomyosin contraction through RHO GTPases (Echarri et al., 2007, Goetz et al., 2011, Grande-García et al., 2007) and contributes to mechanosensing and adaptation in response to various mechanical stimuli, such as membrane stretching, shear stress, hypoosmotic shock, and cell detachment (Boyd et al., 2003, Muriel et al., 2011, Sinha et al., 2011). However, current understanding remains limited regarding the mechanisms by which these phenomena are integrated with overall cell function.

The transcriptional cofactor yes-associated protein (YAP) operates downstream of the canonical Hippo pathway (Piccolo et al., 2014), a highly conserved pathway regulating organ growth control, tissue homeostasis, and tumorigenesis (Yu et al., 2015). YAP regulates the transcription of specific gene sets mainly through its interaction with TEA domain (TEAD) transcription factors (Zhao et al., 2008). A cascade of kinases, including LATS1 and LATS2, lead to YAP phosphorylation and curb its nucleocytoplasmic shuttling, mediating its cytosolic retention through interaction with 14-3-3 proteins, thus downregulating YAP transcriptional output (Dong et al., 2007, Hao et al., 2008, Zhao et al., 2007). This regulatory network is controlled by upstream cues related to tissue architecture and cellular context, such as cell-cell adhesion, cell density, and cell polarity (Piccolo et al., 2014). YAP is also controlled by mechanical signals, such as extracellular matrix (ECM) stiffness, shear stress, and stretching (Codelia et al., 2014, Dupont et al., 2011, Zhong et al., 2013). Stiff environments favor YAP nuclear localization (i.e., activation), whereas attachment to soft substrates increases cytoplasmic retention. This mechanical control, which determines cell proliferation and differentiation (Dupont et al., 2011), depends on RHO GTPase function and actomyosin-driven contractility but is largely independent of kinase regulation, because (1) depletion of LATS1/2 kinases does not alter the mechanical responsiveness of YAP and (2) non-phosphorylatable mutants are nonetheless sensitive to substrate stiffness (Dupont et al., 2011, Elosegui-Artola et al., 2017). The adaptation of nuclear pore units to mechanical tension also contributes to the regulation of YAP nuclear entry (Elosegui-Artola et al., 2017). However, understanding is limited about the exact molecular mechanisms by which ECM stiffness controls YAP activity. Here, we identify CAV1 as an upstream positive regulator of YAP that affects the response to changes in ECM stiffness through a mechanism dependent on F-actin dynamics.

The mechanical regulation of YAP underpins pathophysiological processes such as cardiovascular disease, inflammation and tissue regeneration, and cancer (Panciera et al., 2017). YAP activation by ECM stiffness promotes cancer-associated fibroblast activation and subsequent peritumoral ECM remodeling and stiffening, establishing a positive-feedback loop that favors cancer progression (Calvo et al., 2013). Here, we show that overexpression of constitutively active YAP mutants rescues the blunted contractility and ECM remodeling previously reported for *Cav1* genetic deficiency (Goetz et al., 2011). The positive impact of YAP activity on tumor initiation and progression is further showcased by its critical contribution to pancreatitis-induced acinar-to-ductal metaplasia (ADM), which favors pancreatic ductal carcinoma (PDAC) initiation (Gruber et al., 2016). We further demonstrate CAV1-dependent positive regulation of YAP *in vivo*, showing that Cav1-knockout (Cav1KO) pancreatic parenchyma fails to upregulate YAP in response to induced pancreatitis and exhibits blunting of changes associated with YAP activation, such as ADM.

Our results provide important insight into the mechanisms regulating YAP function. We identify CAV1 as an upstream regulator of YAP, controlling its transcriptional activity through the control of actin cytoskeleton dynamics. Conversely, YAP underpins an important share of CAV1-dependent phenotypes. We propose this CAV1-YAP regulation has important implications in the progression of some pathologies, such as cancer, and will allow us to better understand the principles governing processes driven by substrate stiffness in health and disease.

## Results

### CAV1 Positively Regulates YAP Activity by Controlling YAP Nucleocytoplasmic Shuttling

ECM stiffness mediates CAV1 internalization (Du et al., 2011). We confirmed that CAV1 was internalized in cells grown on soft substrates (Figure S1A) and trafficked to a RAB11-positive recycling endosome (Figure S1B). Thus, cell detachment from integrin-ECM-mediated adhesions and cell growth on soft substrates both trigger the same translocation of CAV1 from the plasma membrane toward a recycling endosome (del Pozo et al., 2005, Muriel et al., 2011). These observations suggest that CAV1 could mediate the response to changes in substrate rigidity. To evaluate the potential contribution of CAV1 to ECM stiffness mechanotransduction, we performed RNA sequencing (RNA-seq) in wild-type (WT) and Cav1KO mouse embryonic fibroblasts (MEFs) cultured on rigid or compliant polyacrylamide hydrogels (GEO: GSE120514). Using Ingenuity Pathway Analysis (IPA) software and the Enrichr open-source tool (Chen et al., 2013, Kuleshov et al., 2016), we queried our datasets for canonical functional programs and Gene Ontology terms responsive to substrate rigidity, classifying them according to their specificity for WT or Cav1KO backgrounds (Figures S1C and S1D). This analysis identified a stiffness-induced increase in genes related to the regulation of actin cytoskeleton, focal adhesions, and cell junctions exclusively in WT cells.

**Figure 1:**
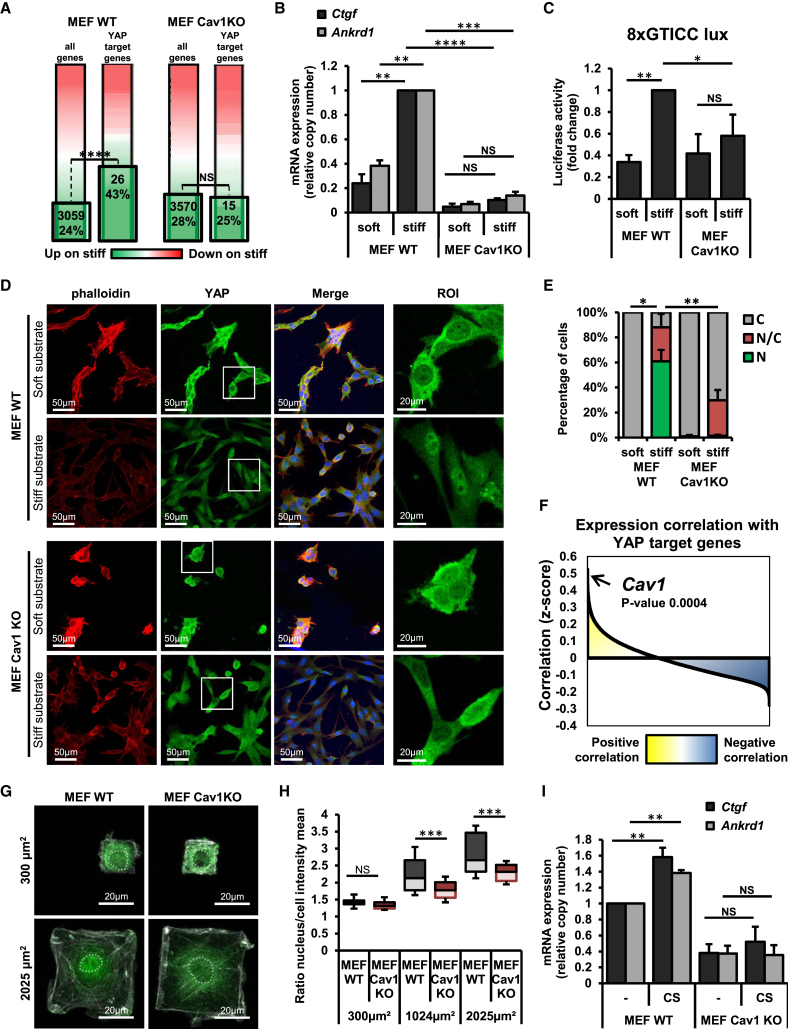
CAV1 Modulates YAP Activity (A) Variations in gene expression in the RNA-seq analysis between cells grown on stiff and soft substrates, showing all identified genes (left bar) and YAP-target genes alone (right bar). Genes significantly upregulated by ECM stiffness are boxed, and the enrichment for YAP target genes was analyzed using the Fisher exact test. Genes highlighted green are those that were upregulated on the stiff substrate, whereas those highlighted red were downregulated. (B) qRT-PCR analysis of *Ctgf* and *Ankrd1* expression in WT and Cav1KO MEFs grown on stiff and soft substrates for 24 hr. Data are normalized to WT cells grown on a stiff substrate. n = 3. (C) TEAD transcriptional activity in WT and Cav1KO MEFs expressing the 8xGTIIC-luciferase reporter and grown on stiff or soft substrates for 24 hr. Luciferase activity was measured and normalized as described in STAR Methods. Data are normalized to WT MEFs grown on stiff substrate. n = 4. (D) Confocal immunofluorescence images of YAP expression in WT and Cav1KO MEFs grown on stiff or soft substrate. F-actin was stained with fluorophore-conjugated phalloidin (red; left column), and nuclei were stained with Hoechst (blue in merged images; third column). The right column shows zoomed views of the YAP ROI (boxed in white in the YAP images). (E) Percentage of cells from analysis as in (D) with predominantly nuclear YAP (N), predominantly cytosolic YAP (C), or an even nuclear-to-cytosolic distribution (N/C). Randomly selected images from 3 independent experiments were analyzed (60–200 interphase cells per condition). (F) SEEK computational gene co-expression analysis, showing expression correlation (*Z* score) between YAP target genes and the rest of the genome. (G) Confocal immunofluorescence images of YAP in WT and Cav1KO MEFs plated on different micropatterns with a fibronectin-coated grid that allows cells to spread to a predefined size of 2,025 μm^2^ or 300 μm^2^. Nuclear contours are outlined with dotted gray lines. (H) ImageJ quantification of YAP subcellular distribution in cells plated on micropatterns of 3 grid sizes (300 μm^2^, 1,024 μm^2^, and 2,025 μm^2^). Data are presented as the nuclear to total cell staining intensities; 10–20 cells were analyzed from 2 biological replicates per condition. The boxplots show the median, 1^st^ and 3^rd^ quartiles, and 90^th^ and 10^th^ percentiles (whiskers). (I) qRT-PCR analysis of the YAP targets *Ctgf* and *Ankrd1* in cells subjected to cyclic mechanical stretching (CS; see STAR Methods) and unstretched cells. n = 4. Data in (B), (C), (E), and (I) are presented as means ± SEM; ^∗^p < 0.05, ^∗∗^p < 0.01, ^∗∗∗^p < 0.005, and ^∗∗∗∗^p < 0.0005. See also Figure S1 and Table S1.

To explore the molecular mechanisms mediating this effect of CAV1 on gene expression, we focused on YAP because this transcriptional cofactor is a prominent transcriptional driver of genes involved in cell adhesion and actin cytoskeleton organization (Stein et al., 2015) and is also positively regulated by mechanical cues such as ECM stiffness (Dupont et al., 2011). To assess whether YAP function was controlled by substrate stiffness in our system, we first analyzed the expression of a panel of 61 genes previously characterized as YAP targets in MCF10A and NIH 3T3 cells (Dupont et al., 2011, Zhao et al., 2008). A Fisher exact test confirmed statistically significant upregulation of endogenous YAP targets by ECM stiffness in WT cells, but not in Cav1KO cells (Figure 1A). This finding was supported by qRT-PCR analysis of the YAP targets *Ankrd1* and *Ctgf* (Figure 1B) and by orthogonal assays to monitor TEAD activity (Figure 1C) based on the 8xGTIIC luciferase reporter (Dupont et al., 2011). To explore the mechanism of this CAV1 dependency, we first studied YAP subcellular distribution (Figure 1D), which was classified as cytosolic (C), nuclear (N), or evenly distributed (N/C) (Figure 1E). As expected, YAP was predominantly nuclear in WT cells plated on stiff substrate and retained in the cytosol in cells plated on soft substrate. However, in Cav1KO MEFs, YAP was predominantly retained in the cytoplasm independently of substrate rigidity and compliance. Defective YAP nuclear localization in Cav1KO cells was confirmed by biochemical fractionation (Figure S1E). These results indicate that the positive regulation of YAP transcriptional activity by environmental rigidity is CAV1 dependent.

To rule out a cell-specific effect on YAP-CAV1 functional interactions, we used small interfering RNA (siRNA) duplexes to transiently knock down CAV1 in epithelial MDA-MB-231 human breast carcinoma cells. CAV1 silencing significantly decreased *Ctgf* and *Ankrd1* expression (Figure S1G). Moreover, qRT-PCR profiling of immortalized neonatal mouse hepatocytes revealed a similar reduction in YAP target gene expression in cells harvested from Cav1KO mice compared with those from WT mice (Figure S1H). To further assess the robustness of the CAV1-YAP interaction, we used the SEEK open-access resource (Zhu et al., 2015) to query known YAP target genes for coexpression patterns against the whole genome across extensive datasets from different tissues and cell lines (Figure 1F; Table S1). *Cav1,* whose mRNA levels highly correlate with its protein expression (Sonntag et al., 2014), showed one of the highest expression correlations with our YAP target list query (0.4994). These observations were upheld by the analysis of an independent dataset, generated by assessing the correlation between the expression of *Cav1* and the rest of the genome across 300 cell lines (Pellinen et al., 2018); in this analysis, 79% of YAP target genes correlated positively with *Cav1* expression and 11% correlated negatively (Figure S1G). Together, these observations suggest that CAV1-dependent regulation of YAP transcriptional activity is a general mechanism operating across different experimental systems.

Simultaneous siRNA-mediated knockdown of YAP and TAZ, to prevent potential compensatory mechanisms, effectively blocked expression of the canonical targets *Ctgf* and *Ankrd1* in WT MEFs (Figures S1J and S1K). Consistent with a pivotal role for CAV1 in the positive regulation of YAP, YAP/TAZ silencing in Cav1KO cells did not further decrease *Ctgf*, *Ankrd1*, and *Cyr61* expression. Notably, CAV1 absence did not alter total YAP protein levels (Figure S1J), suggesting that the relationship between CAV1 and YAP-dependent transcriptional programs relies on CAV1-dependent regulatory mechanisms upstream of YAP and not on the regulation of YAP protein expression.

Cell spreading modulates YAP activity such that YAP is predominantly nuclear in cells spread over large areas and cytosolic in cells with limited spreading (Dupont et al., 2011). Moreover, cell polarization and spreading in MEFs is controlled by CAV1 (Grande-García et al., 2007). To rule out the possibility that CAV1-dependent differences in YAP activity were secondary to differential cell spreading, we cultured MEFs on printed fibronectin micropatterns of fixed area and shape. As expected, YAP was predominantly cytosolic in WT MEFs spreading over small micropatterns, whereas growth on large micropatterns promoted a marked nuclear accumulation. This regulation was blunted in CAV1-deficient cells (Figures 1G and 1H). These observations confirm that CAV1-dependent YAP modulation is not an indirect consequence of changes in cell geometry.

To assess this relationship in the context of other mechanical cues, we evaluated the role of CAV1 in cell stretching, another established YAP-activating stimulus (Aragona et al., 2013, Codelia et al., 2014). Using a stretching device, we exposed cells to uniaxial cyclic strain. Stretching induced significant increases in *Ctgf* and *Ankrd1* expression in WT MEFs, but not in Cav1KO cells (Figure 1I), suggesting that CAV1 modulates YAP activity in response to different stimuli.

### CAV1-Dependent Regulation of YAP Is Independent of Hippo Kinase

**Figure 2:**
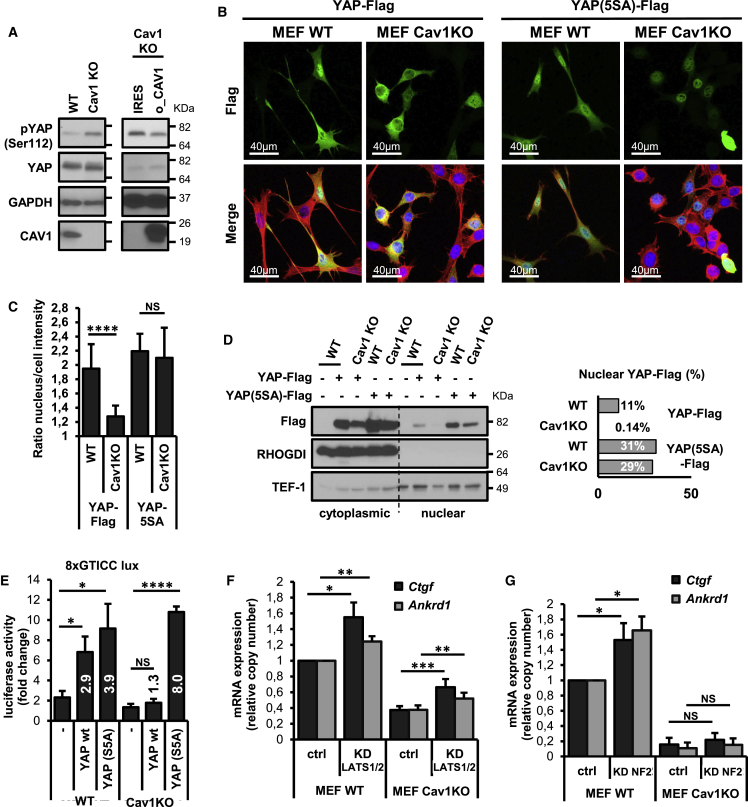
Hippo-Kinase-Independent YAP Serine Phosphorylation Determines Impaired Nuclear Translocation and Blunted YAP-Dependent Transcriptional Activity in Cav1KO Cells (A) Western blot of Ser112-phopshorylated YAP and total YAP in WT and Cav1KO MEFs and Cav1KO MEFs reconstituted with CAV1 (o_CAV1) or IRES-GFP. (B) Confocal immunofluorescence of cells transfected with YAP-FLAG or YAP(S5A)-FLAG and stained with anti-FLAG antibody (green), fluorophore-conjugated phalloidin (red), and Hoechst (blue). (C) FLAG distribution from analysis as in (B), represented as the ratio of nuclear-to-cytosolic intensities. n = 6–11. (D) Western blot analysis of FLAG subcellular distribution in MEFs transfected with YAP-FLAG or YAP(S5A)-FLAG followed by biochemical fractionation. RHO-GDI and Tef-1 were used as cytosolic and nuclear markers, respectively. The percentage of total YAP located in the nuclear fractions was quantified (right graph). (E) TEAD transcriptional activity in MEFs transfected with YAP-FLAG and YAP(S5A)-FLAG, measured by 8xGTICC-luciferase reporter assay. Data are normalized to growth on the soft substrate in each experiment. n = 3 (E). The fold-change with respect to untransfected cells is indicated above the bars. (F) qRT-PCR for *Ctgf* and *Ankrd1* expression in WT and CAV1 KO MEFs transfected with control or Lats1 and 2 siRNAs. Data are normalized to WT control. n = 10. (G) qRT-PCR for *Ctgf* and *Ankrd1* expression in WT and CAV1 KO MEFs transfected with control or NF2 siRNAs. Data are normalized to WT control. n = 4. Data are presented as mean ± SEM; ^∗^p < 0.05, ^∗∗^p < 0.01, ^∗∗∗^p < 0.005, and ^∗∗∗∗^p < 0.0005. See also Figure S2.

We observed that YAP phosphorylation at S112 was increased in Cav1KO MEFs, and this increase was partly blocked by exogenous CAV1 expression (Figure 2A). Previous reports proposed the existence of nuclear pools of S127-phosphorylated YAP in human cells (Wada et al., 2011), but our biochemical partition assays suggested that the phosphorylated form of the mouse homologous residue S112 is largely excluded from the nucleus in our cellular model (Figure S2A). YAP phosphorylation at serine 127 (S112 in mice) promotes the retention of this transcription factor in the cytosol (Basu et al., 2003, Zhao et al., 2007). We evaluated the involvement of YAP phosphorylation in CAV1-dependent regulation ectopically expressing YAP-FLAG and the non-phosphorylatable mutant YAP-5SA (Figure S2B). We transiently transfected these constructs into WT and Cav1KO MEFs and analyzed their subcellular distribution by both immunofluorescence and subcellular fractionation (Figures 2B–2D). In WT MEFs, FLAG-tagged WT YAP was predominantly nuclear but was mostly retained in the cytosol in Cav1KO MEFs. However, FLAG-tagged YAP-5SA accumulated in the nucleus in both WT and Cav1KO MEFs, suggesting that cytosolic retention of YAP in Cav1KO MEFs is at least partially dependent on its regulated phosphorylation. Constitutive nuclear translocation of YAP-5SA proteins in Cav1KO MEFs correlated with the rescue of its downstream transcriptional output. YAP-5SA nuclear accumulation in Cav1KO MEFs correlated with increased canonical YAP-TEAD transcriptional activity, assessed by 8xGTICC-luciferase reporter assay (Figure 2E). In contrast, whereas WT YAP enhanced TEAD activity in WT MEFs, it did not in Cav1KO MEFs, consistent with the cytosolic sequestration of WT YAP-FLAG and endogenous YAP in Cav1KO MEFs. These results were confirmed by qRT-PCR analysis (Figure S2C). It is important to note that while the fold increase was higher in Cav1KO cells, YAP-5SA overexpression in Cav1KO cells did not reach the levels observed in WT cells, suggesting that phosphorylation-independent mechanisms could also be involved. Our observations indicate that YAP serine phosphorylation has an impact on CAV1-dependent control of YAP localization and activity.

YAP serine phosphorylation can be mediated by the kinases LATS1 and LATS2 (Zhao et al., 2007). Knockdown of LATS1/2 increased *Ctgf* and *Ankrd1* mRNA expression and TEAD-driven luciferase reporter activity in both WT and Cav1KO cells, with comparable fold increases (Figures 2G, S2D, and S2E). To further evaluate the implication of Hippo canonical kinases in the differences observed between WT and Cav1KO cells, we analyzed the role of neurofibromin 2 (NF2). NF2 silencing led to an increase in *Ctgf* and *Ankrd1* expression in WT cells, but not in Cav1KO cells (Figures 2H and S2F), supporting the existence of alternative regulation upon suppression of LATS1/2 kinase activity by NF2 knockdown and precluding rescue of YAP activity in Cav1KO cells. Taken together, these results suggest that LATS1/2 kinases are not essential for CAV1-dependent YAP activity regulation.

### CAV1-Dependent Regulation of YAP Activity Is Exerted through the Control of Actin Polymerization

**Figure 3:**
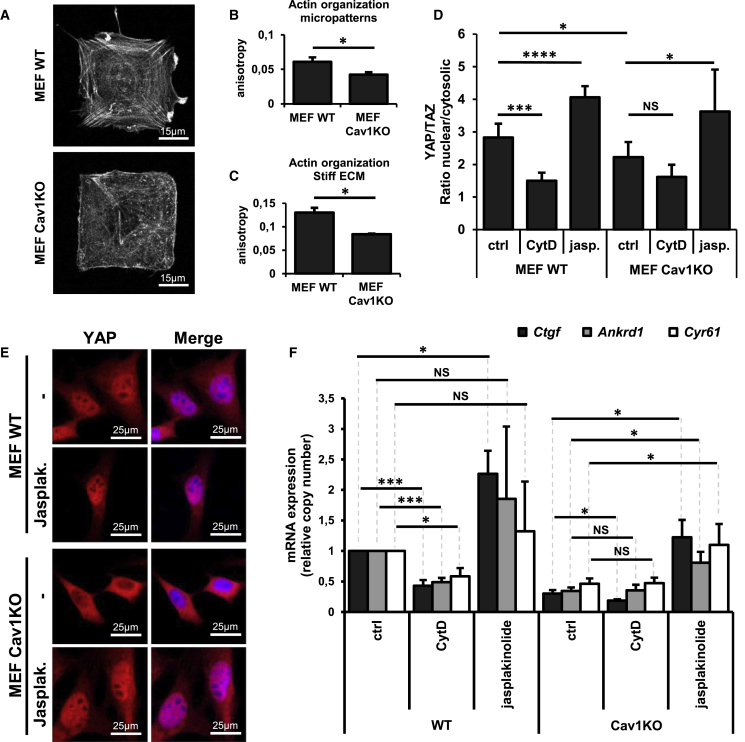
YAP Activity Defects in Cav1KO MEFs Are a Consequence of Defective Actin Polymerization (A) Confocal immunofluorescence images showing actin distribution in cells plated on large squared micropatterns (2,025 μm^2^). Actin was detected by staining with fluorophore-conjugated phalloidin. (B and C) Quantification of actin-fiber anisotropy (fiber order/organization) in WT and Cav1KO MEFs growing on squared micropatterns (n = 7–10; B) or on a stiff ECM (n = 3; C). (D) ImageJ quantification of YAP subcellular distribution in cells treated for 24 hr with 1 μM CytD, 0.05 μM jasplakinolide (Jasplak), or DMSO. Data were obtained from 3 to 8 independent experiments and are presented as means ± SD; ^∗^p < 0.05, ^∗∗∗^p < 0.005, and ^∗∗∗∗^p < 0.0005. (E) Confocal immunofluorescence of YAP in cells grown for 24 hr in the presence of 0.05 μM jasplakinolide or DMSO. Nuclei were detected with Hoechst (blue). (F) qRT-PCR of YAP target genes in MEFs treated for 24 hr with 1 μM CytD, 0.05 μM jasplakinolide, or DMSO (control). Data from jasplakinolide experiments (n = 5) and CytD experiments (n = 3) were normalized to WT controls. Data represent means ± SEM; ^∗^p < 0.05. See also Figure S3.

Since F-actin and RHO are necessary for YAP nuclear translocation and transcriptional activity (Dupont et al., 2011), we next checked whether changes in actin cytoskeleton and RHO signaling could explain the altered YAP regulation in Cav1KO MEFs. For the analysis of actin dynamics and architecture, WT and Cav1KO MEFs were cultured on large fibronectin micropatterns to ensure the same spreading area for both genetic backgrounds and thus exclude effects of spreading area on actin dynamics of cell-cell interaction, spreading, and cell shape (Figures 3A and 3B). Actin dynamics and architecture were also analyzed in cells cultured on stiff substrates (Figure 3C). Actin fiber organization was inferred by anisotropy analysis of microscopy images to measure the degree of departure from a homogeneous distribution toward an increasingly discrete intensity distribution (STAR Methods). Confirming CAV1 as a regulator of actin cytoskeleton organization, actin fibers were less organized in Cav1KO cells.

**Figure 4:**
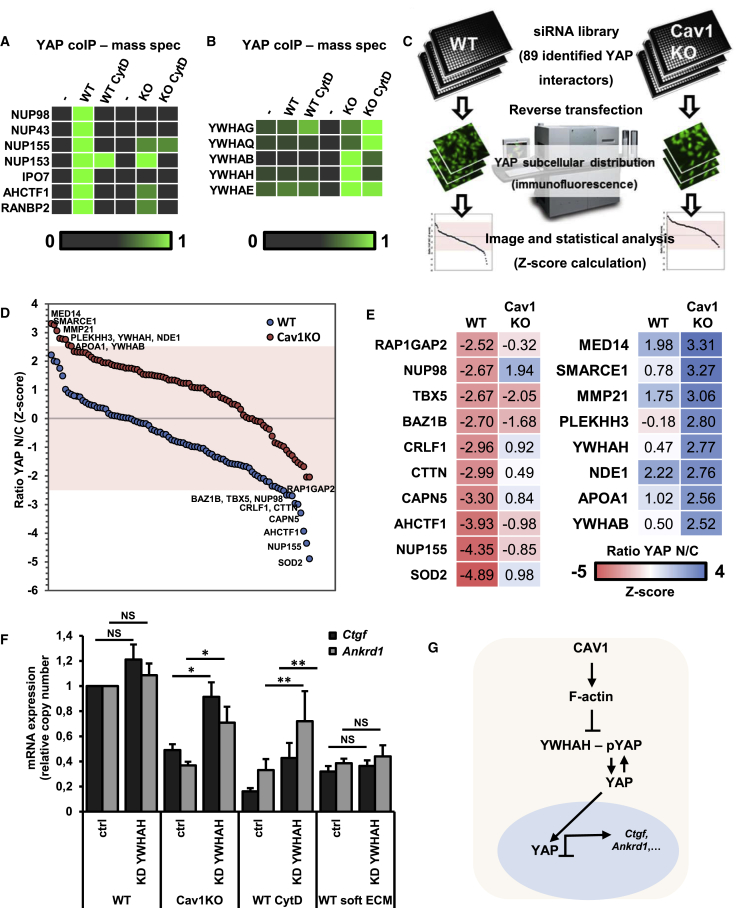
The YAP Interactome Is Altered in Cav1KO MEFs (A and B) Mass spectrometry analysis of YWHA proteins (A) and nuclear pore components and nucelocytosolic transporters (B) that co-immunoprecipitate with YAP in WT and Cav1KO MEFs treated with or without 1 μM CytD for 24 hr. The heatmap represents the relative number of counts per protein and condition. Negative controls (first and third column) were performed in parallel by omitting the primary anti-YAP antibody. n = 5. (C) siRNA library screening scheme for identifying YAP activity regulators. (D) Plot of mean *Z* scores of the YAP nuclear-to-cytosolic ratio for each individual siRNA in WT and Cav1KO MEFs. (E) Mean *Z* score of the YAP nuclear-to-cytosolic ratio in WT and Cav1KO cells after siRNA transfection for those genes whose *Z* scores are above 2.5 or below −2.5. n = 3. (F) qRT-PCR of *Ctgf* and *Ankrd1* in cells transfected with control or YWHAH siRNAs: WT and Cav1KO MEFs, WT cells treated for 24 hr with 1 μM CytD, and WT cells grown on soft substrate. n = 5. Data are presented as means ± SEM. ^∗^p < 0.05 and ^∗∗^p < 0.01. (G) Scheme of CAV1-YAP regulation. See also Figures S4 and S5, Table S2, and Data S1.

We assessed the potential contribution of actin dynamics to CAV1-dependent YAP regulation by using the actin polymerization inhibitor cytochalasin D (CytD) and jasplakinolide, an enhancer of F-actin actin polymerization (Holzinger, 2009, Prentki et al., 1979). CytD decreased stress fiber density (Figure S3A), reducing YAP nuclear accumulation and YAP target transcription throughput in WT cells to levels akin to those in Cav1KO cells (Figures 3D and S3A; see also Figures S3D and 4F). Conversely, jasplakinolide enhanced actin polymerization (Figures S3B and S3C), restored YAP nuclear translocation in Cav1KO MEFs to WT levels (Figures 3D and 3E; see also Figure S3D), and increased YAP target gene expression in both WT and Cav1KO cells (Figure 4F). A constitutively active DIAPH1 mutant (mDia1ΔN3), capable of boosting actin polymerization rates (Watanabe et al., 1999), significantly upregulated YAP target expression in Cav1KO cells (Figure S3E). These data strongly suggest that actin polymerization is a key component of the YAP regulatory machinery in our system. Altered actin dynamics in Cav1KO cells are the direct cause of the reduced YAP activity observed in this genetic background.

We next explored the contribution of RHO signaling to actin- and CAV1-dependent regulation of YAP using Y27632, a well-established inhibitor of the upstream kinase ROCK1/2. Exposure to Y27632 strongly reduced YAP target gene expression in WT cells, reproducing the effect of CytD (see Figure 3A); in contrast, Y27632 had only modest effects in Cav1KO cells (Figure S3F). Transient transfection with a constitutively active form of RHOA (RHOV14) that rescues RHO activity in Cav1KO MEFs (Goetz et al., 2011) further increased *Ctgf* and *Ankrd1* expression in WT cells but did not enhance YAP target gene expression in Cav1KO cells (Figure S3F). Our observations thus suggest that while RHO signaling is necessary for the CAV1-dependent positive mechanoregulation of YAP activity, it is not sufficient, since defective RHO cannot explain the deficient YAP activity in Cav1KO cells.

### Deficient YAP Activity in Cav1KO Cells Is Mediated by YWHAH-YAP Interaction

To characterize the molecular mechanisms underpinning the effect of CAV1-dependent actin dynamics on YAP activity, we profiled the YAP interactome by YAP immunoaffinity purification and mass spectrometry (MS) of control and CytD-treated WT and Cav1KO cells (Figure S5; Table S2). We identified several previously described YAP-interacting proteins: AMOTL2 (Zhao et al., 2011), POLR2A (Gavva et al., 1997), TBX5 (Rosenbluh et al., 2012), RUNX1 (Levy et al., 2008), 14-3-3 proteins, and known members of the Hippo pathway interactome (RBM15, CORO1C, DBN1, DOCK7, LIMA1, MTCL1, PKP4, RAD21, and SLMAP; Couzens et al., 2013). Interestingly, only WT cells showed interactions between YAP and nuclear pore and/or transport complexes, presumably reflecting effective nuclear translocation (Figure 4A). Conversely, both Cav1KO and CytD-treated cells were enriched for interactions with 14-3-3 proteins, which are reported to retain phosphorylated YAP in the cytosol (Dong et al., 2007) (Figure 4B).

To assess the contribution to YAP regulation of each component of these context-specific YAP interactomes, we carried out an image-based RNAi focused screen by knocking down 89 identified YAP interactors and comparing YAP subcellular distribution in Cav1KO and WT cells (STAR Methods; Figures 4C, 4D, S4A–S4C, and S5). Setting a stringent threshold of |Zq| > 2.5, we identified hits specific to WT cells for 10 genes, whose knockdown blunted YAP nuclear translocation (Figures 4D and 4E). These included siRNA pools targeting most nuclear pore components previously shown to selectively interact with YAP in WT cells (NUP155, NUP98, and AHCTF). Conversely, 8 hits were identified as specific to Cav1KO cells, and siRNA-mediated depletion of these genes enhanced YAP nuclear translocation. This second subset included two Cav1KO-specific YAP interactors, the 14-3-3-domain proteins YWHAH and YWHAB (Figures 4D, 4E, and S4D). We confirmed by western blot that YWHAH interacts preferentially with YAP in Cav1KO cells in CytD-treated WT cells compared with control WT cells (Figure S4E). Accordingly, efficient YWHAH siRNA-mediated depletion partially rescued the expression of YAP targets in Cav1KO cells and CytD-treated WT cells (Figures 4F, S4F, and S4G). Notably, this rescue was not effective in cells grown on soft substrates, indicating that additional mechanisms might be involved in this regulation. Taken together, these unbiased approaches suggest that CAV1 determines YAP activity through the control of actin dynamics, via mechanisms involving inhibition of the interaction between YAP and 14-3-3 proteins such as YWHAH (Figure 4G).

### YAP Is a Major Effector of ECM Remodeling Downstream of CAV1

**Figure 5:**
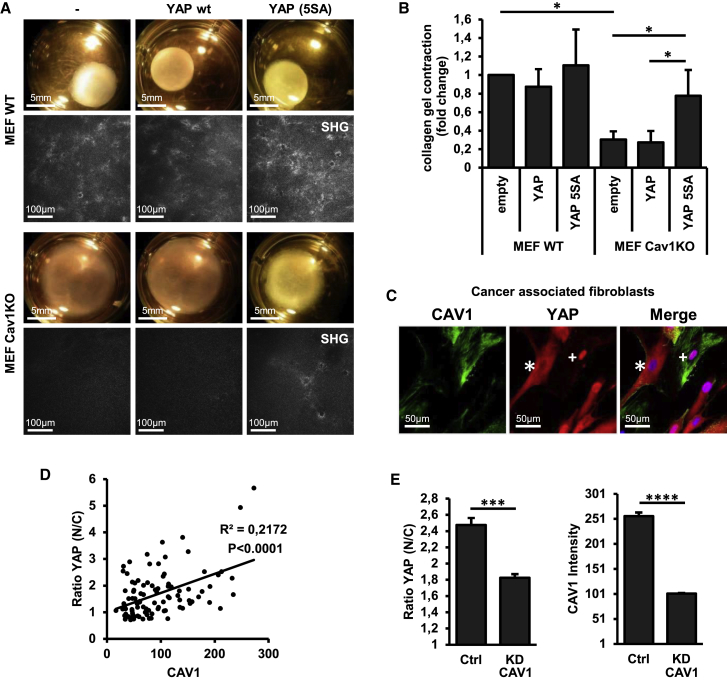
YAP Mediates the Effect of CAV1 in ECM Remodeling (A and B) Collagen gel retraction induced by MEFs transfected with YAP-Flag or YAP(S5A)-FLAG. (A) Cells were embedded in 3D collagen gels, and images were acquired 72 hr later to monitor gel retraction. Collagen organization was determined by second harmonic generation (SHG) microscopy (black and white images). Mock represents mock transfection. (B) Corresponding ImageJ quantification of gel contraction, measured as the fold change with respect to the contraction observed in mock-transfected WT cells. n = 3 experiments. (C) Confocal immunofluorescence images of CAV1 and YAP in cancer-associated fibroblasts (CAFs) extracted from the stroma of human pancreatic tumors and cultured *in vitro*. Nuclei were detected with Hoechst (blue). Symbols mark cells with low (^∗^) or high (^+^) CAV1 levels. (D) Plot of YAP nuclear-to-cytosolic ratio against CAV1 intensity for individual CAFs as in (C). N = 97. (E) Quantification of YAP subcellular distribution (left) and CAV1 levels (right) in CAFs transfected with CAV1 siRNAs or controls. CAV1 and YAP were detected by confocal immunofluorescence microscopy. A total of 4 independent experiments (n = 4) were analyzed (∼500 cells per experiment and condition) using Columbus. See STAR Methods for details. Data are presented as means ± SD in (B) and means ± SEM in (E); ^∗^p < 0.05, ^∗∗∗^p < 0.005, and ^∗∗∗∗^p < 0.0005.

YAP and caveolins are involved in a number of pathophysiological processes, such as liver regeneration, muscular dystrophy, and ECM remodeling (Bertrand et al., 2014, Calvo et al., 2013, Fernández et al., 2006, Goetz et al., 2011, Grijalva et al., 2014, Hagiwara et al., 2000, Minetti et al., 1998). We hypothesized that impaired ECM remodeling in CAV1-deficient cells (Goetz et al., 2011) might be caused by deficient YAP activity. To test this, we transfected Cav1KO cells with either non-phosphorylatable YAP-5SA (able to increase YAP transcriptional output in Cav1KO cells; see Figures 3G and 3H) or WT YAP. ECM remodeling was assessed by (1) collagen gel contraction assay and (2) quantitative image analysis of collagen fiber organization by second harmonic generation (SHG) microscopy (Figures 5A and 5B). As expected, ECM remodeling activity was blunted in Cav1KO MEFs. Interestingly, YAP-5SA overexpression restored the ability of these cells to remodel the matrix, whereas WT YAP was ineffective. Furthermore, we observed a clear correlation between CAV1 expression and YAP nuclear localization in human cancer-associated fibroblasts (CAFs) from pancreatic tumors (Figures 5C and 5D), and CAV1 silencing in these cells induced YAP cytosolic retention (Figure 5D), supporting a major role for a CAV1-YAP regulation in determining the activation state of stromal cell populations *in vivo*. Based on these observations, we propose that CAV1 and YAP nucleate a signaling pathway that drives ECM remodeling and stiffening.

### CAV1 Is Required for YAP Activation in Pancreatitis-Associated ADM

Pancreatitis causes tissue damage and desmoplasia, promoting the development of ADM and potentially contributing to PDAC onset and progression (Guerra et al., 2007). We chose pancreatitis as a model to study the potential contribution of CAV1-YAP regulation *in vivo*, because YAP is required for pancreatitis-induced ADM (Morvaridi et al., 2015) and CAV1 expression is upregulated in pancreatic cancer and it is associated with decreased survival (Chatterjee et al., 2015).

**Figure 6:**
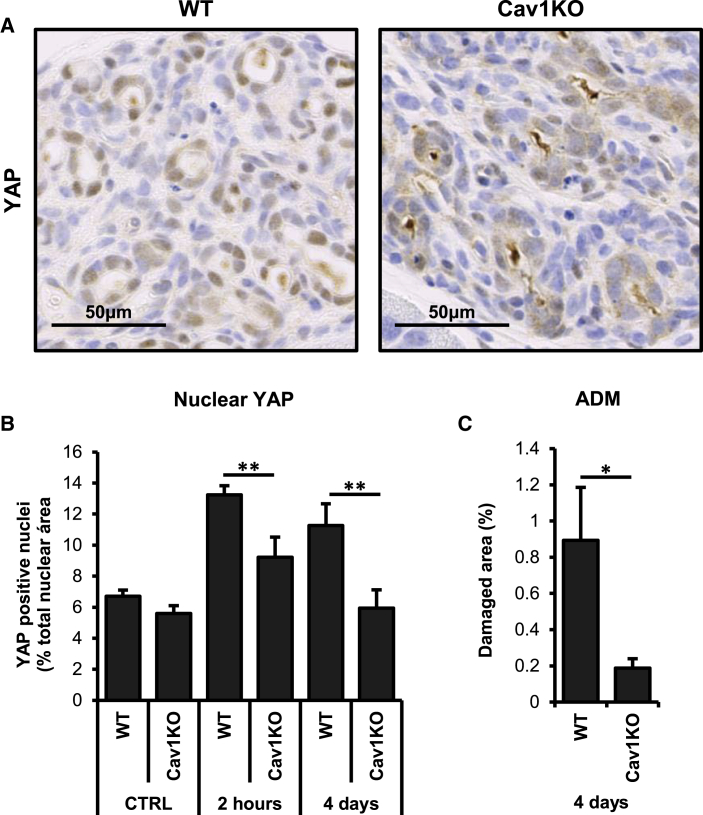
CAV1 Determines YAP Activation and ADM in Caerulein-Induced Acute Pancreatitis (A) Immunohistochemistry analysis of YAP expression in pancreatic tissue of WT and Cav1KO mice 4 days after caerulein treatment. (B) Quantification of the percentage of the nuclear area covered by YAP staining 2 hr and 4 days after caerulein treatment. n = 8. (C) Quantification of the percentage pancreatic area showing extensive ADM after 4 days of chronic pancreatitis in WT mice (n = 4) and Cav1KO mice (n = 3). Data in (B) and (C) are presented as means ± SEM; ^∗^p < 0.05, ^∗∗^p < 0.01. See also Figure S6.

Mild and reversible acute pancreatitis was induced in WT and Cav1KO mice by intraperitoneal administration of the cholecistokinin receptor agonist caerulein (Niederau et al., 1985). 2 hr and 4 days after caerulein treatment, nuclear YAP expression was significantly higher in WT preparations (Figures 6A and 6B). This correlates with an increase in the areas presenting extensive ADM and fibrosis, assessed by αSMA expression in pancreatic stellate cells 4 days after treatment (Figures 6C, S6A, and S6B). Taken together, these data suggest that CAV1 is required for YAP activation in the context of caerulein-induced pancreatitis and that this activation correlates with increased ADM in pancreatic tissue.

## Discussion

Our results identify CAV1 as an upstream regulator of YAP-dependent adaptive programs, working through mechanisms dependent on the control of actin dynamics. This CAV1-dependent control of YAP activity relies, at least in part, on the reversible phosphorylation of YAP, evidenced by the association of blunted YAP activity in Cav1KO cells with increased YAP phosphorylation and its rescued by exogenous expression of non-phosphorylatable YAP. We found no role for LATS1/2 in this mechano-dependent negative regulation, observing no YAP activity recovery either upon transient silencing of both kinases or upon silencing of NF2. YAP might also be a substrate for JNK and Abl (Codelia et al., 2014, Levy et al., 2008) or as-yet unidentified kinases that could be responsible for YAP regulation. Another possible explanation for increased YAP phosphorylation in the absence of CAV1 is a protection of phosphorylated YAP from dephosphorylation through interaction with 14-3-3 YWHA proteins. This interpretation is supported by our interactome profiling and systematic functional screening studies, which showed increased interaction of YWHA proteins with YAP in Cav1KO cells and specific rescue of YAP translocation upon their siRNA depletion. YAP retention in the cytosol upon interaction with YWHAH proteins led to deficient YAP transcriptional activity in these cells. Furthermore, YWHAH proteins positively control Yap expression (Figures S4G and 4F), adding a new level of complexity to the control of YAP activity. Our studies also identify several regulators of YAP nuclear translocation, including nuclear pore components and proteins involved in nucleocytosolic transport.

Changes in stromal stiffness and architecture can enhance tumor aggressiveness, promote resistance to therapy, and favor metastasis (Levental et al., 2009). During tumor progression, CAFs surrounding the tumor may favor an increase in the stiffness of the tumor mass. In CAFs, ECM stiffness itself is an activating cue, thus potentially enabling a mechanically driven feedforward loop in which YAP nuclear translocation is necessary for this activation (Calvo et al., 2013). CAV1 expression in CAFs correlates with higher remodeling capacity and facilitates tumor invasion (Goetz et al., 2011). Our results provide the first evidence of a functional connection between these nodes. 3D assays show that exogenous expression of a constitutively active YAP mutant reverts the impairment of ECM remodeling associated with CAV1 deficiency. This proposed CAV1-YAP regulation is therefore likely a significant driver of key events in tumor progression.

Pancreatitis is characterized by immune cell infiltration, interlobular and interacinar edema, and fibrosis and is a major risk factor for the development of pancreatic cancer (Yadav and Lowenfels, 2013). YAP contributes to acinar cell dedifferentiation in ADM and prevents the regeneration of injured areas (Gruber et al., 2016, Murakami et al., 2017). Furthermore, inflammation increases stiffening (Hidalgo, 2012), and the increased tissue stiffness in caerulein-induced acute pancreatitis could explain the differences in YAP activation between WT and Cav1KO mice. Our results thus support an important role for CAV1-YAP regulation *in vivo* and suggest a potential link between inflammation-induced stiffness and disease progression. These results suggest the interesting possibility that CAV1-YAP regulation could determine pancreatic cancer progression, since YAP is required for the initial stages of PDAC development (Gruber et al., 2016).

Our results demonstrate that CAV1 regulates YAP activity, determining the mechanical response to changes in ECM rigidity and other mechanical cues. CAV1-YAP regulation modulates pathophysiological processes such as ECM remodeling and the response to acute pancreatitis. These findings suggest that this regulation could determine the onset and progression of different physiological and pathological processes, such as tumor development, through multiple mechanisms.

## STAR★Methods

### Key Resources Table

**Table:** 

REAGENT or RESOURCE	SOURCE	IDENTIFIER
**Antibodies**		

Mouse monoclonal anti-YAP (63.7)	Santa Cruz Biotechnology	Cat# sc-101199, RRID: AB_1131430
Mouse monoclonal anti-TEF-1 (H-4)	Santa Cruz Biotechnology	Cat# sc-376113, RRID: AB_10988229
Rabbit monoclonal anti-CAV1 (D46G3) XP	Cell Signaling Tecnology	Cat# 3267S, RRID: AB_2275453
Rabbit monoclonal anti-14-3-3 η (D23B7)	Cell Signaling Tecnology	Cat# 5521S, RRID: AB_10829034
Monoclonal ANTI-FLAG® M2 antibody	Sigma-Aldrich	Cat# F3165, RRID: AB_259529
Mouse monoclonal anti-glyceraldehyde-3-phosphate dehydrogenase antibody	Millipore	Cat# MAB374, RRID: AB_2107445
Rabbit polyclonal anti-Phospho-YAP (Ser127) Antibody	Cell Signaling Technology	Cat# 4911S, RRID: AB_2218913
Rabbit polyclonal anti-Anti-LATS1 Antibody	Cell Signaling Technology	Cat# 9153S, RRID: AB_2296754
Rabbit polyclonal anti-Anti-LATS1 Antibody	Cell Signaling Technology	Cat#13646
Rabbit polyclonal anti-Anti-H3 Histone Antibody	Abcam	Cat# ab1791, RRID: AB_302613
Rabbit polyclonal anti-RHO GDIalpha (A-20) antibody	Santa Cruz Biotechnology	Cat# sc-360, RRID: AB_2227516
Rabbit monoclonal anti-YAP (D8H1X) XP	Cell Signaling Technology	Cat# 14074, RRID: AB_2650491
Rabbit Anti-Human Actin, Smooth Muscle Epitope Specific antibody	Thermo Fisher Scientific	Cat# RB-9010-P0, RRID: AB_149755
Alexa Fluor 488 Goat Anti Mouse IgG (H+L)	Molecular Probes	Cat# A-11029, RRID: AB_138404
Alexa Fluor 546 Goat Anti Mouse IgM (m chain)	Molecular Probes	Cat# A-21045, RRID: AB_2535714
Alexa Fluor 546 Goat Anti Rabbit IgG (H+L)	Molecular Probes	Cat# A-11035, RRID: AB_143051
Alexa Fluor 488 Goat Anti Rabbit IgG (H+L)	Molecular Probes	Cat# A-11034, RRID: AB_2576217
Goat anti-Mouse IgG (H+L) Secondary Antibody, HRP	Thermo Fisher Scientific	Cat# 31430, RRID: AB_228307
Goat anti-Rabbit IgG (H+L) Secondary Antibody, HRP	Thermo Fisher Scientific	Cat# 31460, RRID: AB_228341

**Bacterial and Virus Strains**		

N/A	N/A	N/A

**Biological Samples**		

N/A	N/A	N/A

**Chemicals, Peptides, and Recombinant Proteins**		

ROCK inhibitor Y27632	Sigma-Aldrich	Y0503; CAS Number 129830-38-2
CytochalasinD	Sigma-Aldrich	C8273; CAS Number: 22144-77-0
Jasplakinolide	Santa Cruz Biotechnology	sc-202191; CAS 102396-24-7
Caerulein	Sigma-Aldrich	C9026; CAS Number: 17650-98-5

**Critical Commercial Assays**		

Lipofectamine RNAiMAX Transfection Reagent	Invitrogene	Cat#10601435
Alexa Fluor 647 Phalloidin	Invitrogene	Cat# A22287, RRID:AB_2620155
Dual-Luciferase Reporter Assay System	Promega	Cat#E1910
RNAeasy micro kit	QIAGEN	Cat#74004
Omniscript RT kit	QIAGEN	Cat# 205111
Random primers	Promega	Cat#C1181
SYBR green	Applied Biosystems	Cat#4309155
G-Actin/F-actin *In Vivo* Assay Biochem Kit	Cytoskeleton	Cat#BK037

**Deposited Data**		

RNaseq data	This paper	GEO: GSE120514
Mass spectrometry data	Table S2	N/A

**Experimental Models: Cell Lines**		

WT and Cav1KO Mouse embryonic fibroblasts (MEF)	Razani et al., 2001	N/A
WT and Cav1KO Neonatal Hepatocytes (mouse)	Mayoral et al., 2010	N/A
MDA-MB-231 HTB-26 breast adenocarcinoma (human)	ATCC	Cat# HTB-26, RRID: CVCL_0062
HeLa CCL-2	ATCC	Cat# CCL-2, RRID: CVCL_0030
HeLa Cav1–GFP	Lukas Pelkmans laboratory	N/A
Primary pancreatic cancer associated fibroblasts (PanCAF)	Manuel Hidalgo Laboratory	N/A

**Experimental Models: Organisms/Strains**		

Mouse: Cav1KO C57BL/6	Drab et al., 2001	N/A

**Oligonucleotides**		

siRNA targeting sequence: Cav1 #1: GAGCUUCCUGAUUGAGAUU (sense sequence)	This paper	N/A
ON-Target Plus Smart-pool siRNAs; see Table S3	Dharmacon	N/A
Primers for qRT-PCR	Table S4	N/A

**Recombinant DNA**		

p2xFlag CMV2-YAP2	Oka et al., 2008	Addgene #19045
pCMV-flag YAP2 5SA	Zhao et al., 2007	Addgene #27371
8xGTIIC-luciferase	Dupont et al., 2011	Addgene #34615
pLVX-CMV-CherryFP-P2A-MetLuc	This paper	N/A
pLVX_shRNA2	Clontech	Cat#632179
CMVCherryFPP2A	Viral vectors Unit (CNIC)	N/A
pMetLuc reporter	Viral vectors Unit (CNIC)	N/A
pEGFP–mDia1(DeltaN3)	Ishizaki et al., 2001	N/A
pEGFP-RHO(V14)	del Pozo et al., 1999	N/A

**Software and Algorithms**		

qBase plus	Biogazelle	N/A
MATLAB (R2015a)	MATLAB	N/A
ImageJ	National Institutes of Health	https://imagej.nih.gov/ij/download.html
FibrilTool plug-in	Boudaoud et al., 2014	https://media.nature.com/original/nature-assets/nprot/journal/v9/n2/extref/nprot.2014.024-S3.txt
Ingenuity Pathway Analysis software	QIAGEN	N/A
Enrichr	Enrichr - Ma’ayan Laboratory - Computational Systems Biology	http://amp.pharm.mssm.edu/Enrichr/
Columbus Image Data Storage and Analysis System	PerkinElmer	http://www.perkinelmer.com/es/product/image-data-storage-and-analysis-system-columbus
Search-Based Exploration of Expression Compendium (SEEK)	Troyanskaya Functional Genomics Laboratory at Princeton University, 2014	http://seek.princeton.edu/
GraphPad Prism	GraphPad Software	https://www.graphpad.com/company/
Fisher exact test calculator	Social Science Statistics web site	http://www.socscistatistics.com/tests/fisher/Default2.aspx

**Other**		

pre-printed micropatterns	CYTOO	N/A
Fibronectin-coated 6-well plates for uniaxial stretching	FlexCell	Cat# UF-4001P
Flexcell FX-5000 Tension System	FlexCell	N/A
Protein G Sepharose 4 Fast Flow	GE Healthcare	Cat# GE17-0618-01

### Contact for Reagent and Resource Sharing

Further information and requests for reagents may be directed to, and will be fulfilled by the Lead Contact, Miguel Ángel del Pozo (madelpozo@cnic.es).

### Experimental Model and Subject Details

#### *In vivo* animal studies

Cav1KO C57BL/6 mice (Drab et al., 2001) were bred under specific pathogen-free conditions at the CNIC. Experiments were performed with 8-12-week-old males (Cav1KO and age-matched control littermates). All animal protocols (PROEX 097/18) were in accordance with Spanish animal protection law and were authorized by the corresponding local authority.

#### Cell culture

MEFs were isolated from WT and Cav1KO littermate mice, immortalized, and cultured as described (Razani et al., 2001). Neonatal hepatocytes from WT and Cav1KO littermates were isolated, phenotyped (Mayoral et al., 2010), and kindly provided by Dr. Martín-Sanz (IIBM Alberto Sols, Spain). The human MDA-MB-231 breast carcinoma and HeLa cell lines were obtained from ATCC. HeLa cells expressing CAV1–GFP were kindly provided by Lukas Pelkmans (ETH, Zürich, Switzerland). MEFs, hepatocytes and HeLa cells were grown in Dulbecco’s modified Eagle’s medium (DMEM) and MDA-MB231 cells were grown in DMEM/F-12 (GIBCO, Thermo Fisher Scientific [Waltham; Massachusetts, United States]); growth media were supplemented with 10% fetal bovine serum (FBS; GIBCO, Thermo Fisher Scientific and GE Healthcare Life Science HyClone [Little Chalfont, United Kingdom]) and 100 μg/ml penicillin and streptomycin (GIBCO, Thermo Fisher Scientific). Primary pancreatic cancer associated fibroblasts (PanCAF) were a gift from Manuel Hidalgo (Centro Nacional de Investigaciones Oncológicas, Spain). PanCAFs were grown in Roswell Park Memorial Institute medium (RPMI) supplemented with 20% FBS, 100 μg/ml penicillin and streptomycin, and 5% glutamine. All cells were maintained in a humidified atmosphere at 37 °C and 5% CO2.

### Method Details

#### Polyacrylamide matrices

Polyacrylamide gels with tuneable stiffness were prepared on glass coverslips as previously described (Tse and Engler, 2010). 3-aminopropyltrimethoxysilane (Sigma-Aldrich [St. Louis, Missouri, United States]) was applied over the surface of a coverslip using a cotton-tipped swab and another coverslip was treated with Sigmacote^®^ (Sigma-Aldrich). The coverslips were then washed thoroughly with sterilized water and dried. Acrylamide/bis-acrylamide solutions were prepared using appropriate concentrations to obtain stiff matrices (Young’s modulus ∼55 KPa) and soft matrices (∼200 Pa) as previously defined (Fischer et al., 2012). Polymerization initiators (0.05% w/v ammonium persulfate and 0.0005% v/v N,N,N′,N′-tetramethylethylenediamine [TEMED], final concentrations) were added to the bis-acrylamide mixture. A drop of this mixture was deposited on top of the silanized glass and covered with the sigmacote-treated coverslip; 183 μL was deposited for round coverslips (40mm diameter) and 50 μL for square coverslips (24x24mm). After polymerization, the upper coverslip was removed and the polyacrylamide surface was photo-activated by exposing the sulfo-SANPAH crosslinker (Sigma-Aldrich) to UV light. Finally, the surface was coated with fibronectin (5 μg/ml) for 1 h at 37°C. Fibronectin was then removed and cells were seeded at low confluence. Experiments were performed 24h after seeding.

#### Micropatterns

Glass slides with pre-printed micropatterns were purchased from Cytoo (Grenoble, France). Designs for customized patterns with specific grid sizes were described by Dr. Piccolo and colleagues (Dupont et al., 2011). Fibronectin coating was performed as specified by the supplier. Cells were plated, and 24 h later, fixed and stained following standard protocols.

#### Cell strain

24h after plating on fibronectin-coated 6-well plates (FlexCell [Burlington, North Carolina, United States]), cells were subjected to uniaxial cyclic stretching (0.7Hz, 8%–9% amplitude) for 24h on a programmable Flexcell® FX-5000™Tension System (FlexCell) under standard culture conditions.

#### Reagents and transfections

The ON-TARGET plus SMARTpool siRNAs were purchased from Dharmacon (Table S3; Lafayette, Colorado, United States), siRNA targeting human CAV1 was custom made (sense sequence: GAGCUUCCUGAUUGAGAUU. Cells were transfected with siRNAs at 20pmol/1000 cells using Lipofectamine® RNAiMAX (Invitrogene; Carlsbad, California, United States). Silencing was allowed to proceed for 48h before terminating the experiment.

p2xFlag CMV2-YAP2 was a gift from Dr. Sudol (Addgene plasmid # 19045; Cambridge, Massachusetts, USA) (Oka et al., 2008). pCMV-flag YAP2 5SA (Addgene plasmid # 27371) was a gift from Dr. Guan. 8xGTIIC-luciferase (Addgene plasmid # 34615) was a gift from Dr. Piccolo. The lentiviral backbone for pLVX-CMV-CherryFP-P2A-MetLuc was derived from pLVX_shRNA2 (Clontech; Mountain View, California, United States) and was provided by the CNIC Viral Vectors (VV) Unit. CMVCherryFPP2A was obtained from pRRL_CMV_CherryFP_P2A (provided by the CNIC VV Unit and cloned into pLVX). The Metridia luciferase (secretable form) was amplified from pMetLuc reporter (Clontech; also provided by the CNIC VV Unit) and cloned in-frame with the CherryFP-P2A peptide. pEGFP–mDia1(DeltaN3) and pcDNA3-HA-RHO(V14) were as described (del Pozo et al., 1999, Ishizaki et al., 2001). All transient transfections were by electroporation with 5 μg plasmid DNA and 35 μg UltraPure salmon sperm DNA solution (Sigma-Aldrich) at 350V and 550ohms for 10msec.

Drugs were added to cells 3h after plating, followed by incubation for a further 21h. The ROCK inhibitor Y27632 (Y0503) and cytochalasinD (C8273) were from Sigma-Aldrich. Jasplakinolide (sc-202191) was from Santa Cruz Biotechnology (Dallas, Texas, United States).

#### Antibodies

Monoclonal antibodies were sourced as follows: anti-YAP (sc-101199) and anti-TEF-1 (sc-376113) from Santa Cruz Biotechnology; anti-CAV1 XP (#3267) and anti-YWHAH (14-3-3 η (D23B7); #5521) from Cell Signaling (Danvers, Massachusetts, United States); anti-Flag M2 (F-3167) from Sigma-Aldrich; and anti-glyceraldehyde-3-phosphate dehydrogenase (MAB374) and anti-cortactin (p80/85, clone 4F11) from Millipore (Burlington, Massachusetts, United States). Polyclonal antibodies to phospho-YAP (Ser127) (#9411), LATS1 (#9153), and LATS2 (#13646) were from Cell Signaling; anti-Histone H3 (ab1791) was from Abcam (Cambridge, United Kingdom); and anti-RHO GDI (sc-360) was from Santa Cruz Biotechnology. For immunohistochemistry, we used anti-YAP (D8H1X) XP from Cell Signaling (#14074) and αSMA from Thermo Fisher Scientific (RB-9010-P0).

#### Immunofluorescence microscopy

For immunofluorescence procedures, cells were fixed in paraformaldehyde 4% (w/v) at 37°C for 10 minutes, permeabilized, blocked with 0.2% Triton X-100 in BSA 1% (w/v) for 10 min, and then immunostained with specific antibodies for 1h. Alexa647 phalloidin and Alexa546- and Alexa488-labeled secondary antibodies were from Invitrogen. Images were acquired either on a Zeiss LSM700 confocal microscope or an Opera automated confocal microscope (PerkinElmer; Waltham, Massachusetts, United States).

#### Subcellular fractionation

For subcellular fractionation the cells were lysed (10 mM HEPES, pH 7.6, 10 mM KCl, 0.1 mM EDTA, 0.1 mM EGTA, 0.5 mM DTT, 100 mM phenylmethylsulfonyl fluoride, protease inhibitor cocktail [Roche], and 0.05% NP-40). Nuclear and cytoplasmic fractions were separated by centrifugation. The cytosolic fraction was precipitated with acetone and nuclei were lysed (20 mM HEPES, pH 7.6, 0.4 M NaCl, 1 mM EDTA, 1 mM EGTA, 1 mM DTT, 0.75 mM spermidine, 0.15mM spermine,100 mM phenylmethylsulfonyl fluoride, and protease inhibitor cocktail [Roche]) and centrifuged at 13000 rpm to remove the DNA. Both fractions were eluted with sample buffer and analyzed by western blotting.

#### Immunoprecipitation

For immunoprecipitation, cells were lysed (50mM Tris-HCl at pH8, 100mM NaCl, 1% Triton X-100 [Sigma-Aldrich], 10% glycerol, 1mMMgCl2, 2mM PMSF, protease inhibitor cocktail [Roche]). Cell lysates were centrifuged for 10 min at 4°C. Supernatants were mixed with the specific antibody or control IgG for 2h, and protein G–agarose beads were added for a further 2h. Beads were washed with washing buffer (50mM Tris-HCl pH7.5, 150mM NaCl, 1mM EDTA, 0.25% gelatin, 0.1% NP-40 [Sigma-Aldrich]) and processed for mass spectrometry or western blotting.

#### Immunoblotting

For western blotting, immunoprecipitated proteins were eluted with sample buffer and analyzed by western blotting on nitrocellulose membranes (Amersham Pharmacia Biotech, UK) with primary and secondary HRP-conjugated antibodies using standard protocols. Proteins were detected by enhanced chemiluminescence (Amersham Life Sciences; Arlington Hts, Illinois, United States). Nuclear and cytosolic subcellular fractions were prepared as described (Navarro-Lérida et al., 2015).

#### Image analysis

YAP subcellular distribution was analyzed with the Columbus Image Data Storage and Analysis System (Perkin Elmer) or imageJ. Nuclei were segmented using the Hoechst signal (Figure S4B). Mitotic and aberrant nuclei were then eliminated based on Hoechst intensity and nuclear roundness and area. Cells located at the image borders were also eliminated. The cytosol was segmented growing the nuclear segmentation (Figure S4C). The cytosolic ROI for cytosolic YAP intensity calculation was built as a 4 pixel ring of cytoplasm grown radially from the segmented nuclear border (Figure S4C). Finally, the ratio between nuclear and cytosolic YAP was calculated.

#### Second harmonic generation (SHG) imaging

Fibrillary collagen in non-fixed cell-embedded collagen gels was imaged using the SHG technique (Chen et al., 2012) with a Zeiss LSM780 multiphoton microscope (Carl Zeiss Microscopy; Jena, Germany) fitted with a short pulse laser.

#### Luciferase assay

Luciferase assays to monitor TEAD transcriptional activity with the 8xGTIIC-luciferase reporter were as described (Mahoney et al., 2005). Cells were transiently co-transfected with 8xGTIIC-luciferase (product: firefly luciferase) and pLVX-CMV-CherryFP-P2A-MetLuc (product: Metridia luciferase [MelLuc], which is secreted to the medium). Luciferase activity was monitored with the Dual-Luciferase® Reporter Assay System (Promega; Madison, Wisconsin, United States) in an ORION II microplate luminometer (Titertek Berthold; Bad Wildbad, Germany). Firefly luciferase was quantified in cell lysates by adding Luciferase Assay Reagent II (LARII), and MetLuc was quantified in culture medium by adding Stop & Glo reagent. Firefly luciferase activity was normalized to MetLuc activity to control for variability in transfection efficiency across samples.

#### Collagen contraction assay

Contraction assays to monitor matrix remodeling were as described (Goetz et al., 2011, Orimo et al., 2005). Briefly, 1.5 × 10^5^ MEFs were included in a collagen type I gel (PureCol, Sigma-Aldrich; 1.5mg/ml, 500 μL total volume) in an Ultra-Low Attachment 24-well plate (Corning; Corning, New York, United States). After gel polymerization, normal culture medium was added, and collagen gel borders were detached from the border of the plate. Gels were cultured at 37 °C, 5% CO_2_ for 48h. Gel contraction was monitored by quantifying the gel surface area on photographs with ImageJ. The fold-change with respect to the contraction observed in a control condition was calculated for each sample.

#### Acute pancreatitis induction

Acute pancreatitis was induced by caerulein treatment as described (Guerra et al., 2007). Before the experiment, mice were starved for 12h with unrestricted access to drinking water. Acute pancreatitis was induced by 7 intraperitoneal injections of caerulein (Sigma-Aldrich) dissolved in PBS; injections were given at 1-h intervals on 2 consecutive days at a dose of 50 μg caerulein /kg body weight per injection. Control animals received injections of PBS only. At defined intervals, animals were sacrificed and the pancreas excised for immunohistochemical analysis. Immunostained preparations were scanned with Hamamatsu Nanozoomer 2.0 RS (Hamamatsu, Japan) and digitized with NDP.scan 2.5. Images were viewed and quantified with NDP.analyzer and NDP.view2 (Hamamatsu; Hamamatsu City, Japan).

#### Real-time quantitative PCR

RNA was extracted from cell samples with the RNAeasy micro kit (QIAGEN; Hilden, Germany). For each sample, 1 μg RNA was reverse transcribed using the Omniscript RT kit (QIAGEN) and random primers (Promega). qPCR was performed with SYBR green (Applied Biosystems; Foster City, California, USA) Appropriate negative and positive controls were used (Hellemans et al., 2007). Results were normalized to endogenous GAPDH and HPRT1 expression using qBase plus. Primer sequences were summarized in Table S4.

#### RNA-Seq analysis

Next generation sequencing experiments were performed at the CNIC Genomics Unit. Total RNA was extracted as for qRT-PCR (see above). RNA integrity was determined with an Agilent 2100 Bioanalyzer (Agilent Technologies; Santa Clara, California, United States). Two RNA samples per condition were analyzed by single read (SR) sequencing in an Illumina HiSeq 2500 System (Illumina; San Diego, California, United States). Data were analyzed in the CNIC Bioinformatics Unit. Enrichment analysis was conducted using Ingenuity Pathway Analysis software (IPA, QIAGEN) and the Enrichr web tool.

#### Actin fiber organization analysis

Actin fiber organization was imaged and quantified in MATLAB (R2015a) and ImageJ (1.51a x64). Fiber order/parallelism in a ROI was measured with a custom multiscale anisotropy analysis script based on the FibrilTool plug-in for ImageJ (Boudaoud et al., 2014); this tool analyzes the structural information embodied by the eigenvalues and eigenvectors of a nematic tensor. Iterative performance of this analysis in a collection of image patches of different sizes gives information about the organization of structures at multiple scales and locations. The anisotropy value of each pixel in the original image is set to the mean anisotropy score obtained for each processed patch containing the pixel. The patch collection is created by dividing the image into NxN nonoverlapping subimages, with $N\in \left\{2^{i}|i=0,1,2\ldots \right\}\cup \left\{3^{i}|i=0,1,2\ldots \right\}$ defining each iteration. The final multiscale anisotropy score is the mean of all the anisotropy values of the pixels in the image. This measurement can be constrained to a specific area of interest by using only specific pixels determined by prior segmentation, a useful way of avoiding interference from background or other structures that could bias the results. In micropattern experiments, the inputs were cropped images of the cytoplasmic area of cells (241x261 pixels), avoiding background and membranes as much as possible; no prior area restriction was defined, and the minimum size of patches was fixed at 10x10 pixels.

#### Mass spectrometry analysis

Protein G–agarose beads bound to immunoprecipitated proteins were incubated at room temperature for 2h in a 60 μL volume of 2 M urea, 50 mM Tris-HCl pH 8.5, and 10 mM TCEP with gentle vortexing. Iodoacetamide (7 μL, 500 mM) was then added and the incubation continued in the dark. After dilution to 0.5 M urea with ammonium bicarbonate, 3 μg of trypsin were added and samples were incubated for 6-8 h at 37°C. Samples were then acidified to 1% TFA, and the supernatants were desalted on C18 minispin columns (The Nest Group; Southborough, MA, USA) and dried down for further analysis. Experiments were performed with 5 independent replicates. Peptides were analyzed by LC-MS/MS using a C-18 reversed phase nano-column (75 μm I.D. x 50 cm, 2 μm particle size, Acclaim PepMap RSLC, 100 C18; Thermo Fisher Scientific) in a continuous acetonitrile gradient consisting of 0%–32% B over 80 min, 50%–90% B over 3 min at 50°C (A = 0.1% formic acid; B = 80% acetonitrile, 0.1% formic acid). Peptides were eluted from the nanocolumn at a flow rate of 200 nL/min to an emitter nanospray needle for real-time ionization and peptide fragmentation in a QExactive HF mass spectrometer (Thermo Fisher Scientific). The chromatographic run analyzed an enhanced FT-resolution spectrum (70,000 resolution) followed by the MS/MS spectra from the 15 most intense parent ions. Dynamic exclusion was set at 40 s. For peptide identification, all spectra were analyzed with Proteome Discoverer (version 2.1.0.81, Thermo Fisher Scientific) using SEQUEST-HT (Thermo Fisher Scientific). For searching the Uniprot proteome database containing all sequences from mouse and frequently observed contaminants (April 27, 2016; 48644 entries), the following parameters were selected: trypsin digestion with 2 maximum missed cleavage sites; precursor and fragment mass tolerances of 2 Da and 0.02 Da, respectively; carbamidomethyl cysteine as a fixed modification; and methionine oxidation as a dynamic modification. Peptides were identified by the probability ratio method (Martínez-Bartolomé et al., 2008), and false discovery rate (FDR) was calculated using inverted databases and the refined method (Navarro and Vázquez, 2009) with an additional filtering for a precursor mass tolerance of 15 ppm (Bonzon-Kulichenko et al., 2015). Proteins were quantified for each condition based on the number of scans/peptides identified at 1% FDR.

#### Image-based siRNA screening

The smart-pool siRNA library for selected YAP interactors detected by mass spectrometry was purchased from Dharmacon. Four different sequences per each gene were used. SiRNAs were transfected by reverse transfection in 384-well plates. Cells were fixed and stained for YAP detection 48h post transfection as described previously. Immunofluorescence images were acquired with an Opera automated confocal microscope (PerkinElmer). Three replicates were performed, with four wells per siRNA in each replicate. Two different ON-traget nontargeting siRNA controls were used. Transfection efficiency was validated by transfection with INCENP siRNA, which promotes the appearance of multinucleated cells and cells with aberrant nuclei (Figure S4A). YAP nucleo:cytosolic ratios were calculated using Columbus as described above (Image analysis section), and Z-scores were calculated as Z = (x – control mean)/control standard deviation. The mean Z-score of the three replicates was calculated.

#### SEEK analysis

SEEK (http://seek.princeton.edu) is a computational coexpression gene search tool (Zhu et al., 2015). We queried this web tool with the list of previously published YAP target genes (Dupont et al., 2011) and used all human expression datasets from tissue samples and cell lines included in SEEK for coexpression analysis. The program gives a ranked list of genes ordered from the strongest positive correlation with the query to the weakest. With Enrichr (Chen et al., 2013, Kuleshov et al., 2016), we analyzed the enrichment in KEGG annotated pathways and gene ontology (GO) terms of the 200 genes showing the highest positive coexpression with YAP targets.

### Quantification and Statistical Analysis

Statistical details of experiments are reported in Figure Legends. Significance was evaluated by paired Student’s t test, using GraphPad Prism. Differences were considered statistically significant at ^∗^p < 0.05, ^∗∗^p < 0.01, ^∗∗∗^p < 0.005, and ^∗∗∗∗^p < 0.0005. YAP-target gene enrichment on stiff versus soft substrates in the RNA-Seq analysis was compared by the Fisher exact test using an online Fisher exact test calculator (http://www.socscistatistics.com/tests/fisher/Default2.aspx).

### Data and Software Availability

The accession number for the RNA-seq data reported in this paper is GEO: GSE120514.

## Supplemental Information

Document S1. Figures S1–S6 and Tables S3 and S4

Table S1. Coexpression of YAP Target Genes with the Rest of the Genome Genes, Analyzed across Extensive Datasets from Different Tissues and Cell Lines, Related to Figure 1

Table S2. YAP Interactors Identified in WT and Cav1KO MEFs Untreated or Treated with CytD, Related to Figure 4

Data S1. Automated Image Analysis Script, Related to Figure 4

Document S2. Article plus Supplemental Information
