# Caveolin-1 Modulates Mechanotransduction Responses to Substrate Stiffness through Actin-Dependent Control of YAP

**DOI:** 10.1016/j.celrep.2019.01.090

**Published:** 2019-02-05

**Authors:** Roberto Moreno-Vicente, Dácil María Pavón, Inés Martín-Padura, Mauro Català-Montoro, Alberto Díez-Sánchez, Antonio Quílez-Álvarez, Juan Antonio López, Miguel Sánchez-Álvarez, Jesús Vázquez, Raffaele Strippoli, Miguel A. del Pozo

(Cell Reports *25*, 1622–1635.e1–e6; November 6, 2018)

In the originally published version of this article, Figure 1D showed four merged channel accessory images, of which two of them had been mistakenly swapped with each other. While this mistake does not affect at all the interpretation and quality assessment of the experiment, the original image has been replaced by a corrected version in the online article. Both the original and the corrected version of the panel are also displayed below.

The authors regret this error.

**Figure:**
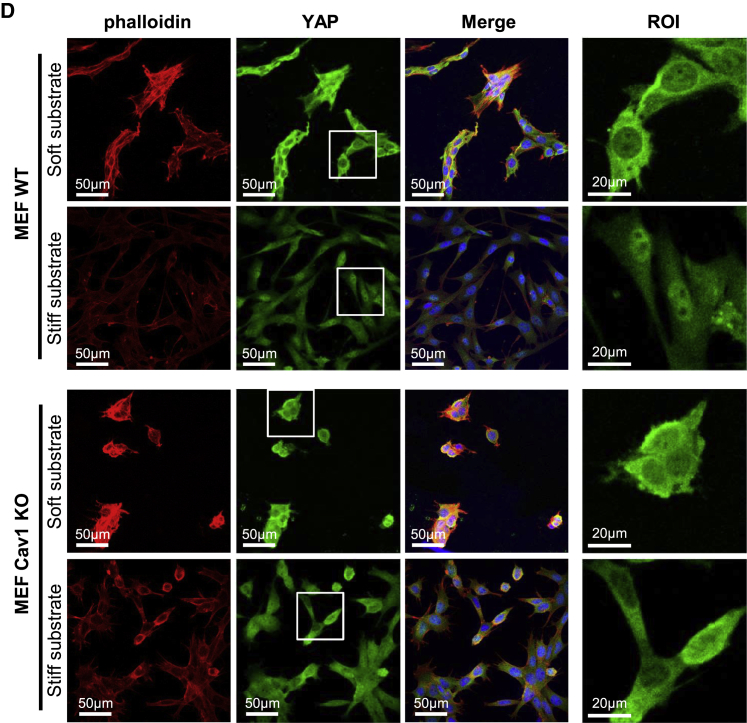
Figure 1D. CAV1 Modulates YAP Activity (Corrected)

**Figure:**
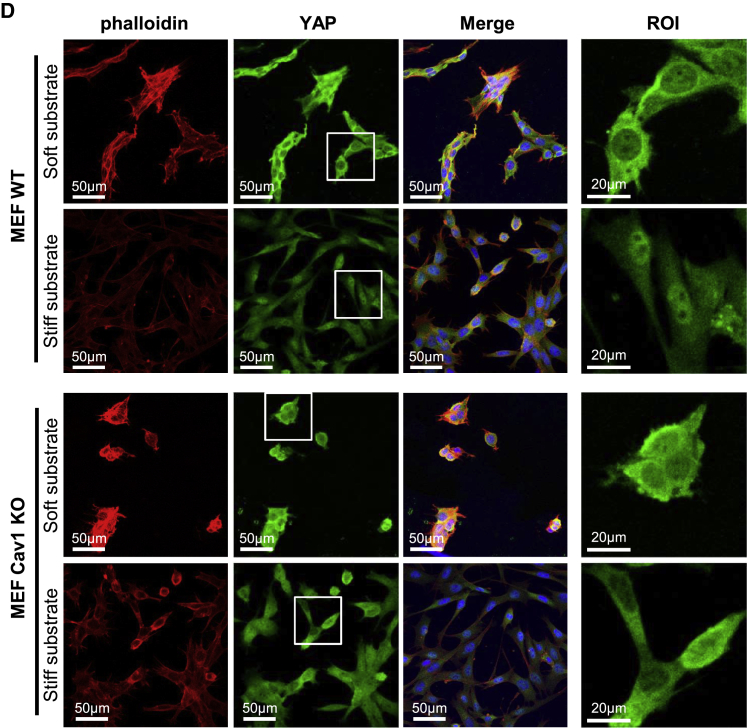
Figure 1D. CAV1 Modulates YAP Activity (Original)

