# Supplementary material for: Caveolin-1 Modulates Mechanotransduction Responses to Substrate Stiffness through Actin-Dependent Control of YAP
Source: Cell Rep. 2018 Nov 6;25(6):1622–1635.e6. doi: 10.1016/j.celrep.2018.10.024 (PMC6231326; doi:10.1016/j.celrep.2018.10.024)
Supplement: Document S1. Figures S1–S6 and Tables S3 and S4 [file mmc1.pdf]

Cell Reports, Volume *25*

Supplemental Information

Caveolin-1 Modulates Mechanotransduction Responses to Substrate Stiffness through Actin-Dependent Control of YAP

Roberto Moreno-Vicente, Dácil María Pavón, Inés Martín-Padura, Mauro Català-Montoro, Alberto Díez-Sánchez, Antonio Quílez-Álvarez, Juan Antonio López, Miguel Sánchez-Álvarez, Jesús Vázquez, Raffaele Strippoli, and Miguel A. del Pozo

Figure S1

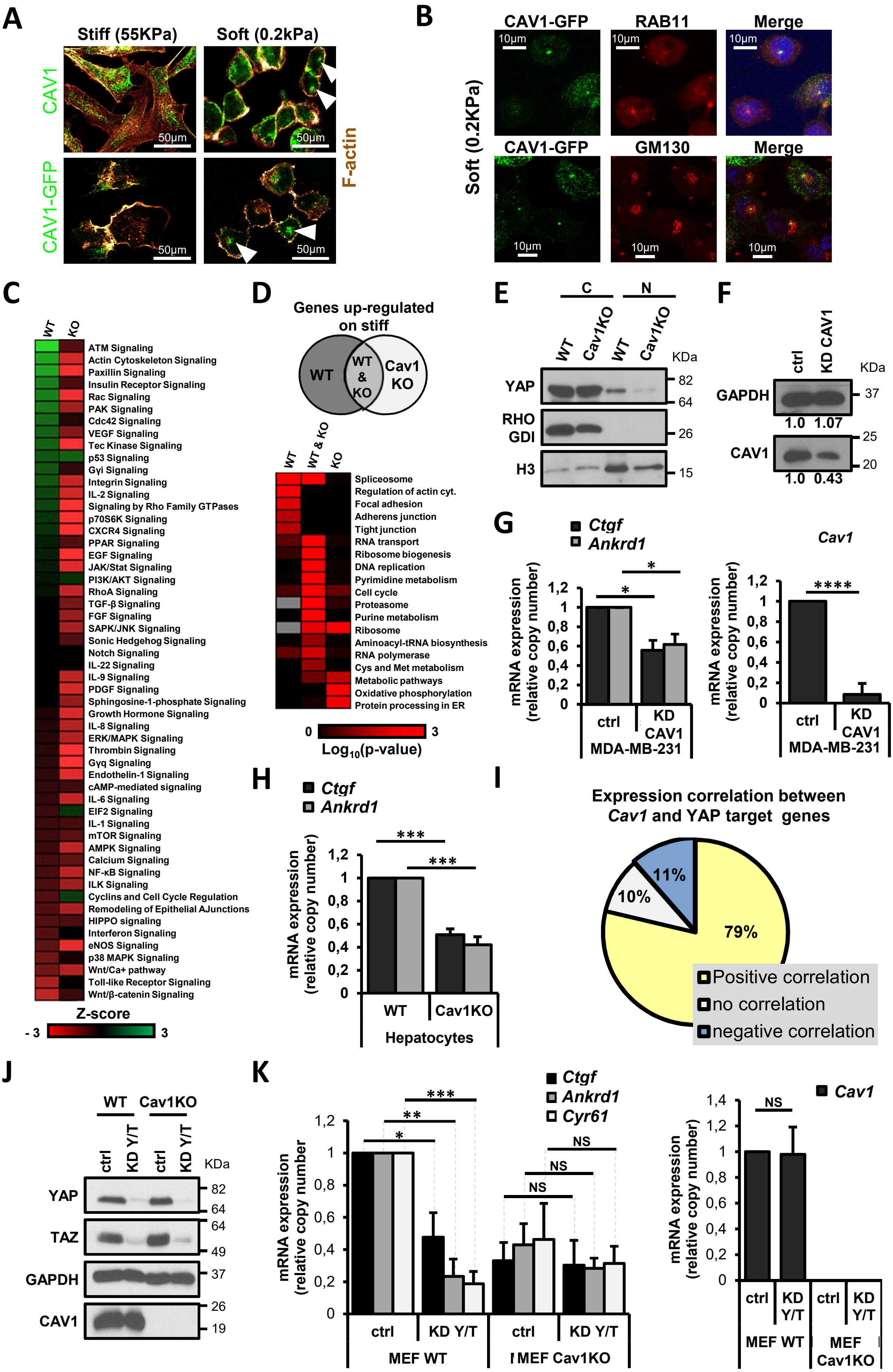

A
Stiff (55KPa)
Soft (0.2kPa)
CAV1
CAV1-GFP
F-actin
50µm
B
Soft (0.2KPa)
CAV1-GFP
RAB11
Merge
GM130
10µm
C
WT
KO
ATM Signaling
Actin Cytoskeleton Signaling
Paxillin Signaling
Insulin Receptor Signaling
Rac Signaling
PAK Signaling
Cdc42 Signaling
VEGF Signaling
Tec Kinase Signaling
p53 Signaling
Gγi Signaling
Integrin Signaling
IL-2 Signaling
Signaling by Rho Family GTPases
p70S6K Signaling
CXCR4 Signaling
PPAR Signaling
EGF Signaling
JAK/Stat Signaling
PI3K/AKT Signaling
RhoA Signaling
TGF-β Signaling
FGF Signaling
SAPK/JNK Signaling
Sonic Hedgehog Signaling
Notch Signaling
IL-22 Signaling
IL-9 Signaling
PDGF Signaling
Sphingosine-1-phosphate Signaling
Growth Hormone Signaling
IL-8 Signaling
ERK/MAPK Signaling
Thrombin Signaling
Gγq Signaling
Endothelin-1 Signaling
cAMP-mediated signaling
IL-6 Signaling
EIF2 Signaling
IL-1 Signaling
mTOR Signaling
AMPK Signaling
Calcium Signaling
NF-κB Signaling
ILK Signaling
Cyclins and Cell Cycle Regulation
Remodeling of Epithelial AJunctions
HIPPO signaling
Interferon Signaling
eNOS Signaling
p38 MAPK Signaling
Wnt/Ca+ pathway
Toll-like Receptor Signaling
Wnt/β-catenin Signaling
Z-score
- 3
3
D
Genes up-regulated on stiff
WT
WT & KO
Cav1 KO
KO
Spliceosome
Regulation of actin cyt.
Focal adhesion
Adherens junction
Tight junction
RNA transport
Ribosome biogenesis
DNA replication
Pyrimidine metabolism
Cell cycle
Proteasome
Purine metabolism
Ribosome
Aminoacyl-tRNA biosynthesis
RNA polymerase
Cys and Met metabolism
Metabolic pathways
Oxidative phosphorylation
Protein processing in ER
0
3
Log10(p-value)
E
C
N
WT
Cav1KO
KDa
YAP
RHO GDI
H3
82
64
26
15
F
ctrl
KD CAV1
KDa
GAPDH
37
1.0
1.07
25
CAV1
20
1.0
0.43
G
Ctgf
Ankrd1
Cav1
mRNA expression (relative copy number)
1,2
1
0,8
0,6
0,4
0,2
0
*
*
****
ctrl
KD CAV1
MDA-MB-231
H
Ctgf
Ankrd1
***
***
mRNA expression (relative copy number)
WT
Cav1KO
Hepatocytes
I
Expression correlation between Cav1 and YAP target genes
11%
10%
79%
Positive correlation
no correlation
negative correlation
J
WT
Cav1KO
ctrl
KD Y/T
KDa
YAP
TAZ
GAPDH
CAV1
82
64
64
49
37
26
19
K
Ctgf
Ankrd1
Cyr61
mRNA expression (relative copy number
1,2
1
0,8
0,6
0,4
0,2
0
***
**
*
NS
NS
NS
ctrl
KD Y/T
MEF WT
MEF Cav1KO
Cav1
1,4
NS
mRNA expression (relative copy number)
MEF WT
MEF Cav1KO

Figure S1. Cav1 controls the expression of cytoskeletal regulatory genes and YAP targets. (Related to Figure 1) (A) Conflocal immunofluorencence images of CAV1 and phalloidine staining in HeLa cells (upper panels) and CAV1-GFP expressing cells (lower panels) grown on stiff or soft substrates for 24 hours. Arrowheads mark the intracellular accumulation of CAV1 in soft conditions. **(B)** Conflocal immunofluorencence images of CAV1-GFP, RAB11 (recycling endosome marker) and GM130 (Golgi marker). **(C)** Computational analysis using IPA software to detect canonical pathways regulated by matrix stiffness. mRNA expression in cells on rigid (55KPa) or compliant (0.22KPa) polyacrylamide hydrogels was assessed by RNA-Seq. Genes significantly up- or down-regulated by matrix stiffness in WT or Cav1KO MEFs were analyzed. Activation Z-scores are color-coded for each genotype. Positive values (green) indicate activation after exposure to rigid substrate, whereas negative values (red) indicate pathway inhibition. **(D)** *Top,* Venn diagram showing genes up-regulated in WT and Cav1KO MEFs grown on stiff substrates in the RNA-Seq study. *Bottom,* KEGG-term enrichment between genes up-regulated in stiff conditions in WT MEFs, Cav1KO MEFS, and both genotypes (bottom). **(E)** YAP western blot in nuclear and cytosolic fractions of WT and Cav1KO MEFs grown on stiff substrates. RHO-GDI and Histone H3 were used as cytosolic and nuclear internal controls. **(F)** Western blot of CAV1 in MDA-MB-231 cells transfected with CAV1 or control siRNAs. **(G)** qRT-PCR analysis of *Ctgf*, *Ankrd1*, and *Cav1* mRNA expression in MDA-MB-231 cells transfected with control or CAV1 siRNAs. Data are normalized to the control condition (n=4). **(H)** qRT-PCR analysis of *Ctgf* and *Ankrd1* mRNA expression in WT and Cav1KO mouse neonatal hepatocytes. Data are normalized to values in WT cells for each experiment. n = 4. **(I)** Relative distributions of YAP target genes showing positive, negative, or zero expression correlation with *Cav1* across 300 cell lines (p-value > 0.05). **(J and K)** Western blot **(J)** and qRT-PCR **(K)** analysis of YAP targets (*Ctgf*, *Ankrd1*, and *Cyr61*) and *Cav1* expression in cells transfected with control or YAP/TAZ-targeting siRNAs. For qRT-PCR analysis, data are normalized to WT cells grown on stiff substrate. n=3. Data are presented as means ±SEM; *P<0.05, **P<0.01, ***P<0.005.

Figure S2

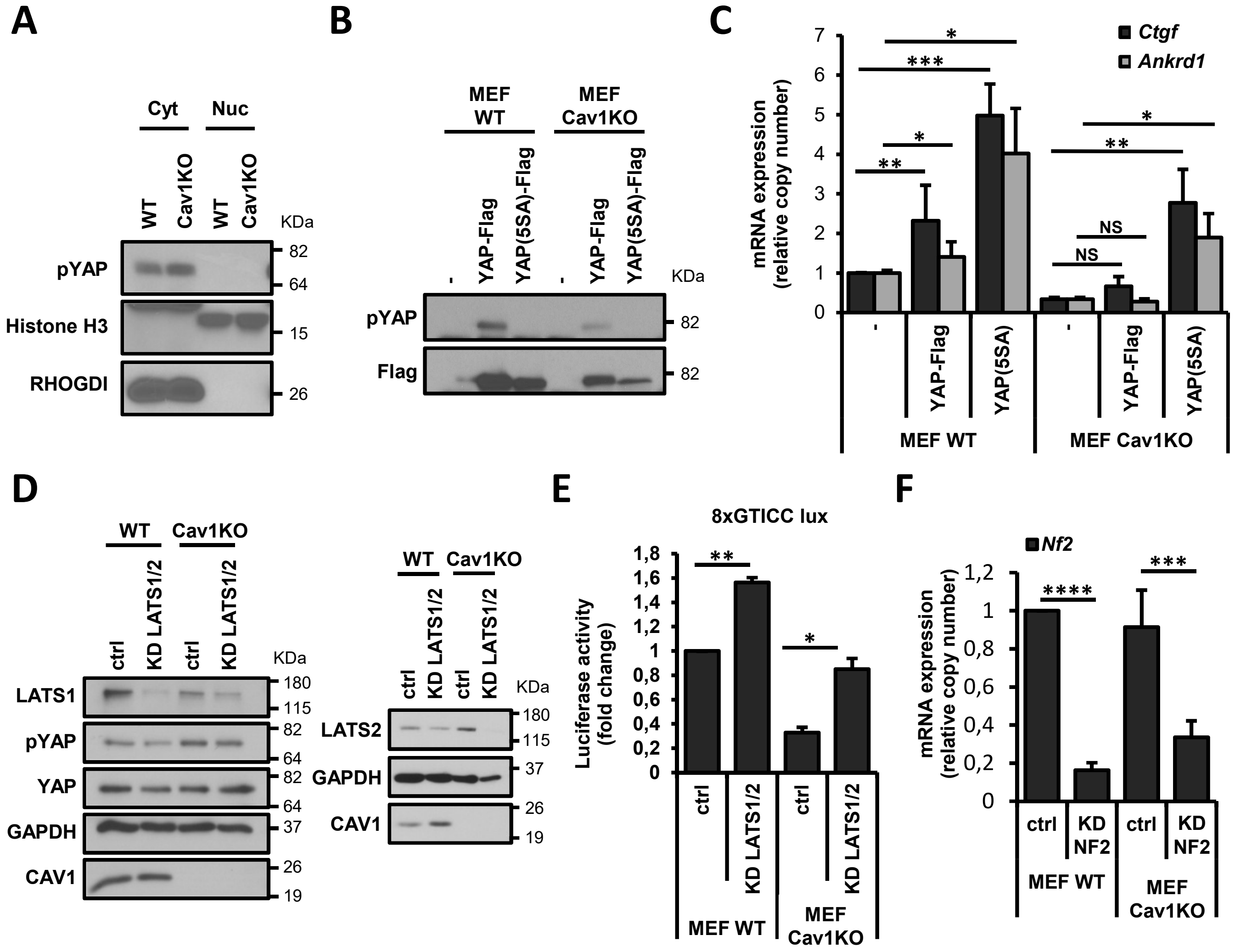

Figure S2. Hippo-kinase-independent YAP phosphorylation determines deficient YAP activity in Cav1KO cells. (Related to Figure 2) (A) Western blot analysis of the subcellular distribution of YAP phosphorylated on Ser112. Nuclear and cytosolic fractions were separated by biochemical fractionation. RHO-GDI and Histone H3 were used as cytosolic and nuclear internal controls. **(B)** Western blot for Flag in WT and Cav1KO MEFS transfected with YAP-Flag or YAP(5SA)-Flag. YAP phosphorylated on Ser112 migrated to the same position as the YAP constructs (which are larger than endogenous YAP) **(C)** qRT-PCR analysis of *Ctgf* and *Ankrd1* expression in cells transfected with YAP-Flag or YAP(5SA)-Flag. Data are normalized to WT control. n=4. **(D)** Western blot for LATS1 and 2, CAV1, S112-phosphorylated YAP, and total YAP in MEFs transfected with control or LATS1/2 siRNAs. GAPDH was used as an internal control. **(E)** TEAD transcriptional activity measured by 8xGTICC-luciferase reporter assay in cells transfected with control or LATS1/2 siRNAs. Data are normalized to WT control. n=3. **(F)** qRT-PCR analysis of *Nf2* expression in WT and Cav1 KO MEFs transfected with control or NF2 siRNAs. Data are normalized to WT control. n=4. Data are presented as means ±SEM. *P<0.05, **P<0.01, ***P<0.005, ****P<0.0005.

Figure S3

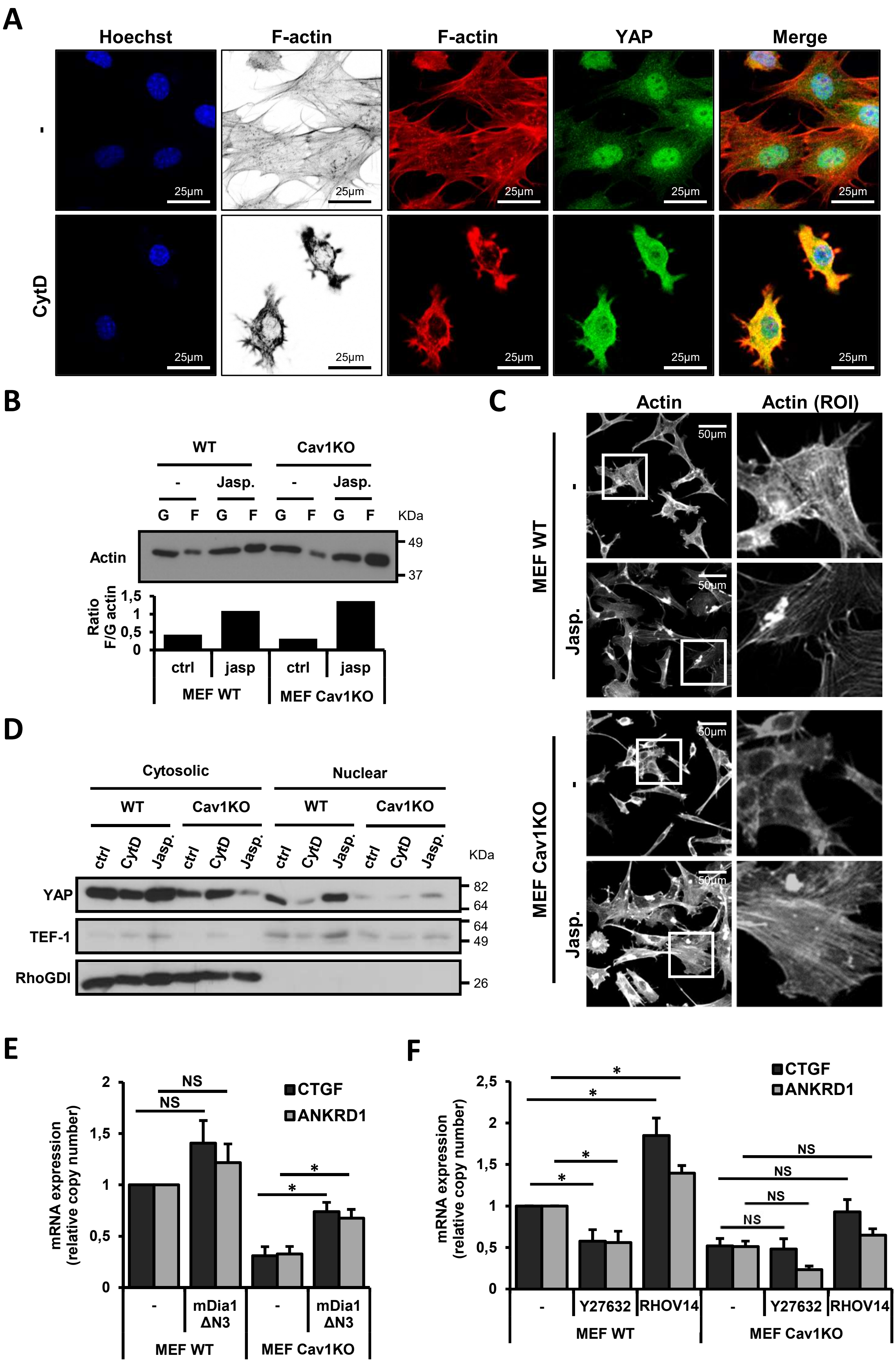

A
Hoechst
F-actin
F-actin
YAP
Merge
-
CytD
25µm
B
WT
Cav1KO
-
Jasp.
G F G F G F G F
KDa
Actin
49
37
Ratio F/G actin
1,5
1
0,5
0
ctrl
jasp
MEF WT
MEF Cav1KO
C
Actin
Actin (ROI)
MEF WT
MEF Cav1KO
Jasp.
50µm
D
Cytosolic
Nuclear
WT
Cav1KO
ctrl
CytD
Jasp.
KDa
YAP
TEF-1
RhoGDI
82
64
49
26
E
mRNA expression (relative copy number)
CTGF
ANKRD1
NS
*
mDia1 ΔN3
MEF WT
MEF Cav1KO
2
1,5
1
0,5
0
F
2,5
Y27632
RHOV14

Figure S3. Altered actin dynamics, but not RhoA function, are a direct cause of the reduced YAP activity in Cav1KO MEFs (Related to Figure 3) (**A**) Conflocal immunofluorencence images of YAP and phalloidin staining in cells treated for 24 hours with CytD (1µM) or DMSO. **(B)** Western blot for filamentous (F) and globular (G) actin in MEFs treated for 24 hours with 0.05µM jasplakinolide (Jasp.) or DMSO. F and G-actin were separated using an in vitro assay kit. Bottom graph showing the ratio between filamentous and globular actin. **(C)** Phalloidin staining in cells treated for 24 hours with 0.05µM jasplakinolide (Jasp.) or DMSO. Zoomed views of the selected ROIs (white squares) are shown on the right. YAP immunostaining is shown in **Figure 3E**. **(D)** YAP subcellular distribution in WT and Cav1KO MEFs treated for 24 hours with 1 µM Cytochalasin D (CytD) or 0.05µM jasplakinolide (jasp). Nuclear and cytosolic fractions were separated by biochemical fractionation. RHO-GDI and TEF-1 were used as cytosolic and nuclear markers, respectively. **(E)** qRT-PCR analysis of *Ctgf* and *Ankrd1* in cells transfected with a constitutively active form of *DIAPH1* (mDia1ΔN3) or mock-transfected. Data are normalized to WT mock-transfected. n =5. **(F)** qRT-PCR analysis of *Ctgf* and *Ankrd1* in cells transfected with a constitutively active RHOA mutant (RHOV14) or treated with the ROCK inhibitor Y27632 for 24 hours. Data are normalized to WT control. n=4. Data in **E** and **F** are presented as means ±SEM. *P<0.05.

Figure S4

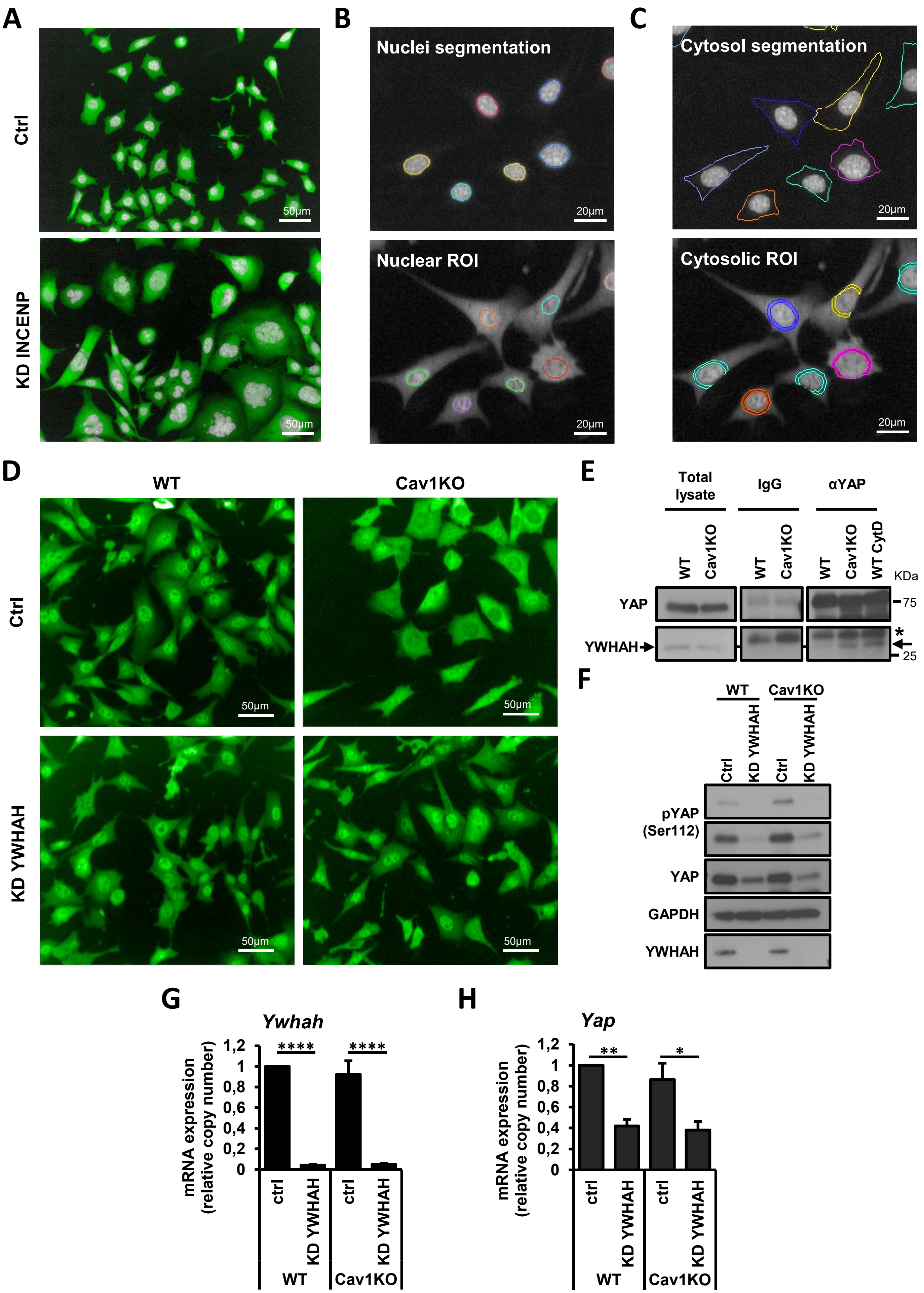
A
Ctrl
KD INCENP
50µm
B
Nuclei segmentation
Nuclear ROI
20µm
C
Cytosol segmentation
Cytosolic ROI
20µm
D
WT
Cav1KO
Ctrl
KD YWHAH
50µm
E
Total lysate
IgG
αYAP
WT
Cav1KO
WT CytD
KDa
YAP
YWHAH
75
25
*
F
WT
Cav1KO
Ctrl
KD YWHAH
pYAP (Ser112)
YAP
GAPDH
YWHAH
G
Ywhah
mRNA expression (relative copy number)
1,2
1
0,8
0,6
0,4
0,2
0
****
ctrl
KD YWHAH
WT
Cav1KO
H
Yap
**
*

Figure S4. Functional analysis of YAP interactors by image-based screening. (Related to Figure 4) (A) Representative images of YAP staining in cells transfected with control or INCENP siRNAs from the image-based screen. **(B)** Example of nuclear segmentation with Columbus^{TM} using Hoechst staining (top) and the ROI used for YAP nuclear intensity calculation based on that segmentation (bottom). **(C)** Cytosolic segmentation with Columbus (top) and the ROI around the nuclei used for YAP cytosolic intensity calculation (bottom). **(D)** Representative images of YAP staining in WT and Cav1KO MEFs transfected with control siRNAs or YWHAH siRNAs identified in the image-based screen. **(E)** Co-immunoprecipitation of YWHAH with anti-YAP in WT and Cav1KO MEFs treated for 24 h with or without 1μM CytD. Negative controls (IgG) were performed in parallel, using a nonspecific IgG instead of anti-YAP antibody. The asterisk marks a nonspecific band. **(F)** Western blot analysis of WT and Cav1KO cells transfected with control or YWHAH siRNAs. **(G and H)** qRT-PCR analysis of *Ywhah* **(G)** and *Yap* **(H)** in WT and Cav1KO MEFs transfected with control or YWHAH siRNAs. n=5. Data are represented as means ±SEM. *P<0.05, **P<0.01, ****P<0.0005.

Figure S5

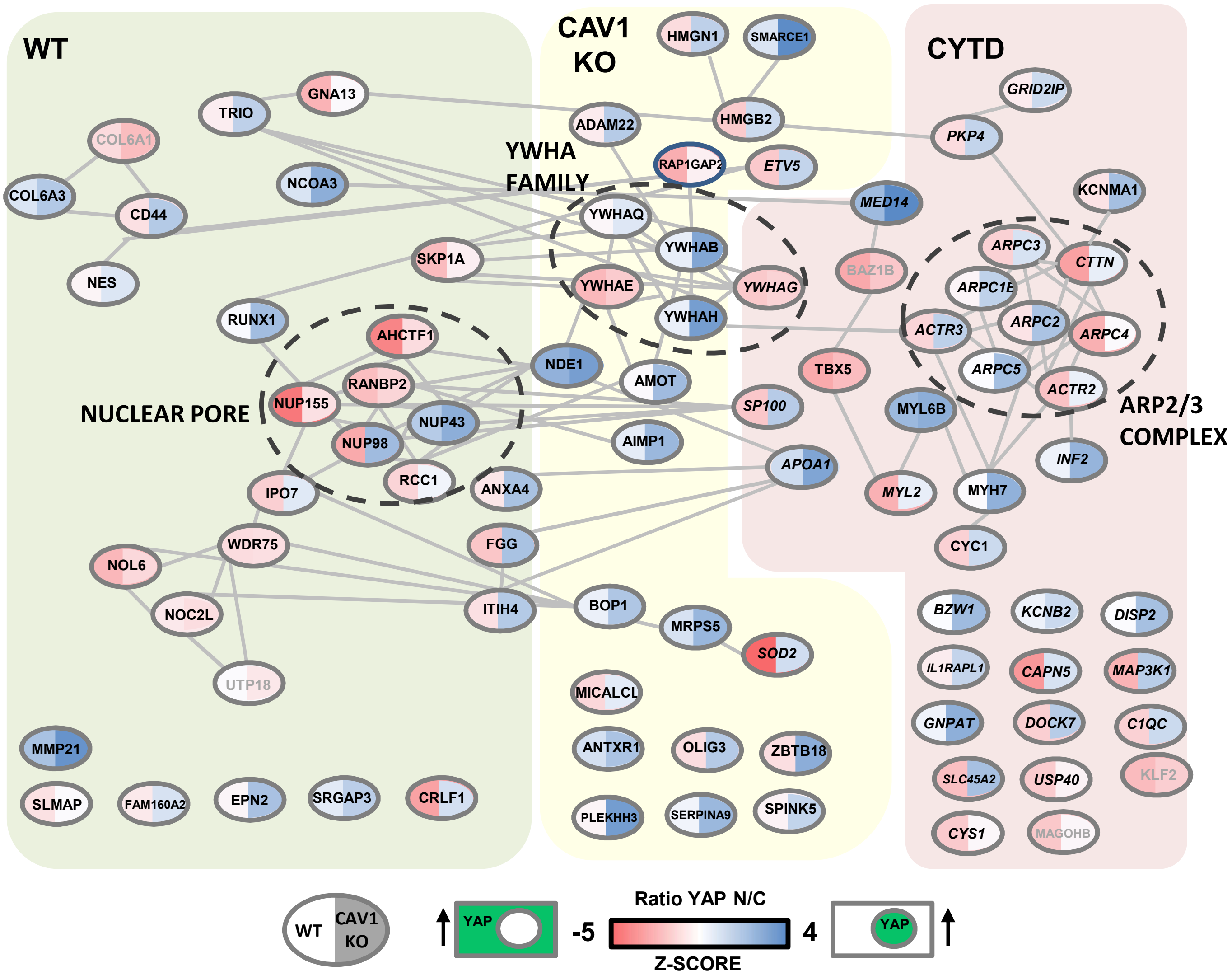

Figure S5. YAP interactome characterization in WT and Cav1KO MEFs. (Related to Figure 4) Functional protein association network based on data from STRING (Szklarczyk et al., 2017), including YAP interactors identified by mass spectrometry in untreated WT MEFs (green region) and Cav1KO MEFs (yellow region) and WT or Cav1KO MEFs treated with CytD (red region). For each gene, the image-based siRNA screen results are indicated as a color code representing the mean Z-score of the YAP nuclear:cytosolic ratio in WT (left side of the node) and Cav1KO cells (right side of the node). n=3.

Figure S6

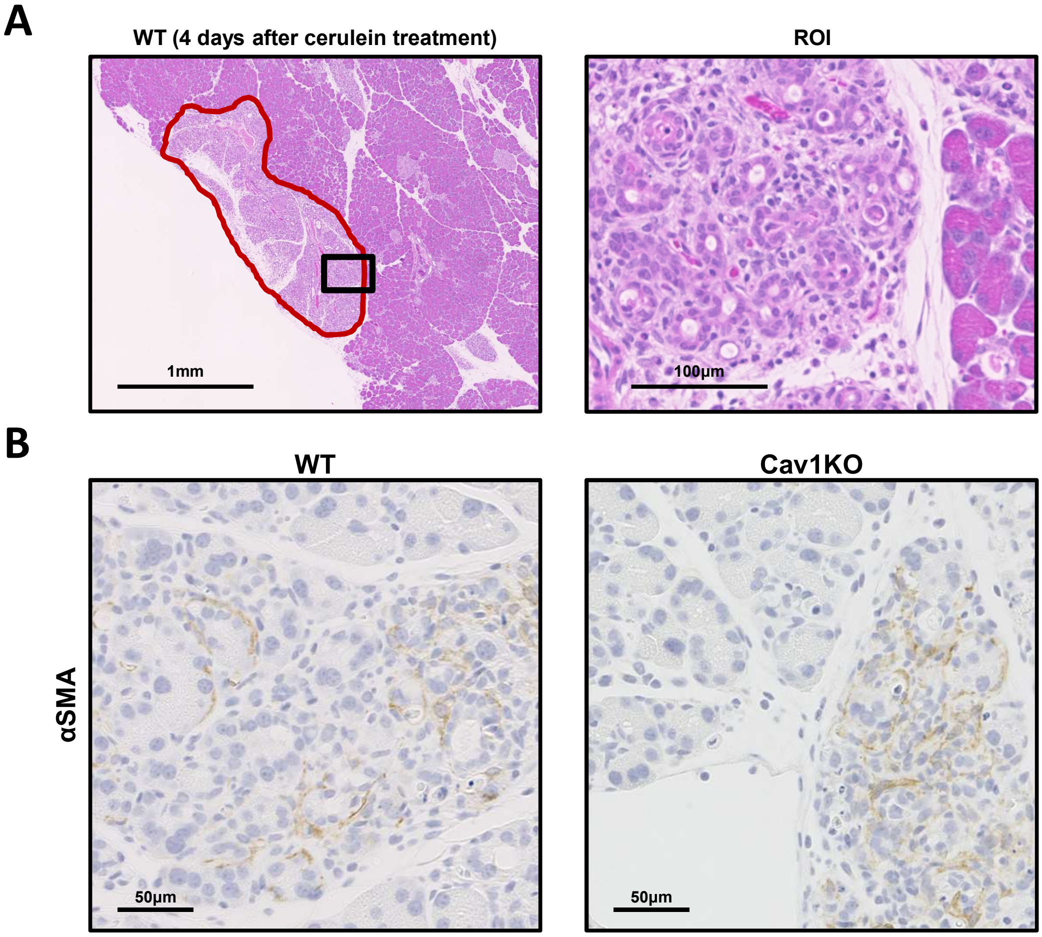

Figure S6. Mouse pancreatic regions presenting extensive ADM and fibrosis after caerulein treatment. (Related to Figure 6) (A) H&E staining. Damaged regions observed 4 days after caerulein administration in WT mice. The red line encloses a region presenting extensive acinar-to-ductal metaplasia (ADM). The right panel shows a zoomed view of the boxed ROI in the left image. **(B)** Immunohistochemistry analysis of αSMA expression in pancreatic tissue of WT and Cav1KO mice 4 days after caerulein treatment.

Table S3. List of siRNAs used. (Related to STAR Methods)

ON-Target Plus Smart-pool siRNAs	
siRNA	IDENTIFIER
ON-Target Plus Smart-pool siRNA targeting YAP	Cat#L-046247-01
ON-Target Plus Smart-pool siRNA targeting TAZ	Cat#L-041057-01
ON-Target Plus Smart-pool siRNA targeting LATS1	Cat#L-063467-01
ON-Target Plus Smart-pool siRNA targeting LATS2	Cat#L-044602-01
ON-Target Plus Smart-pool siRNA targeting Nf2	Cat#L-061566-00
ON-Target Plus Smart-pool siRNA targeting Ywhah	Cat#L-043652-01
ON-Target Plus Smart-pool siRNA targeting Nes	Cat#L-057300-01
ON-Target Plus Smart-pool siRNA targeting Ywhae	Cat#L-062431-01
ON-Target Plus Smart-pool siRNA targeting Baz1b	Cat#L-047645-01
ON-Target Plus Smart-pool siRNA targeting Ywhag	Cat#L-059307-01
ON-Target Plus Smart-pool siRNA targeting Col6a1	Cat#L-043147-01
ON-Target Plus Smart-pool siRNA targeting Noc2l	Cat#L-047536-02
ON-Target Plus Smart-pool siRNA targeting Wdr75	Cat#L-045131-01
ON-Target Plus Smart-pool siRNA targeting Utp18	Cat#L-064167-01
ON-Target Plus Smart-pool siRNA targeting Klf2	Cat#L-046974-01
ON-Target Plus Smart-pool siRNA targeting Nol6	Cat#L-052204-01
ON-Target Plus Smart-pool siRNA targeting Gna13	Cat#L-041077-00
ON-Target Plus Smart-pool siRNA targeting Itih4	Cat#L-046212-01
ON-Target Plus Smart-pool siRNA targeting Slmap	Cat#L-059668-01
ON-Target Plus Smart-pool siRNA targeting Magohb	Cat#L-047702-01
ON-Target Plus Smart-pool siRNA targeting Nup155	Cat#L-059426-01
ON-Target Plus Smart-pool siRNA targeting C1qc	Cat#L-043163-01
ON-Target Plus Smart-pool siRNA targeting Ranbp2	Cat#L-042297-01
ON-Target Plus Smart-pool siRNA targeting Bop1	Cat#L-058593-01
ON-Target Plus Smart-pool siRNA targeting Ahctf1	Cat#L-051465-02
ON-Target Plus Smart-pool siRNA targeting Fam160a2	Cat#L-042848-01
ON-Target Plus Smart-pool siRNA targeting Skp1a	Cat#L-060546-00
ON-Target Plus Smart-pool siRNA targeting Ipo7	Cat#L-058357-01
ON-Target Plus Smart-pool siRNA targeting Rcc1	Cat#L-051808-01
ON-Target Plus Smart-pool siRNA targeting Nup98	Cat#L-060137-01
ON-Target Plus Smart-pool siRNA targeting Fgg	Cat#L-051775-01
ON-Target Plus Smart-pool siRNA targeting Actr3	Cat#L-046642-01
ON-Target Plus Smart-pool siRNA targeting Ywhaq	Cat#L-048160-01
ON-Target Plus Smart-pool siRNA targeting Arpc1b	Cat#L-047321-01
ON-Target Plus Smart-pool siRNA targeting Actr2	Cat#L-053600-01
ON-Target Plus Smart-pool siRNA targeting Arpc4	Cat#L-044870-01
ON-Target Plus Smart-pool siRNA targeting Ywhah	Cat#L-043652-01
ON-Target Plus Smart-pool siRNA targeting Hmgn1	Cat#L-054969-00
ON-Target Plus Smart-pool siRNA targeting Il1rapl1	Cat#L-066757-01
ON-Target Plus Smart-pool siRNA targeting Nup43	Cat#L-052646-01
ON-Target Plus Smart-pool siRNA targeting Trio	Cat#L-065649-00
ON-Target Plus Smart-pool siRNA targeting Cd44	Cat#L-041132-01
ON-Target Plus Smart-pool siRNA targeting Col6a3	Cat#L-065699-01
ON-Target Plus Smart-pool siRNA targeting Runx1	Cat#L-048982-00
ON-Target Plus Smart-pool siRNA targeting Anxa4	Cat#L-057375-00
ON-Target Plus Smart-pool siRNA targeting Srgap3	Cat#L-058941-01
ON-Target Plus Smart-pool siRNA targeting Crlf1	Cat#L-042918-01
ON-Target Plus Smart-pool siRNA targeting Epn2	Cat#L-043345-01
ON-Target Plus Smart-pool siRNA targeting Mmp21	Cat#L-054472-01
ON-Target Plus Smart-pool siRNA targeting Ncoa3	Cat#L-047722-02
ON-Target Plus Smart-pool siRNA targeting Ywhab	Cat#L-059298-01
ON-Target Plus Smart-pool siRNA targeting Inf2	Cat#L-042811-01
ON-Target Plus Smart-pool siRNA targeting Mrps5	Cat#L-046026-01
ON-Target Plus Smart-pool siRNA targeting Myl6b	Cat#L-054158-01
ON-Target Plus Smart-pool siRNA targeting Disp2	Cat#L-059002-01
ON-Target Plus Smart-pool siRNA targeting Arpc5	Cat#L-041235-01
ON-Target Plus Smart-pool siRNA targeting Cttn	Cat#L-044721-00
ON-Target Plus Smart-pool siRNA targeting Hmgb2	Cat#L-066145-01
ON-Target Plus Smart-pool siRNA targeting Smarce1	Cat#L-051327-01
ON-Target Plus Smart-pool siRNA targeting Aimp1	Cat#L-045099-01
ON-Target Plus Smart-pool siRNA targeting Arpc2	Cat#L-043464-01

ON-Target Plus Smart-pool siRNAs	
siRNA	IDENTIFIER
ON-Target Plus Smart-pool siRNA targeting Bzw1	Cat#L-058621-01
ON-Target Plus Smart-pool siRNA targeting Cyc1	Cat#L-047759-01
ON-Target Plus Smart-pool siRNA targeting Med14	Cat#L-057957-01
ON-Target Plus Smart-pool siRNA targeting Gnpat	Cat#L-040695-01
ON-Target Plus Smart-pool siRNA targeting Kcnb2	Cat#L-043145-00
ON-Target Plus Smart-pool siRNA targeting Zbtb18	Cat#L-043562-01
ON-Target Plus Smart-pool siRNA targeting Dock7	Cat#L-065434-01
ON-Target Plus Smart-pool siRNA targeting Amot	Cat#L-058986-02
ON-Target Plus Smart-pool siRNA targeting Arpc3	Cat#L-040765-01
ON-Target Plus Smart-pool siRNA targeting Myh7	Cat#L-048355-01
ON-Target Plus Smart-pool siRNA targeting Sp100	Cat#L-043934-01
ON-Target Plus Smart-pool siRNA targeting Pkp4	Cat#L-045923-01
ON-Target Plus Smart-pool siRNA targeting Apoa1	Cat#L-042942-01
ON-Target Plus Smart-pool siRNA targeting Nde1	Cat#L-050911-01
ON-Target Plus Smart-pool siRNA targeting Kcnma1	Cat#L-041156-00
ON-Target Plus Smart-pool siRNA targeting Sod2	Cat#L-062893-00
ON-Target Plus Smart-pool siRNA targeting Adam22	Cat#L-054425-01
ON-Target Plus Smart-pool siRNA targeting Grid2ip	Cat#L-053712-01
ON-Target Plus Smart-pool siRNA targeting Myl2	Cat#L-045013-01
ON-Target Plus Smart-pool siRNA targeting Olig3	Cat#L-048661-01
ON-Target Plus Smart-pool siRNA targeting Plekhh3	Cat#L-062851-01
ON-Target Plus Smart-pool siRNA targeting Spink5	Cat#L-055789-01
ON-Target Plus Smart-pool siRNA targeting Antxr1	Cat#L-048413-01
ON-Target Plus Smart-pool siRNA targeting Micalcl	Cat#L-172668-00
ON-Target Plus Smart-pool siRNA targeting Serpina9	Cat#L-041728-01
ON-Target Plus Smart-pool siRNA targeting Tbx5	Cat#L-044182-01
ON-Target Plus Smart-pool siRNA targeting Cys1	Cat#L-053758-01
ON-Target Plus Smart-pool siRNA targeting Rap1gap2	Cat#L-040366-01
ON-Target Plus Smart-pool siRNA targeting Usp40	Cat#L-043598-01
ON-Target Plus Smart-pool siRNA targeting Slc45a2	Cat#L-061072-01
ON-Target Plus Smart-pool siRNA targeting Capn5	Cat#L-042053-01
ON-Target Plus Smart-pool siRNA targeting Etv5	Cat#L-062952-01
ON-Target Plus Smart-pool siRNA targeting Map3k1	Cat#L-041090-00
ON-Target Plus Smart-pool siRNA targeting Incenp	Cat#L-049298-01
ON-Target Plus Smart-pool siRNA Control	Cat#D-001810-01
ON-Target Plus Smart-pool siRNA Control	Cat#D-001810-02

Table S4. List of qRT-PCR primers used. (Related to STAR Methods)

Human Primers		
Gene		Sequence (5' -> 3')
HPRT1	Forward Primer	CCTGGCGTCGTGATTAGTGAT
	Reverse Primer	AGACGTTCAGTCCTGTCCATAA
Beta-actin	Forward Primer	CACCTTCCAGCAGATGTCGA
	Reverse Primer	AGCATTTGCGGTGGACGATGG
CTGF	Forward Primer	ACCGACTGGAAGACACGTTTG
	Reverse Primer	CCAGGTCAGCTTCGCAAGG
ANKRD1	Forward Primer	AGTAGAGGAACTGGTCACTGG
	Reverse Primer	TGTTTCTCGCTTTTCCACTGTT
Mouse primers		
Gene		Sequence (5' -> 3')
Hprt1	Forward Primer	GCTGGTGAAAAGGACCTCT
	Reverse Primer	CACAGGACTAGAACACCTGC
Beta-actin	Forward Primer	AGATGACCCAGATCATGTTTGA
	Reverse Primer	CACAGCCTGGATGGCTACGT
Ctgf	Forward Primer	GGACACCTAAAATCGCCAAGC
	Reverse Primer	ACTTAGCCCTGTATGTCTTCACA
Ankrd1	Forward Primer	GCTGGTAACAGGCAAAAAGAAC
	Reverse Primer	CCTCTCGCAGTTTCTCGCT
Cyr61	Forward Primer	CTGCGCTAAACAACTCAACGA
	Reverse Primer	GCAGATCCCTTTCAGAGCGG
Cav1	Forward Primer	CCGCGACCCCAAGCA
	Reverse Primer	CTGCAATCACATCTTCAAAGTC
