# Supplementary material for: Caveolin-1 Modulates Mechanotransduction Responses to Substrate Stiffness through Actin-Dependent Control of YAP
Source: Cell Rep. 2018 Nov 6;25(6):1622–1635.e6. doi: 10.1016/j.celrep.2018.10.024 (PMC6231326; doi:10.1016/j.celrep.2018.10.024)
Supplement: Document S2. Article plus Supplemental Information [file mmc5.pdf]

Cell Reports

Article

# Caveolin-1 Modulates Mechanotransduction Responses to Substrate Stiffness through Actin-Dependent Control of YAP

## Graphical Abstract

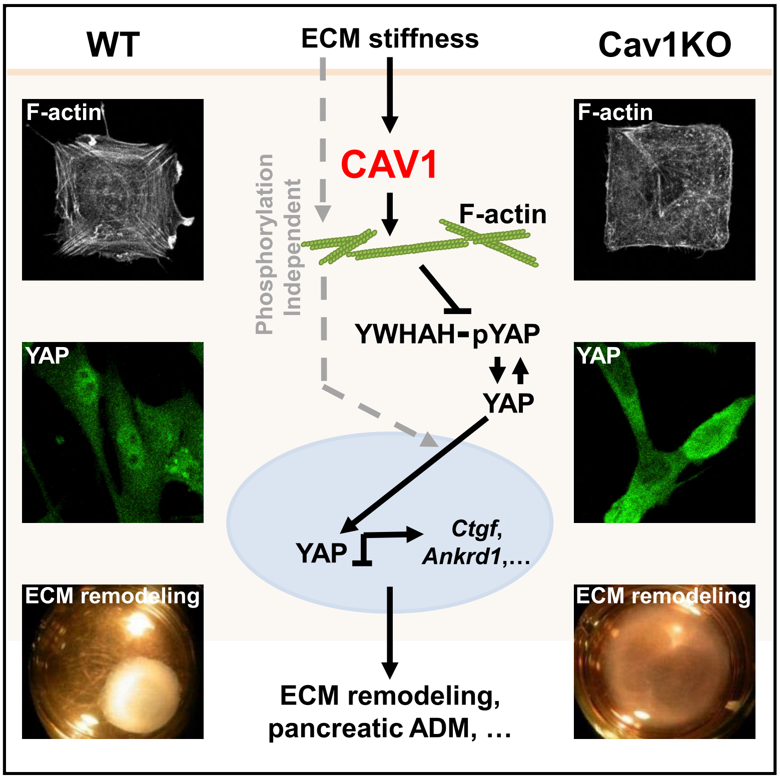


## Authors

Roberto Moreno-Vicente, Dácil María Pavón, Inés Martín-Padura, ..., Jesús Vázquez, Raffaele Strippoli, Miguel A. del Pozo

## Correspondence

madelpozo@cnic.es

## In Brief

Moreno-Vicente et al. report that CAV1, a key component of PM mechanosensing caveolae, mediates adaptation to ECM rigidity by modulating YAP activity through the control of actin dynamics and phosphorylation-dependent interaction of YAP with the 14-3-3-domain protein YWHAH. Cav1-dependent YAP regulation drives two pathophysiological processes: ECM remodeling and pancreatic ADM.

## Highlights

- CAV1 modulates YAP activity in response to changes in ECM stiffness
- CAV1 regulates YAP through the control of actin dynamics and YAP phosphorylation
- YWHAH-YAP interaction blunts YAP activity in CAV1-deficient cells
- CAV1-YAP mechanotransduction drives ECM remodeling and pancreatic ADM

Moreno-Vicente et al., 2018, Cell Reports *25*, 1622–1635
November 6, 2018 © 2018 The Authors.
https://doi.org/10.1016/j.celrep.2018.10.024

OPEN ACCESS
CellPress

Cell Reports
Article

# Caveolin-1 Modulates Mechanotransduction Responses to Substrate Stiffness through Actin-Dependent Control of YAP

Roberto Moreno-Vicente,[1] Dácil María Pavón,[1] Inés Martín-Padura,[1] Mauro Català-Montoro,[1] Alberto Díez-Sánchez,[1] Antonio Quílez-Álvarez,[1] Juan Antonio López,[2] Miguel Sánchez-Álvarez,[1] Jesús Vázquez,[2] Raffaele Strippoli,[3] and Miguel A. del Pozo[1,4,*]

[1]Mechanoadaptation & Caveolae Biology Lab, Cell and Developmental Biology Area, Centro Nacional de Investigaciones Cardiovasculares (CNIC), Madrid 28029, Spain
[2]Cardiovascular Proteomics Unit, CNIC, Madrid 28029, Spain
[3]Section of Molecular Genetics, Department of Cellular Biotechnologies and Hematology, Istituto Pasteur-Fondazione Cenci Bolognetti, Sapienza University of Rome, Rome 00161, Italy
[4]Lead Contact
*Correspondence: madelpozo@cnic.es
https://doi.org/10.1016/j.celrep.2018.10.024

## SUMMARY

**The transcriptional regulator YAP orchestrates many cellular functions, including tissue homeostasis, organ growth control, and tumorigenesis. Mechanical stimuli are a key input to YAP activity, but the mechanisms controlling this regulation remain largely uncharacterized. We show that CAV1 positively modulates the YAP mechanoresponse to substrate stiffness through actin-cytoskeleton-dependent and Hippo-kinase-independent mechanisms. RHO activity is necessary, but not sufficient, for CAV1-dependent mechanoregulation of YAP activity. Systematic quantitative interactomic studies and image-based small interfering RNA (siRNA) screens provide evidence that this actin-dependent regulation is determined by YAP interaction with the 14-3-3 protein YWHAH. Constitutive YAP activation rescued phenotypes associated with CAV1 loss, including defective extracellular matrix (ECM) remodeling. CAV1-mediated control of YAP activity was validated *in vivo* in a model of pancreatitis-driven acinar-to-ductal metaplasia. We propose that this CAV1-YAP mechanotransduction system controls a significant share of cell programs linked to these two pivotal regulators, with potentially broad physiological and pathological implications.**

## INTRODUCTION

The integral membrane protein Caveolin-1 (CAV1) engages in crosstalk with the actin cytoskeleton and connects directly to actin cables through the protein FLNA (Muriel et al., 2011; Stahlhut and van Deurs, 2000). CAV1 controls focal adhesion stability, actin organization, and actomyosin contraction through RHO GTPases (Echarri et al., 2007; Goetz et al., 2011; Grande-García et al., 2007) and contributes to mechanosensing and adaptation in response to various mechanical stimuli, such as membrane stretching, shear stress, hypoosmotic shock, and cell detachment (Boyd et al., 2003; Muriel et al., 2011; Sinha et al., 2011). However, current understanding remains limited regarding the mechanisms by which these phenomena are integrated with overall cell function.

The transcriptional cofactor yes-associated protein (YAP) operates downstream of the canonical Hippo pathway (Piccolo et al., 2014), a highly conserved pathway regulating organ growth control, tissue homeostasis, and tumorigenesis (Yu et al., 2015). YAP regulates the transcription of specific gene sets mainly through its interaction with TEA domain (TEAD) transcription factors (Zhao et al., 2008). A cascade of kinases, including LATS1 and LATS2, lead to YAP phosphorylation and curb its nucleocytoplasmic shuttling, mediating its cytosolic retention through interaction with 14-3-3 proteins, thus downregulating YAP transcriptional output (Dong et al., 2007; Hao et al., 2008; Zhao et al., 2007). This regulatory network is controlled by upstream cues related to tissue architecture and cellular context, such as cell-cell adhesion, cell density, and cell polarity (Piccolo et al., 2014). YAP is also controlled by mechanical signals, such as extracellular matrix (ECM) stiffness, shear stress, and stretching (Codelia et al., 2014; Dupont et al., 2011; Zhong et al., 2013). Stiff environments favor YAP nuclear localization (i.e., activation), whereas attachment to soft substrates increases cytoplasmic retention. This mechanical control, which determines cell proliferation and differentiation (Dupont et al., 2011), depends on RHO GTPase function and actomyosin-driven contractility but is largely independent of kinase regulation, because (1) depletion of LATS1/2 kinases does not alter the mechanical responsiveness of YAP and (2) non-phosphorylatable mutants are nonetheless sensitive to substrate stiffness (Dupont et al., 2011; Elosegui-Artola et al., 2017). The adaptation of nuclear pore units to mechanical tension also contributes to the regulation of YAP nuclear entry (Elosegui-Artola et al., 2017). However, understanding is limited about the exact molecular mechanisms by which ECM stiffness controls YAP activity. Here, we identify CAV1 as an upstream positive regulator of YAP that affects the response

 © 2018 The Authors.
This is an open access article under the CC BY license (http://creativecommons.org/licenses/by/4.0/).

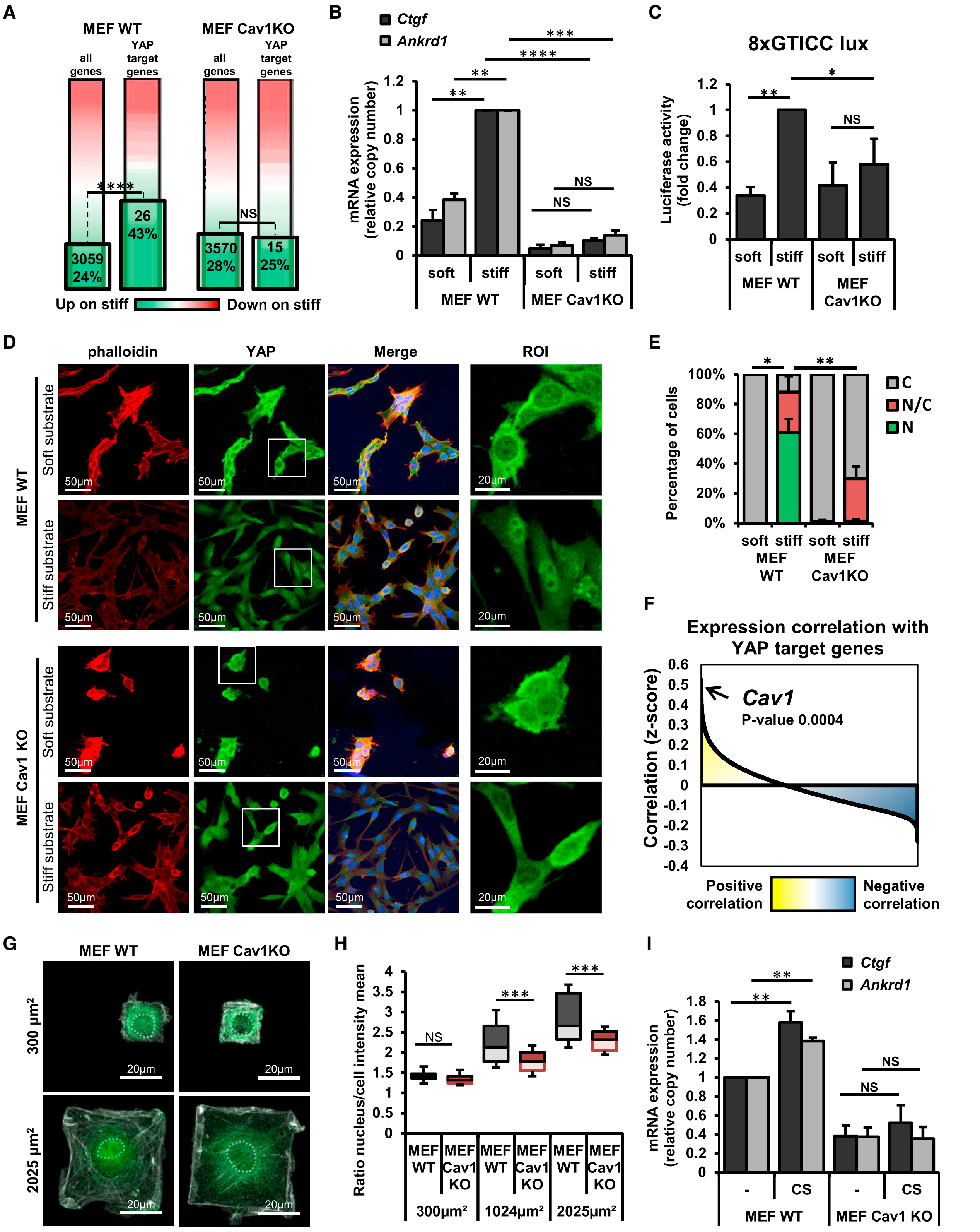


(legend on next page)

to changes in ECM stiffness through a mechanism dependent on F-actin dynamics.

The mechanical regulation of YAP underpins pathophysiological processes such as cardiovascular disease, inflammation and tissue regeneration, and cancer (Panciera et al., 2017). YAP activation by ECM stiffness promotes cancer-associated fibroblast activation and subsequent peritumoral ECM remodeling and stiffening, establishing a positive-feedback loop that favors cancer progression (Calvo et al., 2013). Here, we show that overexpression of constitutively active YAP mutants rescues the blunted contractility and ECM remodeling previously reported for *Cav1* genetic deficiency (Goetz et al., 2011). The positive impact of YAP activity on tumor initiation and progression is further showcased by its critical contribution to pancreatitis-induced acinar-to-ductal metaplasia (ADM), which favors pancreatic ductal carcinoma (PDAC) initiation (Gruber et al., 2016). We further demonstrate CAV1-dependent positive regulation of YAP *in vivo*, showing that Cav1-knockout (Cav1KO) pancreatic parenchyma fails to upregulate YAP in response to induced pancreatitis and exhibits blunting of changes associated with YAP activation, such as ADM.

Our results provide important insight into the mechanisms regulating YAP function. We identify CAV1 as an upstream regulator of YAP, controlling its transcriptional activity through the control of actin cytoskeleton dynamics. Conversely, YAP underpins an important share of CAV1-dependent phenotypes. We propose this CAV1-YAP regulation has important implications in the progression of some pathologies, such as cancer, and will allow us to better understand the principles governing processes driven by substrate stiffness in health and disease.

## RESULTS

### CAV1 Positively Regulates YAP Activity by Controlling YAP Nucleocytoplasmic Shuttling

ECM stiffness mediates CAV1 internalization (Du et al., 2011). We confirmed that CAV1 was internalized in cells grown on soft substrates (Figure S1A) and trafficked to a RAB11-positive recycling endosome (Figure S1B). Thus, cell detachment from integrin-ECM-mediated adhesions and cell growth on soft substrates both trigger the same translocation of CAV1 from the plasma membrane toward a recycling endosome (del Pozo et al., 2005; Muriel et al., 2011). These observations suggest that CAV1 could mediate the response to changes in substrate rigidity. To evaluate the potential contribution of CAV1 to ECM stiffness mechanotransduction, we performed RNA sequencing (RNA-seq) in wild-type (WT) and Cav1KO mouse embryonic fibroblasts (MEFs) cultured on rigid or compliant polyacrylamide hydrogels (GEO: GSE120514). Using Ingenuity Pathway Analysis (IPA) software and the Enrichr open-source tool (Chen et al., 2013; Kuleshov et al., 2016), we queried our datasets for canonical functional programs and Gene Ontology terms responsive to substrate rigidity, classifying them according to their specificity for WT or Cav1KO backgrounds (Figures S1C and S1D). This analysis identified a stiffness-induced increase in genes related to the regulation of actin cytoskeleton, focal adhesions, and cell junctions exclusively in WT cells.

To explore the molecular mechanisms mediating this effect of CAV1 on gene expression, we focused on YAP because this transcriptional cofactor is a prominent transcriptional driver of genes involved in cell adhesion and actin cytoskeleton organization (Stein et al., 2015) and is also positively regulated by mechanical cues such as ECM stiffness (Dupont et al., 2011). To assess whether YAP function was controlled by substrate stiffness in our system, we first analyzed the expression of a panel of 61 genes previously characterized as YAP targets in MCF10A and NIH 3T3 cells (Dupont et al., 2011; Zhao et al., 2008). A Fisher exact test confirmed statistically significant upregulation of endogenous YAP targets by ECM stiffness in WT cells, but not in Cav1KO cells (Figure 1A). This finding was supported by qRT-PCR analysis of the YAP targets *Ankrd1* and *Ctgf* (Figure 1B) and by orthogonal assays to monitor TEAD activity (Figure 1C) based on the 8xGTIIC luciferase reporter (Dupont et al., 2011). To explore the mechanism of this CAV1 dependency, we first studied YAP subcellular distribution (Figure 1D), which was classified as cytosolic (C), nuclear (N), or evenly

**Figure 1. CAV1 Modulates YAP Activity**

(A) Variations in gene expression in the RNA-seq analysis between cells grown on stiff and soft substrates, showing all identified genes (left bar) and YAP-target genes alone (right bar). Genes significantly upregulated by ECM stiffness are boxed, and the enrichment for YAP target genes was analyzed using the Fisher exact test. Genes highlighted green are those that were upregulated on the stiff substrate, whereas those highlighted red were downregulated.

(B) qRT-PCR analysis of *Ctgf* and *Ankrd1* expression in WT and Cav1KO MEFs grown on stiff and soft substrates for 24 hr. Data are normalized to WT cells grown on a stiff substrate. n = 3.

(C) TEAD transcriptional activity in WT and Cav1KO MEFs expressing the 8xGTIIC-luciferase reporter and grown on stiff or soft substrates for 24 hr. Luciferase activity was measured and normalized as described in STAR Methods. Data are normalized to WT MEFs grown on stiff substrate. n = 4.

(D) Confocal immunofluorescence images of YAP expression in WT and Cav1KO MEFs grown on stiff or soft substrate. F-actin was stained with fluorophore-conjugated phalloidin (red; left column), and nuclei were stained with Hoechst (blue in merged images; third column). The right column shows zoomed views of the YAP ROI (boxed in white in the YAP images).

(E) Percentage of cells from analysis as in (D) with predominantly nuclear YAP (N), predominantly cytosolic YAP (C), or an even nuclear-to-cytosolic distribution (N/C). Randomly selected images from 3 independent experiments were analyzed (60–200 interphase cells per condition).

(F) SEEK computational gene co-expression analysis, showing expression correlation (*Z* score) between YAP target genes and the rest of the genome.

(G) Confocal immunofluorescence images of YAP in WT and Cav1KO MEFs plated on different micropatterns with a fibronectin-coated grid that allows cells to spread to a predefined size of 2,025 $\mu m^2$ or 300 $\mu m^2$. Nuclear contours are outlined with dotted gray lines.

(H) ImageJ quantification of YAP subcellular distribution in cells plated on micropatterns of 3 grid sizes (300 $\mu m^2$, 1,024 $\mu m^2$, and 2,025 $\mu m^2$). Data are presented as the nuclear to total cell staining intensities; 10–20 cells were analyzed from 2 biological replicates per condition. The boxplots show the median, 1$^{st}$ and 3$^{rd}$ quartiles, and 90$^{th}$ and 10$^{th}$ percentiles (whiskers).

(I) qRT-PCR analysis of the YAP targets *Ctgf* and *Ankrd1* in cells subjected to cyclic mechanical stretching (CS; see STAR Methods) and unstretched cells. n = 4.

Data in (B), (C), (E), and (I) are presented as means ± SEM; *p < 0.05, **p < 0.01, ***p < 0.005, and ****p < 0.0005. See also Figure S1 and Table S1.

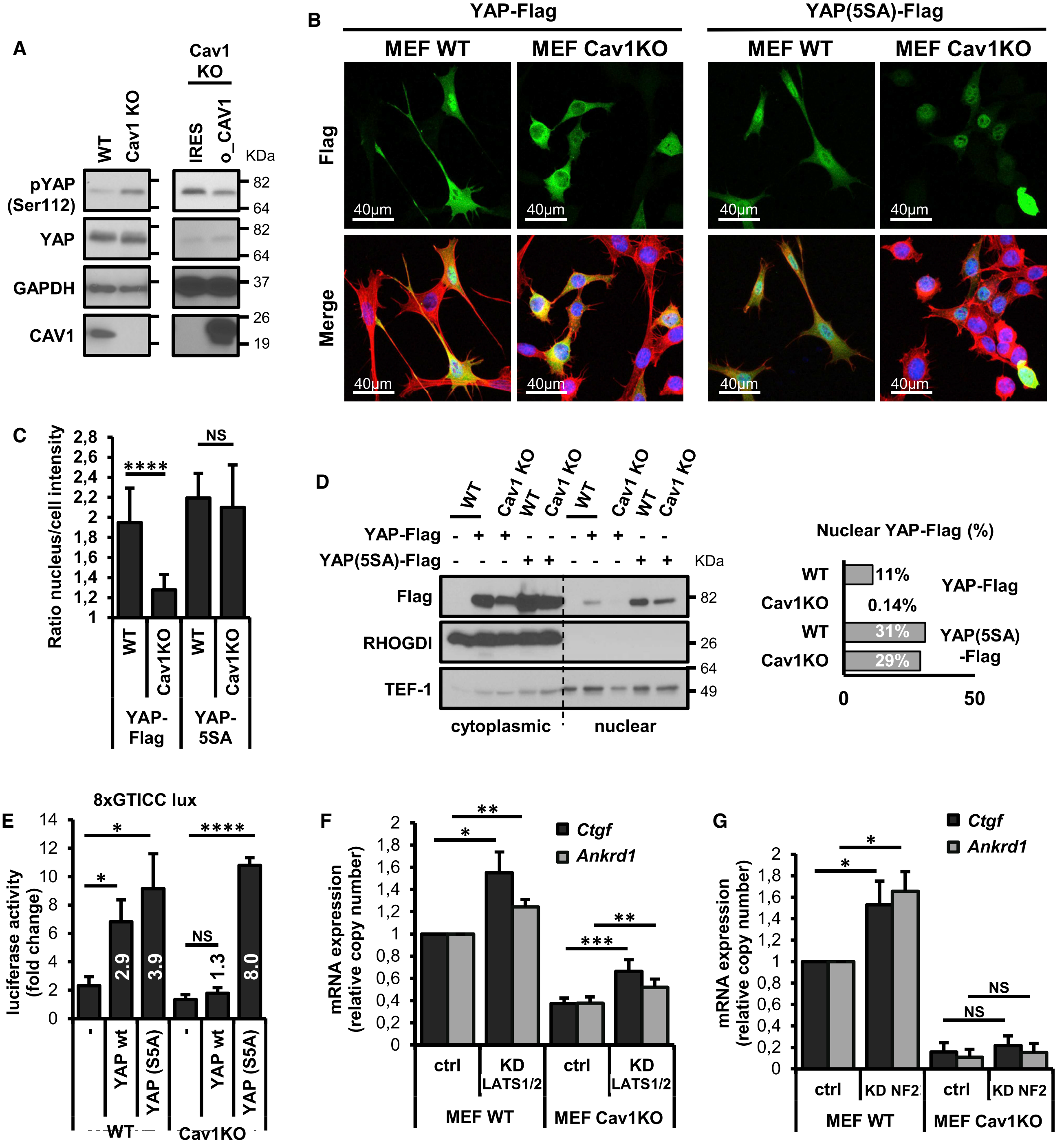


**Figure 2. Hippo-Kinase-Independent YAP Serine Phosphorylation Determines Impaired Nuclear Translocation and Blunted YAP-Dependent Transcriptional Activity in Cav1KO Cells**

(A) Western blot of Ser112-phopshorylated YAP and total YAP in WT and Cav1KO MEFs and Cav1KO MEFs reconstituted with CAV1 (o_CAV1) or IRES-GFP.

(B) Confocal immunofluorescence of cells transfected with YAP-FLAG or YAP(S5A)-FLAG and stained with anti-FLAG antibody (green), fluorophore-conjugated phalloidin (red), and Hoechst (blue).

(C) FLAG distribution from analysis as in (B), represented as the ratio of nuclear-to-cytosolic intensities. n = 6–11.

(D) Western blot analysis of FLAG subcellular distribution in MEFs transfected with YAP-FLAG or YAP(S5A)-FLAG followed by biochemical fractionation. RHO-GDI and Tef-1 were used as cytosolic and nuclear markers, respectively. The percentage of total YAP located in the nuclear fractions was quantified (right graph).

*(legend continued on next page)*

distributed (N/C) (Figure 1E). As expected, YAP was predominantly nuclear in WT cells plated on stiff substrate and retained in the cytosol in cells plated on soft substrate. However, in Cav1KO MEFs, YAP was predominantly retained in the cytoplasm independently of substrate rigidity and compliance. Defective YAP nuclear localization in Cav1KO cells was confirmed by biochemical fractionation (Figure S1E). These results indicate that the positive regulation of YAP transcriptional activity by environmental rigidity is CAV1 dependent.

To rule out a cell-specific effect on YAP-CAV1 functional interactions, we used small interfering RNA (siRNA) duplexes to transiently knock down CAV1 in epithelial MDA-MB-231 human breast carcinoma cells. CAV1 silencing significantly decreased *Ctgf* and *Ankrd1* expression (Figure S1G). Moreover, qRT-PCR profiling of immortalized neonatal mouse hepatocytes revealed a similar reduction in YAP target gene expression in cells harvested from Cav1KO mice compared with those from WT mice (Figure S1H). To further assess the robustness of the CAV1-YAP interaction, we used the SEEK open-access resource (Zhu et al., 2015) to query known YAP target genes for coexpression patterns against the whole genome across extensive datasets from different tissues and cell lines (Figure 1F; Table S1). *Cav1,* whose mRNA levels highly correlate with its protein expression (Sonntag et al., 2014), showed one of the highest expression correlations with our YAP target list query (0.4994). These observations were upheld by the analysis of an independent dataset, generated by assessing the correlation between the expression of *Cav1* and the rest of the genome across 300 cell lines (Pellinen et al., 2018); in this analysis, 79% of YAP target genes correlated positively with *Cav1* expression and 11% correlated negatively (Figure S1G). Together, these observations suggest that CAV1-dependent regulation of YAP transcriptional activity is a general mechanism operating across different experimental systems.

Simultaneous siRNA-mediated knockdown of YAP and TAZ, to prevent potential compensatory mechanisms, effectively blocked expression of the canonical targets *Ctgf* and *Ankrd1* in WT MEFs (Figures S1J and S1K). Consistent with a pivotal role for CAV1 in the positive regulation of YAP, YAP/TAZ silencing in Cav1KO cells did not further decrease *Ctgf*, *Ankrd1*, and *Cyr61* expression. Notably, CAV1 absence did not alter total YAP protein levels (Figure S1J), suggesting that the relationship between CAV1 and YAP-dependent transcriptional programs relies on CAV1-dependent regulatory mechanisms upstream of YAP and not on the regulation of YAP protein expression.

Cell spreading modulates YAP activity such that YAP is predominantly nuclear in cells spread over large areas and cytosolic in cells with limited spreading (Dupont et al., 2011). Moreover, cell polarization and spreading in MEFs is controlled by CAV1 (Grande-García et al., 2007). To rule out the possibility that CAV1-dependent differences in YAP activity were secondary to differential cell spreading, we cultured MEFs on printed fibronectin micropatterns of fixed area and shape. As expected, YAP was predominantly cytosolic in WT MEFs spreading over small micropatterns, whereas growth on large micropatterns promoted a marked nuclear accumulation. This regulation was blunted in CAV1-deficient cells (Figures 1G and 1H). These observations confirm that CAV1-dependent YAP modulation is not an indirect consequence of changes in cell geometry.

To assess this relationship in the context of other mechanical cues, we evaluated the role of CAV1 in cell stretching, another established YAP-activating stimulus (Aragona et al., 2013; Codelia et al., 2014). Using a stretching device, we exposed cells to uniaxial cyclic strain. Stretching induced significant increases in *Ctgf* and *Ankrd1* expression in WT MEFs, but not in Cav1KO cells (Figure 1I), suggesting that CAV1 modulates YAP activity in response to different stimuli.

## CAV1-Dependent Regulation of YAP Is Independent of Hippo Kinase

We observed that YAP phosphorylation at S112 was increased in Cav1KO MEFs, and this increase was partly blocked by exogenous CAV1 expression (Figure 2A). Previous reports proposed the existence of nuclear pools of S127-phosphorylated YAP in human cells (Wada et al., 2011), but our biochemical partition assays suggested that the phosphorylated form of the mouse homologous residue S112 is largely excluded from the nucleus in our cellular model (Figure S2A). YAP phosphorylation at serine 127 (S112 in mice) promotes the retention of this transcription factor in the cytosol (Basu et al., 2003; Zhao et al., 2007). We evaluated the involvement of YAP phosphorylation in CAV1-dependent regulation ectopically expressing YAP-FLAG and the non-phosphorylatable mutant YAP-5SA (Figure S2B). We transiently transfected these constructs into WT and Cav1KO MEFs and analyzed their subcellular distribution by both immunofluorescence and subcellular fractionation (Figures 2B–2D). In WT MEFs, FLAG-tagged WT YAP was predominantly nuclear but was mostly retained in the cytosol in Cav1KO MEFs. However, FLAG-tagged YAP-5SA accumulated in the nucleus in both WT and Cav1KO MEFs, suggesting that cytosolic retention of YAP in Cav1KO MEFs is at least partially dependent on its regulated phosphorylation. Constitutive nuclear translocation of YAP-5SA proteins in Cav1KO MEFs correlated with the rescue of its downstream transcriptional output. YAP-5SA nuclear accumulation in Cav1KO MEFs correlated with increased canonical YAP-TEAD transcriptional activity, assessed by 8xGTICC-luciferase reporter assay (Figure 2E). In contrast, whereas WT YAP enhanced TEAD activity in WT MEFs, it did not in Cav1KO MEFs, consistent with the cytosolic sequestration of WT YAP-FLAG and endogenous YAP in Cav1KO MEFs. These results were confirmed by qRT-PCR analysis (Figure S2C). It is important to note that while the fold increase was higher in Cav1KO cells, YAP-5SA overexpression in Cav1KO cells did not reach the levels observed in WT cells, suggesting

(E) TEAD transcriptional activity in MEFs transfected with YAP-FLAG and YAP(S5A)-FLAG, measured by 8xGTICC-luciferase reporter assay. Data are normalized to growth on the soft substrate in each experiment. n = 3 (E). The fold-change with respect to untransfected cells is indicated above the bars.
(F) qRT-PCR for *Ctgf* and *Ankrd1* expression in WT and CAV1 KO MEFs transfected with control or Lats1 and 2 siRNAs. Data are normalized to WT control. n = 10.
(G) qRT-PCR for *Ctgf* and *Ankrd1* expression in WT and CAV1 KO MEFs transfected with control or NF2 siRNAs. Data are normalized to WT control. n = 4.
Data are presented as mean ± SEM; *p < 0.05, **p < 0.01, ***p < 0.005, and ****p < 0.0005. See also Figure S2.

that phosphorylation-independent mechanisms could also be involved. Our observations indicate that YAP serine phosphorylation has an impact on CAV1-dependent control of YAP localization and activity.

YAP serine phosphorylation can be mediated by the kinases LATS1 and LATS2 (Zhao et al., 2007). Knockdown of LATS1/2 increased *Ctgf* and *Ankrd1* mRNA expression and TEAD-driven luciferase reporter activity in both WT and Cav1KO cells, with comparable fold increases (Figures 2G, S2D, and S2E). To further evaluate the implication of Hippo canonical kinases in the differences observed between WT and Cav1KO cells, we analyzed the role of neurofibromin 2 (NF2). NF2 silencing led to an increase in *Ctgf* and *Ankrd1* expression in WT cells, but not in Cav1KO cells (Figures 2H and S2F), supporting the existence of alternative regulation upon suppression of LATS1/2 kinase activity by NF2 knockdown and precluding rescue of YAP activity in Cav1KO cells. Taken together, these results suggest that LATS1/2 kinases are not essential for CAV1-dependent YAP activity regulation.

## CAV1-Dependent Regulation of YAP Activity Is Exerted through the Control of Actin Polymerization

Since F-actin and RHO are necessary for YAP nuclear translocation and transcriptional activity (Dupont et al., 2011), we next checked whether changes in actin cytoskeleton and RHO signaling could explain the altered YAP regulation in Cav1KO MEFs. For the analysis of actin dynamics and architecture, WT and Cav1KO MEFs were cultured on large fibronectin micropatterns to ensure the same spreading area for both genetic backgrounds and thus exclude effects of spreading area on actin dynamics of cell-cell interaction, spreading, and cell shape (Figures 3A and 3B). Actin dynamics and architecture were also analyzed in cells cultured on stiff substrates (Figure 3C). Actin fiber organization was inferred by anisotropy analysis of microscopy images to measure the degree of departure from a homogeneous distribution toward an increasingly discrete intensity distribution (STAR Methods). Confirming CAV1 as a regulator of actin cytoskeleton organization, actin fibers were less organized in Cav1KO cells.

We assessed the potential contribution of actin dynamics to CAV1-dependent YAP regulation by using the actin polymerization inhibitor cytochalasin D (CytD) and jasplakinolide, an enhancer of F-actin actin polymerization (Holzinger, 2009; Prentki et al., 1979). CytD decreased stress fiber density (Figure S3A), reducing YAP nuclear accumulation and YAP target transcription throughput in WT cells to levels akin to those in Cav1KO cells (Figures 3D and S3A; see also Figures S3D and 4F). Conversely, jasplakinolide enhanced actin polymerization (Figures S3B and S3C), restored YAP nuclear translocation in Cav1KO MEFs to WT levels (Figures 3D and 3E; see also Figure S3D), and increased YAP target gene expression in both WT and Cav1KO cells (Figure 4F). A constitutively active DIAPH1 mutant (mDia1ΔN3), capable of boosting actin polymerization rates (Watanabe et al., 1999), significantly upregulated YAP target expression in Cav1KO cells (Figure S3E). These data strongly suggest that actin polymerization is a key component of the YAP regulatory machinery in our system. Altered actin dynamics in Cav1KO cells are the direct cause of the reduced YAP activity observed in this genetic background.

We next explored the contribution of RHO signaling to actin- and CAV1-dependent regulation of YAP using Y27632, a well-established inhibitor of the upstream kinase ROCK1/2. Exposure to Y27632 strongly reduced YAP target gene expression in WT cells, reproducing the effect of CytD (see Figure 3A); in contrast, Y27632 had only modest effects in Cav1KO cells (Figure S3F). Transient transfection with a constitutively active form of RHOA (RHOV14) that rescues RHO activity in Cav1KO MEFs (Goetz et al., 2011) further increased *Ctgf* and *Ankrd1* expression in WT cells but did not enhance YAP target gene expression in Cav1KO cells (Figure S3F). Our observations thus suggest that while RHO signaling is necessary for the CAV1-dependent positive mechanoregulation of YAP activity, it is not sufficient, since defective RHO cannot explain the deficient YAP activity in Cav1KO cells.

## Deficient YAP Activity in Cav1KO Cells Is Mediated by YWHAH-YAP Interaction

To characterize the molecular mechanisms underpinning the effect of CAV1-dependent actin dynamics on YAP activity, we profiled the YAP interactome by YAP immunoaffinity purification and mass spectrometry (MS) of control and CytD-treated WT and Cav1KO cells (Figure S5; Table S2). We identified several previously described YAP-interacting proteins: AMOTL2 (Zhao et al., 2011), POLR2A (Gavva et al., 1997), TBX5 (Rosenbluh et al., 2012), RUNX1 (Levy et al., 2008), 14-3-3 proteins, and known members of the Hippo pathway interactome (RBM15, CORO1C, DBN1, DOCK7, LIMA1, MTCL1, PKP4, RAD21, and SLMAP; Couzens et al., 2013). Interestingly, only WT cells showed interactions between YAP and nuclear pore and/or transport complexes, presumably reflecting effective nuclear translocation (Figure 4A). Conversely, both Cav1KO and CytD-treated cells were enriched for interactions with 14-3-3 proteins, which are reported to retain phosphorylated YAP in the cytosol (Dong et al., 2007) (Figure 4B).

To assess the contribution to YAP regulation of each component of these context-specific YAP interactomes, we carried out an image-based RNAi focused screen by knocking down 89 identified YAP interactors and comparing YAP subcellular distribution in Cav1KO and WT cells (STAR Methods; Figures 4C, 4D, S4A–S4C, and S5). Setting a stringent threshold of $|Zq| > 2.5$, we identified hits specific to WT cells for 10 genes, whose knockdown blunted YAP nuclear translocation (Figures 4D and 4E). These included siRNA pools targeting most nuclear pore components previously shown to selectively interact with YAP in WT cells (NUP155, NUP98, and AHCTF). Conversely, 8 hits were identified as specific to Cav1KO cells, and siRNA-mediated depletion of these genes enhanced YAP nuclear translocation. This second subset included two Cav1KO-specific YAP interactors, the 14-3-3-domain proteins YWHAH and YWHAB (Figures 4D, 4E, and S4D). We confirmed by western blot that YWHAH interacts preferentially with YAP in Cav1KO cells in CytD-treated WT cells compared with control WT cells (Figure S4E). Accordingly, efficient YWHAH siRNA-mediated depletion partially rescued the expression of YAP targets in Cav1KO cells and CytD-treated WT cells (Figures 4F, S4F, and S4G). Notably, this rescue was not effective in cells grown on soft substrates, indicating that additional mechanisms might be involved in this

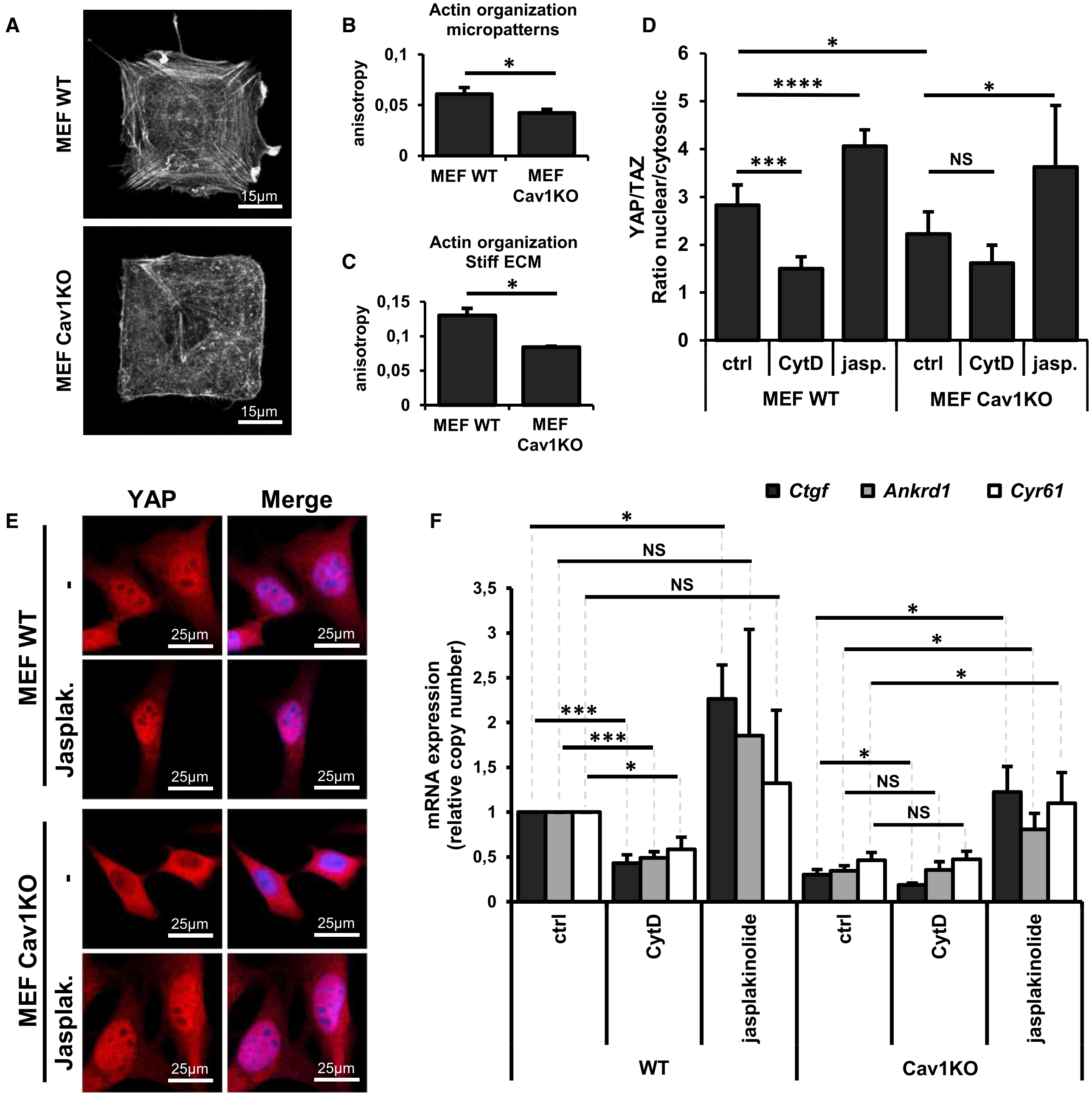


**Figure 3. YAP Activity Defects in Cav1KO MEFs Are a Consequence of Defective Actin Polymerization**

(A) Confocal immunofluorescence images showing actin distribution in cells plated on large squared micropatterns (2,025 $\mu m^2$). Actin was detected by staining with fluorophore-conjugated phalloidin.

(B and C) Quantification of actin-fiber anisotropy (fiber order/organization) in WT and Cav1KO MEFs growing on squared micropatterns (n = 7–10; B) or on a stiff ECM (n = 3; C).

(D) ImageJ quantification of YAP subcellular distribution in cells treated for 24 hr with 1 μM CytD, 0.05 μM jasplakinolide (Jasplak), or DMSO. Data were obtained from 3 to 8 independent experiments and are presented as means ± SD; $^{*}p < 0.05$, $^{***}p < 0.005$, and $^{****}p < 0.0005$.

(E) Confocal immunofluorescence of YAP in cells grown for 24 hr in the presence of 0.05 μM jasplakinolide or DMSO. Nuclei were detected with Hoechst (blue).

(F) qRT-PCR of YAP target genes in MEFs treated for 24 hr with 1 μM CytD, 0.05 μM jasplakinolide, or DMSO (control). Data from jasplakinolide experiments (n = 5) and CytD experiments (n = 3) were normalized to WT controls. Data represent means ± SEM; $^{*}p < 0.05$.

See also Figure S3.

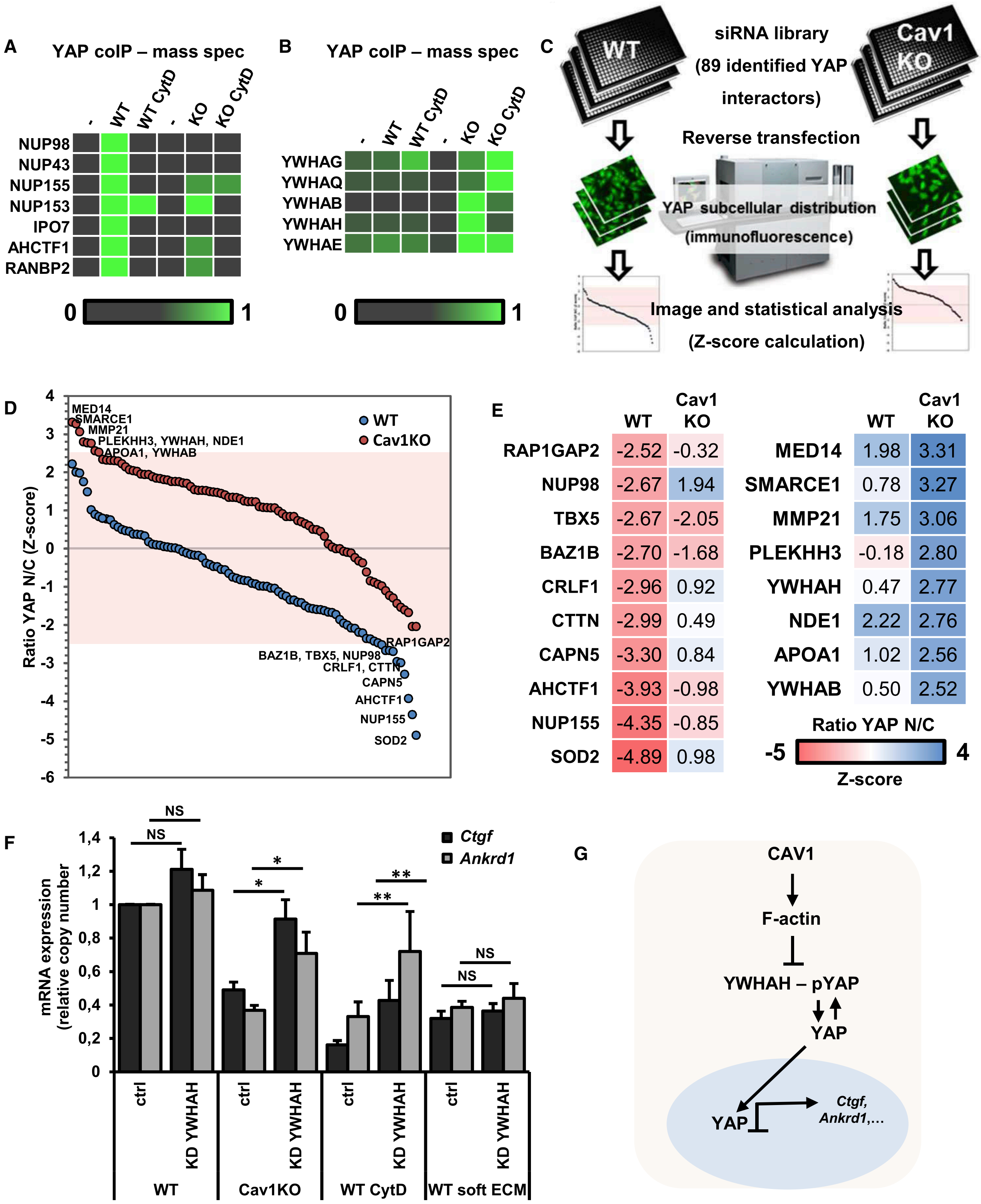


(legend on next page)

regulation. Taken together, these unbiased approaches suggest that CAV1 determines YAP activity through the control of actin dynamics, via mechanisms involving inhibition of the interaction between YAP and 14-3-3 proteins such as YWHAH (Figure 4G).

### YAP Is a Major Effector of ECM Remodeling Downstream of CAV1

YAP and caveolins are involved in a number of pathophysiological processes, such as liver regeneration, muscular dystrophy, and ECM remodeling (Bertrand et al., 2014; Calvo et al., 2013; Fernández et al., 2006; Goetz et al., 2011; Grijalva et al., 2014; Hagiwara et al., 2000; Minetti et al., 1998). We hypothesized that impaired ECM remodeling in CAV1-deficient cells (Goetz et al., 2011) might be caused by deficient YAP activity. To test this, we transfected Cav1KO cells with either non-phosphorylatable YAP-5SA (able to increase YAP transcriptional output in Cav1KO cells; see Figures 3G and 3H) or WT YAP. ECM remodeling was assessed by (1) collagen gel contraction assay and (2) quantitative image analysis of collagen fiber organization by second harmonic generation (SHG) microscopy (Figures 5A and 5B). As expected, ECM remodeling activity was blunted in Cav1KO MEFs. Interestingly, YAP-5SA overexpression restored the ability of these cells to remodel the matrix, whereas WT YAP was ineffective. Furthermore, we observed a clear correlation between CAV1 expression and YAP nuclear localization in human cancer-associated fibroblasts (CAFs) from pancreatic tumors (Figures 5C and 5D), and CAV1 silencing in these cells induced YAP cytosolic retention (Figure 5D), supporting a major role for a CAV1-YAP regulation in determining the activation state of stromal cell populations *in vivo*. Based on these observations, we propose that CAV1 and YAP nucleate a signaling pathway that drives ECM remodeling and stiffening.

### CAV1 Is Required for YAP Activation in Pancreatitis-Associated ADM

Pancreatitis causes tissue damage and desmoplasia, promoting the development of ADM and potentially contributing to PDAC onset and progression (Guerra et al., 2007). We chose pancreatitis as a model to study the potential contribution of CAV1-YAP regulation *in vivo*, because YAP is required for pancreatitis-induced ADM (Morvaridi et al., 2015) and CAV1 expression is upregulated in pancreatic cancer and it is associated with decreased survival (Chatterjee et al., 2015).

Mild and reversible acute pancreatitis was induced in WT and Cav1KO mice by intraperitoneal administration of the cholecistokinin receptor agonist caerulein (Niederau et al., 1985). 2 hr and 4 days after caerulein treatment, nuclear YAP expression was significantly higher in WT preparations (Figures 6A and 6B). This correlates with an increase in the areas presenting extensive ADM and fibrosis, assessed by αSMA expression in pancreatic stellate cells 4 days after treatment (Figures 6C, S6A, and S6B). Taken together, these data suggest that CAV1 is required for YAP activation in the context of caerulein-induced pancreatitis and that this activation correlates with increased ADM in pancreatic tissue.

## DISCUSSION

Our results identify CAV1 as an upstream regulator of YAP-dependent adaptive programs, working through mechanisms dependent on the control of actin dynamics. This CAV1-dependent control of YAP activity relies, at least in part, on the reversible phosphorylation of YAP, evidenced by the association of blunted YAP activity in Cav1KO cells with increased YAP phosphorylation and its rescued by exogenous expression of non-phosphorylatable YAP. We found no role for LATS1/2 in this mechano-dependent negative regulation, observing no YAP activity recovery either upon transient silencing of both kinases or upon silencing of NF2. YAP might also be a substrate for JNK and Abl (Codelia et al., 2014; Levy et al., 2008) or as-yet unidentified kinases that could be responsible for YAP regulation. Another possible explanation for increased YAP phosphorylation in the absence of CAV1 is a protection of phosphorylated YAP from dephosphorylation through interaction with 14-3-3 YWHA proteins. This interpretation is supported by our interactome profiling and systematic functional screening studies, which showed increased interaction of YWHA proteins with YAP in Cav1KO cells and specific rescue of YAP translocation upon their siRNA depletion. YAP retention in the cytosol upon interaction with YWHAH proteins led to deficient YAP transcriptional activity in these cells. Furthermore, YWHAH proteins positively control Yap expression (Figures S4G and 4F), adding a new level of complexity to the control of YAP activity. Our studies also identify several regulators of YAP nuclear translocation, including nuclear pore components and proteins involved in nucleocytosolic transport.

Changes in stromal stiffness and architecture can enhance tumor aggressiveness, promote resistance to therapy, and favor metastasis (Levental et al., 2009). During tumor progression, CAFs surrounding the tumor may favor an increase in the stiffness of the tumor mass. In CAFs, ECM stiffness itself is an activating cue, thus potentially enabling a mechanically driven

**Figure 4. The YAP Interactome Is Altered in Cav1KO MEFs**

(A and B) Mass spectrometry analysis of YWHA proteins (A) and nuclear pore components and nucelocytosolic transporters (B) that co-immunoprecipitate with YAP in WT and Cav1KO MEFs treated with or without 1 μM CytD for 24 hr. The heatmap represents the relative number of counts per protein and condition. Negative controls (first and third column) were performed in parallel by omitting the primary anti-YAP antibody. n = 5.

(C) siRNA library screening scheme for identifying YAP activity regulators.

(D) Plot of mean *Z* scores of the YAP nuclear-to-cytosolic ratio for each individual siRNA in WT and Cav1KO MEFs.

(E) Mean *Z* score of the YAP nuclear-to-cytosolic ratio in WT and Cav1KO cells after siRNA transfection for those genes whose *Z* scores are above 2.5 or below −2.5. n = 3.

(F) qRT-PCR of *Ctgf* and *Ankrd1* in cells transfected with control or YWHAH siRNAs: WT and Cav1KO MEFs, WT cells treated for 24 hr with 1 μM CytD, and WT cells grown on soft substrate. n = 5. Data are presented as means ± SEM. *p < 0.05 and **p < 0.01.

(G) Scheme of CAV1-YAP regulation.

See also Figures S4 and S5, Table S2, and Data S1.

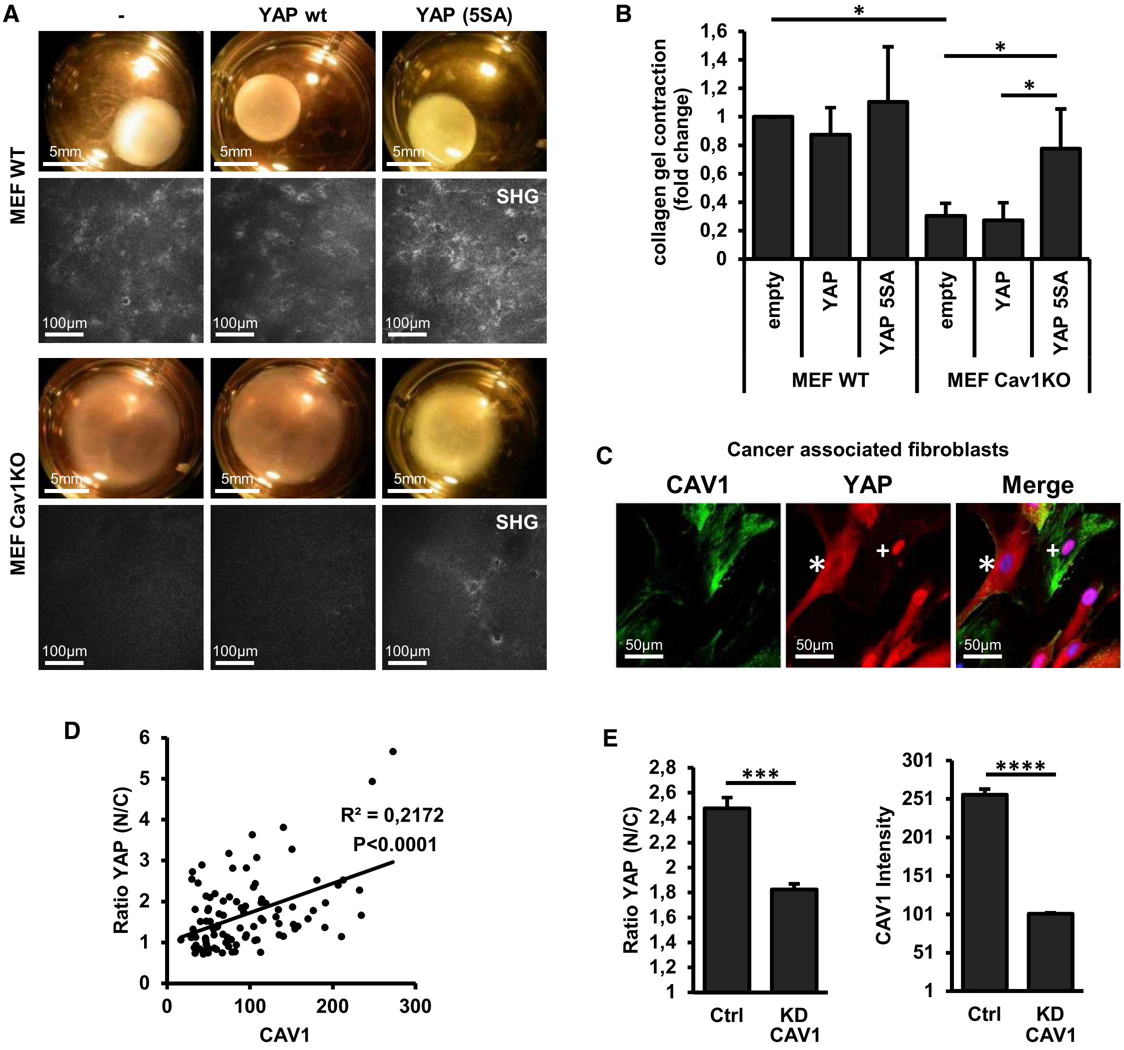


**Figure 5. YAP Mediates the Effect of CAV1 in ECM Remodeling**

(A and B) Collagen gel retraction induced by MEFs transfected with YAP-Flag or YAP(S5A)-FLAG.

(A) Cells were embedded in 3D collagen gels, and images were acquired 72 hr later to monitor gel retraction. Collagen organization was determined by second harmonic generation (SHG) microscopy (black and white images). Mock represents mock transfection.

(B) Corresponding ImageJ quantification of gel contraction, measured as the fold change with respect to the contraction observed in mock-transfected WT cells. n = 3 experiments.

(C) Confocal immunofluorescence images of CAV1 and YAP in cancer-associated fibroblasts (CAFs) extracted from the stroma of human pancreatic tumors and cultured *in vitro*. Nuclei were detected with Hoechst (blue). Symbols mark cells with low (*) or high (+) CAV1 levels.

(D) Plot of YAP nuclear-to-cytosolic ratio against CAV1 intensity for individual CAFs as in (C). N = 97.

(E) Quantification of YAP subcellular distribution (left) and CAV1 levels (right) in CAFs transfected with CAV1 siRNAs or controls. CAV1 and YAP were detected by confocal immunofluorescence microscopy. A total of 4 independent experiments (n = 4) were analyzed (~500 cells per experiment and condition) using Columbus. See STAR Methods for details.

Data are presented as means ± SD in (B) and means ± SEM in (E); *p < 0.05, ***p < 0.005, and ****p < 0.0005.

feedforward loop in which YAP nuclear translocation is necessary for this activation (Calvo et al., 2013). CAV1 expression in CAFs correlates with higher remodeling capacity and facilitates tumor invasion (Goetz et al., 2011). Our results provide the first evidence of a functional connection between these nodes. 3D assays show that exogenous expression of a constitutively active YAP mutant reverts the impairment of ECM remodeling associated with CAV1 deficiency. This proposed CAV1-YAP regulation is therefore likely a significant driver of key events in tumor progression.

Pancreatitis is characterized by immune cell infiltration, interlobular and interacinar edema, and fibrosis and is a major risk factor for the development of pancreatic cancer (Yadav and Lowenfels, 2013). YAP contributes to acinar cell dedifferentiation in ADM and prevents the regeneration of injured areas (Gruber et al., 2016; Murakami et al., 2017). Furthermore, inflammation

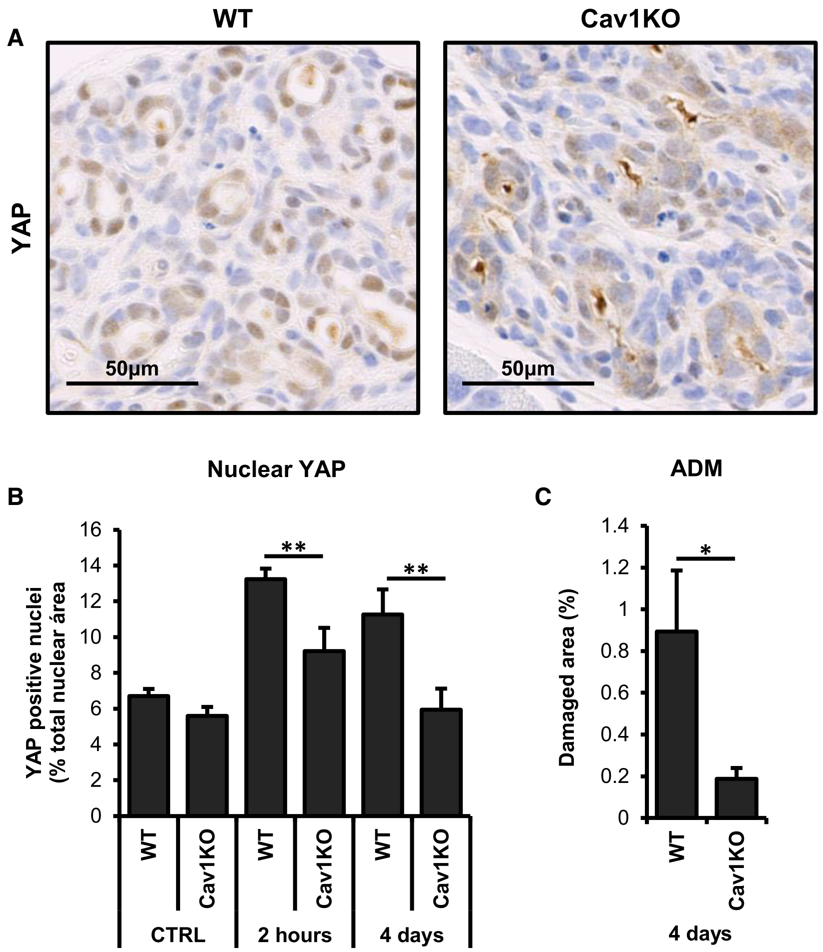


**Figure 6. CAV1 Determines YAP Activation and ADM in Caerulein-Induced Acute Pancreatitis**

(A) Immunohistochemistry analysis of YAP expression in pancreatic tissue of WT and Cav1KO mice 4 days after caerulein treatment.

(B) Quantification of the percentage of the nuclear area covered by YAP staining 2 hr and 4 days after caerulein treatment. n = 8.

(C) Quantification of the percentage pancreatic area showing extensive ADM after 4 days of chronic pancreatitis in WT mice (n = 4) and Cav1KO mice (n = 3). Data in (B) and (C) are presented as means ± SEM; *p < 0.05, **p < 0.01. See also Figure S6.

increases stiffening (Hidalgo, 2012), and the increased tissue stiffness in caerulein-induced acute pancreatitis could explain the differences in YAP activation between WT and Cav1KO mice. Our results thus support an important role for CAV1-YAP regulation *in vivo* and suggest a potential link between inflammation-induced stiffness and disease progression. These results suggest the interesting possibility that CAV1-YAP regulation could determine pancreatic cancer progression, since YAP is required for the initial stages of PDAC development (Gruber et al., 2016).

Our results demonstrate that CAV1 regulates YAP activity, determining the mechanical response to changes in ECM rigidity and other mechanical cues. CAV1-YAP regulation modulates pathophysiological processes such as ECM remodeling and the response to acute pancreatitis. These findings suggest that this regulation could determine the onset and progression of different physiological and pathological processes, such as tumor development, through multiple mechanisms.

## STAR★METHODS

Detailed methods are provided in the online version of this paper and include the following:

- KEY RESOURCES TABLE
- CONTACT FOR REAGENT AND RESOURCE SHARING
- EXPERIMENTAL MODEL AND SUBJECT DETAILS
  - *In vivo* animal studies
  - Cell culture
- METHOD DETAILS
  - Polyacrylamide matrices
  - Micropatterns
  - Cell strain
  - Reagents and transfections
  - Antibodies
  - Immunofluorescence microscopy
  - Subcellular fractionation
  - Immunoprecipitation
  - Immunoblotting
  - Image analysis
  - Second harmonic generation (SHG) imaging
  - Luciferase assay
  - Collagen contraction assay
  - Acute pancreatitis induction
  - Real-time quantitative PCR
  - RNA-Seq analysis
  - Actin fiber organization analysis

- ○ Mass spectrometry analysis
- ○ Image-based siRNA screening
- ○ SEEK analysis
- QUANTIFICATION AND STATISTICAL ANALYSIS
- DATA AND SOFTWARE AVAILABILITY

## SUPPLEMENTAL INFORMATION

Supplemental Information includes six figures, four tables, and one data file and can be found with this article online at https://doi.org/10.1016/j.celrep.2018.10.024.

## ACKNOWLEDGMENTS

This study was supported by the Ministerio de Ciencia, Innovación y Universidades (grants SAF2011-25047, CSD2009-0016, SAF2014-51876-R, and SAF2017-83130-R and IGP-SO grant MINSEV1512-07-2016), the European Regional Development Fund (ERDF "A way to make Europe"), Fundació la Marató de TV3 (grant 674/C/2013), and Worldwide Cancer Research (grant 15-0404) (all to M.A.d.P.). R.M.-V. was supported by Ministerio de Ciencia, Innovación y Universidades (predoctoral fellowship BES-2012-052980; FPI, SAF2011-25047). We thank the CNIC Microscopy, Genomics, and Bioinformatics Units for technical assistance. Daniel Jiménez-Carretero (Cellomics Unit at CNIC) developed image analysis scripts for the quantitative assessment of actin fiber arrangement. We thank Dr. Paloma Martín-Sanz (IIBM Alberto Sols, Madrid, Spain) for neonatal hepatocytes. Simon Bartlett (CNIC) provided English editing. The CNIC is supported by the Ministerio de Ciencia, Innovación y Universidades and the Pro CNIC Foundation and is a Severo Ochoa Center of Excellence (SEV-2015-0505).

## AUTHOR CONTRIBUTIONS

R.M.-V. and M.A.d.P. conceived the study and designed the experiments. R.M.-V., D.M.P., I.M.-P., M.C.-M., A.D.-S., and A.Q.-A. performed the experiments. R.M.-V. and R.S. analyzed the data. J.A.L. and J.V. performed the proteomics analysis. M.S.-A. provided support in image data analysis. R.M-V. and M.A.d.P. wrote the paper with input from M.S.-A., I.M.-P., and R.S.

## DECLARATION OF INTERESTS

The authors declare no competing interests.

Received: March 1, 2018
Revised: August 16, 2018
Accepted: October 3, 2018
Published: November 6, 2018

## REFERENCES

Aragona, M., Panciera, T., Manfrin, A., Giulitti, S., Michielin, F., Elvassore, N., Dupont, S., and Piccolo, S. (2013). A mechanical checkpoint controls multicellular growth through YAP/TAZ regulation by actin-processing factors. Cell *154*, 1047–1059.

Basu, S., Totty, N.F., Irwin, M.S., Sudol, M., and Downward, J. (2003). Akt phosphorylates the Yes-associated protein, YAP, to induce interaction with 14-3-3 and attenuation of p73-mediated apoptosis. Mol. Cell *11*, 11–23.

Bertrand, A.T., Ziaei, S., Ehret, C., Duchemin, H., Mamchaoui, K., Bigot, A., Mayer, M., Quijano-Roy, S., Desguerre, I., Lainé, J., et al. (2014). Cellular microenvironments reveal defective mechanosensing responses and elevated YAP signaling in LMNA-mutated muscle precursors. J. Cell Sci. *127*, 2873–2884.

Bonzon-Kulichenko, E., Garcia-Marques, F., Trevisan-Herraz, M., and Vázquez, J. (2015). Revisiting peptide identification by high-accuracy mass spectrometry: problems associated with the use of narrow mass precursor windows. J. Proteome Res. *14*, 700–710.

Boudaoud, A., Burian, A., Borowska-Wykręt, D., Uyttewaal, M., Wrzalik, R., Kwiatkowska, D., and Hamant, O. (2014). FibrilTool, an ImageJ plug-in to quantify fibrillar structures in raw microscopy images. Nat. Protoc. *9*, 457–463.

Boyd, N.L., Park, H., Yi, H., Boo, Y.C., Sorescu, G.P., Sykes, M., and Jo, H. (2003). Chronic shear induces caveolae formation and alters ERK and Akt responses in endothelial cells. Am. J. Physiol. Heart Circ. Physiol. *285*, H1113–H1122.

Calvo, F., Ege, N., Grande-Garcia, A., Hooper, S., Jenkins, R.P., Chaudhry, S.I., Harrington, K., Williamson, P., Moeendarbary, E., Charras, G., and Sahai, E. (2013). Mechanotransduction and YAP-dependent matrix remodelling is required for the generation and maintenance of cancer-associated fibroblasts. Nat. Cell Biol. *15*, 637–646.

Chatterjee, M., Ben-Josef, E., Thomas, D.G., Morgan, M.A., Zalupski, M.M., Khan, G., Andrew Robinson, C., Griffith, K.A., Chen, C.S., Ludwig, T., et al. (2015). Caveolin-1 is associated with tumor progression and confers a multi-modality resistance phenotype in pancreatic cancer. Sci. Rep. *5*, 10867.

Chen, X., Nadiarynkh, O., Plotnikov, S., and Campagnola, P.J. (2012). Second harmonic generation microscopy for quantitative analysis of collagen fibrillar structure. Nat. Protoc. *7*, 654–669.

Chen, E.Y., Tan, C.M., Kou, Y., Duan, Q., Wang, Z., Meirelles, G.V., Clark, N.R., and Ma'ayan, A. (2013). Enrichr: interactive and collaborative HTML5 gene list enrichment analysis tool. BMC Bioinformatics *14*, 128.

Codelia, V.A., Sun, G., and Irvine, K.D. (2014). Regulation of YAP by mechanical strain through Jnk and Hippo signaling. Curr. Biol. *24*, 2012–2017.

Couzens, A.L., Knight, J.D., Kean, M.J., Teo, G., Weiss, A., Dunham, W.H., Lin, Z.Y., Bagshaw, R.D., Sicheri, F., Pawson, T., et al. (2013). Protein interaction network of the mammalian Hippo pathway reveals mechanisms of kinase-phosphatase interactions. Sci. Signal. *6*, rs15.

del Pozo, M.A., Vicente-Manzanares, M., Tejedor, R., Serrador, J.M., and Sánchez-Madrid, F. (1999). Rho GTPases control migration and polarization of adhesion molecules and cytoskeletal ERM components in T lymphocytes. Eur. J. Immunol. *29*, 3609–3620.

del Pozo, M.A., Balasubramanian, N., Alderson, N.B., Kiosses, W.B., Grande-García, A., Anderson, R.G., and Schwartz, M.A. (2005). Phospho-caveolin-1 mediates integrin-regulated membrane domain internalization. Nat. Cell Biol. *7*, 901–908.

Dong, J., Feldmann, G., Huang, J., Wu, S., Zhang, N., Comerford, S.A., Gayyed, M.F., Anders, R.A., Maitra, A., and Pan, D. (2007). Elucidation of a universal size-control mechanism in Drosophila and mammals. Cell *130*, 1120–1133.

Drab, M., Verkade, P., Elger, M., Kasper, M., Lohn, M., Lauterbach, B., Menne, J., Lindschau, C., Mende, F., Luft, F.C., et al. (2001). Loss of caveolae, vascular dysfunction, and pulmonary defects in caveolin-1 gene-disrupted mice. Science *293*, 2449–2452.

Du, J., Chen, X., Liang, X., Zhang, G., Xu, J., He, L., Zhan, Q., Feng, X.Q., Chien, S., and Yang, C. (2011). Integrin activation and internalization on soft ECM as a mechanism of induction of stem cell differentiation by ECM elasticity. Proc. Natl. Acad. Sci. USA *108*, 9466–9471.

Dupont, S., Morsut, L., Aragona, M., Enzo, E., Giulitti, S., Cordenonsi, M., Zanconato, F., Le Digabel, J., Forcato, M., Bicciato, S., et al. (2011). Role of YAP/TAZ in mechanotransduction. Nature *474*, 179–183.

Echarri, A., Muriel, O., and Del Pozo, M.A. (2007). Intracellular trafficking of raft/caveolae domains: insights from integrin signaling. Semin. Cell Dev. Biol. *18*, 627–637.

Elosegui-Artola, A., Andreu, I., Beedle, A.E.M., Lezamiz, A., Uroz, M., Kosmalska, A.J., Oria, R., Kechagia, J.Z., Rico-Lastres, P., Le Roux, A.L., et al. (2017). Force triggers YAP nuclear entry by regulating transport across nuclear pores. Cell *171*, 1397–1410.e1314.

Fernández, M.A., Albor, C., Ingelmo-Torres, M., Nixon, S.J., Ferguson, C., Kurzchalia, T., Tebar, F., Enrich, C., Parton, R.G., and Pol, A. (2006). Caveolin-1 is essential for liver regeneration. Science *313*, 1628–1632.

Fischer, R.S., Myers, K.A., Gardel, M.L., and Waterman, C.M. (2012). Stiffness-controlled three-dimensional extracellular matrices for high-resolution imaging of cell behavior. Nat. Protoc. *7*, 2056–2066.

Gavva, N.R., Gavva, R., Ermekova, K., Sudol, M., and Shen, C.J. (1997). Interaction of WW domains with hematopoietic transcription factor p45/NF-E2 and RNA polymerase II. J. Biol. Chem. *272*, 24105–24108.

Goetz, J.G., Minguet, S., Navarro-Lérida, I., Lazcano, J.J., Samaniego, R., Calvo, E., Tello, M., Osteso-Ibáñez, T., Pellinen, T., Echarri, A., et al. (2011). Biomechanical remodeling of the microenvironment by stromal caveolin-1 favors tumor invasion and metastasis. Cell *146*, 148–163.

Grande-García, A., Echarri, A., de Rooij, J., Alderson, N.B., Waterman-Storer, C.M., Valdivielso, J.M., and del Pozo, M.A. (2007). Caveolin-1 regulates cell polarization and directional migration through Src kinase and Rho GTPases. J. Cell Biol. *177*, 683–694.

Grijalva, J.L., Huizenga, M., Mueller, K., Rodriguez, S., Brazzo, J., Camargo, F., Sadri-Vakili, G., and Vakili, K. (2014). Dynamic alterations in Hippo signaling pathway and YAP activation during liver regeneration. Am. J. Physiol. Gastrointest. Liver Physiol. *307*, G196–G204.

Gruber, R., Panayiotou, R., Nye, E., Spencer-Dene, B., Stamp, G., and Behrens, A. (2016). YAP1 and TAZ control pancreatic cancer initiation in mice by direct up-regulation of JAK-STAT3 signaling. Gastroenterology *151*, 526–539.

Guerra, C., Schuhmacher, A.J., Cañamero, M., Grippo, P.J., Verdaguer, L., Pérez-Gallego, L., Dubus, P., Sandgren, E.P., and Barbacid, M. (2007). Chronic pancreatitis is essential for induction of pancreatic ductal adenocarcinoma by K-Ras oncogenes in adult mice. Cancer Cell *11*, 291–302.

Hagiwara, Y., Sasaoka, T., Araishi, K., Imamura, M., Yorifuji, H., Nonaka, I., Ozawa, E., and Kikuchi, T. (2000). Caveolin-3 deficiency causes muscle degeneration in mice. Hum. Mol. Genet. *9*, 3047–3054.

Hao, Y., Chun, A., Cheung, K., Rashidi, B., and Yang, X. (2008). Tumor suppressor LATS1 is a negative regulator of oncogene YAP. J. Biol. Chem. *283*, 5496–5509.

Hellemans, J., Mortier, G., De Paepe, A., Speleman, F., and Vandesompele, J. (2007). qBase relative quantification framework and software for management and automated analysis of real-time quantitative PCR data. Genome Biol. *8*, R19.

Hidalgo, M. (2012). New insights into pancreatic cancer biology. Ann. Oncol. *23* (*Suppl 10*), x135–x138.

Holzinger, A. (2009). Jasplakinolide: an actin-specific reagent that promotes actin polymerization. Methods Mol. Biol. *586*, 71–87.

Ishizaki, T., Morishima, Y., Okamoto, M., Furuyashiki, T., Kato, T., and Narumiya, S. (2001). Coordination of microtubules and the actin cytoskeleton by the Rho effector mDia1. Nat. Cell Biol. *3*, 8–14.

Kuleshov, M.V., Jones, M.R., Rouillard, A.D., Fernandez, N.F., Duan, Q., Wang, Z., Koplev, S., Jenkins, S.L., Jagodnik, K.M., Lachmann, A., et al. (2016). Enrichr: a comprehensive gene set enrichment analysis web server 2016 update. Nucleic Acids Res. *44* (*W1*), W90–W97.

Levental, K.R., Yu, H., Kass, L., Lakins, J.N., Egeblad, M., Erler, J.T., Fong, S.F., Csiszar, K., Giaccia, A., Weninger, W., et al. (2009). Matrix crosslinking forces tumor progression by enhancing integrin signaling. Cell *139*, 891–906.

Levy, D., Adamovich, Y., Reuven, N., and Shaul, Y. (2008). Yap1 phosphorylation by c-Abl is a critical step in selective activation of proapoptotic genes in response to DNA damage. Mol. Cell *29*, 350–361.

Mahoney, W.M., Jr., Hong, J.H., Yaffe, M.B., and Farrance, I.K. (2005). The transcriptional co-activator TAZ interacts differentially with transcriptional enhancer factor-1 (TEF-1) family members. Biochem. J. *388*, 217–225.

Martínez-Bartolomé, S., Navarro, P., Martín-Maroto, F., López-Ferrer, D., Ramos-Fernández, A., Villar, M., García-Ruiz, J.P., and Vázquez, J. (2008). Properties of average score distributions of SEQUEST: the probability ratio method. Mol. Cell. Proteomics *7*, 1135–1145.

Mayoral, R., Valverde, A.M., Llorente Izquierdo, C., González-Rodríguez, A., Boscá, L., and Martín-Sanz, P. (2010). Impairment of transforming growth factor beta signaling in caveolin-1-deficient hepatocytes: role in liver regeneration. J. Biol. Chem. *285*, 3633–3642.

Minetti, C., Sotgia, F., Bruno, C., Scartezzini, P., Broda, P., Bado, M., Masetti, E., Mazzocco, M., Egeo, A., Donati, M.A., et al. (1998). Mutations in the caveolin-3 gene cause autosomal dominant limb-girdle muscular dystrophy. Nat. Genet. *18*, 365–368.

Morvaridi, S., Dhall, D., Greene, M.I., Pandol, S.J., and Wang, Q. (2015). Role of YAP and TAZ in pancreatic ductal adenocarcinoma and in stellate cells associated with cancer and chronic pancreatitis. Sci. Rep. *5*, 16759.

Murakami, S., Shahbazian, D., Surana, R., Zhang, W., Chen, H., Graham, G.T., White, S.M., Weiner, L.M., and Yi, C. (2017). Yes-associated protein mediates immune reprogramming in pancreatic ductal adenocarcinoma. Oncogene *36*, 1232–1244.

Muriel, O., Echarri, A., Hellriegel, C., Pavón, D.M., Beccari, L., and Del Pozo, M.A. (2011). Phosphorylated filamin A regulates actin-linked caveolae dynamics. J. Cell Sci. *124*, 2763–2776.

Navarro, P., and Vázquez, J. (2009). A refined method to calculate false discovery rates for peptide identification using decoy databases. J. Proteome Res. *8*, 1792–1796.

Navarro-Lérida, I., Pellinen, T., Sanchez, S.A., Guadamillas, M.C., Wang, Y., Mirtti, T., Calvo, E., and Del Pozo, M.A. (2015). Rac1 nucleocytoplasmic shuttling drives nuclear shape changes and tumor invasion. Dev. Cell *32*, 318–334.

Niederau, C., Ferrell, L.D., and Grendell, J.H. (1985). Caerulein-induced acute necrotizing pancreatitis in mice: protective effects of proglumide, benzotript, and secretin. Gastroenterology *88*, 1192–1204.

Oka, T., Mazack, V., and Sudol, M. (2008). Mst2 and Lats kinases regulate apoptotic function of Yes kinase-associated protein (YAP). J. Biol. Chem. *283*, 27534–27546.

Orimo, A., Gupta, P.B., Sgroi, D.C., Arenzana-Seisdedos, F., Delaunay, T., Naeem, R., Carey, V.J., Richardson, A.L., and Weinberg, R.A. (2005). Stromal fibroblasts present in invasive human breast carcinomas promote tumor growth and angiogenesis through elevated SDF-1/CXCL12 secretion. Cell *121*, 335–348.

Panciera, T., Azzolin, L., Cordenonsi, M., and Piccolo, S. (2017). Mechanobiology of YAP and TAZ in physiology and disease. Nat. Rev. Mol. Cell Biol. *18*, 758–770.

Pellinen, T., Blom, S., Sánchez, S., Välimäki, K., Mpindi, J.P., Azegrouz, H., Strippoli, R., Nieto, R., Vitón, M., Palacios, I., et al. (2018). ITGB1-dependent upregulation of Caveolin-1 switches TGF$\beta$ signalling from tumour-suppressive to oncogenic in prostate cancer. Sci. Rep. *8*, 2338.

Piccolo, S., Dupont, S., and Cordenonsi, M. (2014). The biology of YAP/TAZ: hippo signaling and beyond. Physiol. Rev. *94*, 1287–1312.

Prentki, M., Chaponnier, C., Jeanrenaud, B., and Gabbiani, G. (1979). Actin microfilaments, cell shape, and secretory processes in isolated rat hepatocytes. Effect of phalloidin and cytochalasin D. J. Cell Biol. *81*, 592–607.

Razani, B., Engelman, J.A., Wang, X.B., Schubert, W., Zhang, X.L., Marks, C.B., Macaluso, F., Russell, R.G., Li, M., Pestell, R.G., et al. (2001). Caveolin-1 null mice are viable but show evidence of hyperproliferative and vascular abnormalities. J. Biol. Chem. *276*, 38121–38138.

Rosenbluh, J., Nijhawan, D., Cox, A.G., Li, X., Neal, J.T., Schafer, E.J., Zack, T.I., Wang, X., Tsherniak, A., Schinzel, A.C., et al. (2012). $\beta$-Catenin-driven cancers require a YAP1 transcriptional complex for survival and tumorigenesis. Cell *151*, 1457–1473.

Sinha, B., Köster, D., Ruez, R., Gonnord, P., Bastiani, M., Abankwa, D., Stan, R.V., Butler-Browne, G., Vedie, B., Johannes, L., et al. (2011). Cells respond to mechanical stress by rapid disassembly of caveolae. Cell *144*, 402–413.

Sonntag, J., Bender, C., Soons, Z., der Heyde, S.v., König, R., Wiemann, S., Sinn, H.-P., Schneeweiss, A., Beißbarth, T., and Korf, U. (2014). Reverse phase protein array based tumor profiling identifies a biomarker signature for risk classification of hormone receptor-positive breast cancer. Transl. Proteom. *2*, 52–59.

Stahlhut, M., and van Deurs, B. (2000). Identification of filamin as a novel ligand for caveolin-1: evidence for the organization of caveolin-1-associated membrane domains by the actin cytoskeleton. Mol. Biol. Cell *11*, 325–337.

Stein, C., Bardet, A.F., Roma, G., Bergling, S., Clay, I., Ruchti, A., Agarinis, C., Schmelzle, T., Bouwmeester, T., Schübeler, D., and Bauer, A. (2015). YAP1 Exerts Its Transcriptional Control via TEAD-Mediated Activation of Enhancers. PLoS Genet. *11*, e1005465.

Tse, J.R., and Engler, A.J. (2010). Preparation of hydrogel substrates with tunable mechanical properties. Curr. Protoc. Cell Biol *Chapter 10*, Unit 10.16.

Wada, K., Itoga, K., Okano, T., Yonemura, S., and Sasaki, H. (2011). Hippo pathway regulation by cell morphology and stress fibers. Development *138*, 3907–3914.

Watanabe, N., Kato, T., Fujita, A., Ishizaki, T., and Narumiya, S. (1999). Cooperation between mDia1 and ROCK in Rho-induced actin reorganization. Nat. Cell Biol. *1*, 136–143.

Yadav, D., and Lowenfels, A.B. (2013). The epidemiology of pancreatitis and pancreatic cancer. Gastroenterology *144*, 1252–1261.

Yu, F.X., Zhao, B., and Guan, K.L. (2015). Hippo pathway in organ size control, tissue homeostasis, and cancer. Cell *163*, 811–828.

Zhao, B., Wei, X., Li, W., Udan, R.S., Yang, Q., Kim, J., Xie, J., Ikenoue, T., Yu, J., Li, L., et al. (2007). Inactivation of YAP oncoprotein by the Hippo pathway is involved in cell contact inhibition and tissue growth control. Genes Dev. *21*, 2747–2761.

Zhao, B., Ye, X., Yu, J., Li, L., Li, W., Li, S., Yu, J., Lin, J.D., Wang, C.Y., Chinnaiyan, A.M., et al. (2008). TEAD mediates YAP-dependent gene induction and growth control. Genes Dev. *22*, 1962–1971.

Zhao, B., Li, L., Lu, Q., Wang, L.H., Liu, C.Y., Lei, Q., and Guan, K.L. (2011). Angiomotin is a novel Hippo pathway component that inhibits YAP oncoprotein. Genes Dev. *25*, 51–63.

Zhong, W., Tian, K., Zheng, X., Li, L., Zhang, W., Wang, S., and Qin, J. (2013). Mesenchymal stem cell and chondrocyte fates in a multishear microdevice are regulated by Yes-associated protein. Stem Cells Dev. *22*, 2083–2093.

Zhu, Q., Wong, A.K., Krishnan, A., Aure, M.R., Tadych, A., Zhang, R., Corney, D.C., Greene, C.S., Bongo, L.A., Kristensen, V.N., et al. (2015). Targeted exploration and analysis of large cross-platform human transcriptomic compendia. Nat. Methods *12*, 211–214, 3, 214.

# STAR★METHODS

## KEY RESOURCES TABLE

| REAGENT or RESOURCE | SOURCE | IDENTIFIER |
|---|---|---|
| Antibodies | | |
| Mouse monoclonal anti-YAP (63.7) | Santa Cruz Biotechnology | Cat# sc-101199, RRID: AB_1131430 |
| Mouse monoclonal anti-TEF-1 (H-4) | Santa Cruz Biotechnology | Cat# sc-376113, RRID: AB_10988229 |
| Rabbit monoclonal anti-CAV1 (D46G3) XP | Cell Signaling Tecnology | Cat# 3267S, RRID: AB_2275453 |
| Rabbit monoclonal anti-14-3-3 η (D23B7) | Cell Signaling Tecnology | Cat# 5521S, RRID: AB_10829034 |
| Monoclonal ANTI-FLAG® M2 antibody | Sigma-Aldrich | Cat# F3165, RRID: AB_259529 |
| Mouse monoclonal anti-glyceraldehyde-3-phosphate dehydrogenase antibody | Millipore | Cat# MAB374, RRID: AB_2107445 |
| Rabbit polyclonal anti-Phospho-YAP (Ser127) Antibody | Cell Signaling Technology | Cat# 4911S, RRID: AB_2218913 |
| Rabbit polyclonal anti-Anti-LATS1 Antibody | Cell Signaling Technology | Cat# 9153S, RRID: AB_2296754 |
| Rabbit polyclonal anti-Anti-LATS1 Antibody | Cell Signaling Technology | Cat#13646 |
| Rabbit polyclonal anti-Anti-H3 Histone Antibody | Abcam | Cat# ab1791, RRID: AB_302613 |
| Rabbit polyclonal anti-RHO GDIalpha (A-20) antibody | Santa Cruz Biotechnology | Cat# sc-360, RRID: AB_2227516 |
| Rabbit monoclonal anti-YAP (D8H1X) XP | Cell Signaling Technology | Cat# 14074, RRID: AB_2650491 |
| Rabbit Anti-Human Actin, Smooth Muscle Epitope Specific antibody | Thermo Fisher Scientific | Cat# RB-9010-P0, RRID: AB_149755 |
| Alexa Fluor 488 Goat Anti Mouse IgG (H+L) | Molecular Probes | Cat# A-11029, RRID: AB_138404 |
| Alexa Fluor 546 Goat Anti Mouse IgM (m chain) | Molecular Probes | Cat# A-21045, RRID: AB_2535714 |
| Alexa Fluor 546 Goat Anti Rabbit IgG (H+L) | Molecular Probes | Cat# A-11035, RRID: AB_143051 |
| Alexa Fluor 488 Goat Anti Rabbit IgG (H+L) | Molecular Probes | Cat# A-11034, RRID: AB_2576217 |
| Goat anti-Mouse IgG (H+L) Secondary Antibody, HRP | Thermo Fisher Scientific | Cat# 31430, RRID: AB_228307 |
| Goat anti-Rabbit IgG (H+L) Secondary Antibody, HRP | Thermo Fisher Scientific | Cat# 31460, RRID: AB_228341 |
| Bacterial and Virus Strains | | |
| N/A | N/A | N/A |
| Biological Samples | | |
| N/A | N/A | N/A |
| Chemicals, Peptides, and Recombinant Proteins | | |
| ROCK inhibitor Y27632 | Sigma-Aldrich | Y0503; CAS Number 129830-38-2 |
| CytochalasinD | Sigma-Aldrich | C8273; CAS Number: 22144-77-0 |
| Jasplakinolide | Santa Cruz Biotechnology | sc-202191; CAS 102396-24-7 |
| Caerulein | Sigma-Aldrich | C9026; CAS Number: 17650-98-5 |
| Critical Commercial Assays | | |
| Lipofectamine RNAiMAX Transfection Reagent | Invitrogene | Cat#10601435 |
| Alexa Fluor 647 Phalloidin | Invitrogene | Cat# A22287, RRID:AB_2620155 |
| Dual-Luciferase Reporter Assay System | Promega | Cat#E1910 |
| RNAeasy micro kit | QIAGEN | Cat#74004 |
| Omniscript RT kit | QIAGEN | Cat# 205111 |
| Random primers | Promega | Cat#C1181 |
| SYBR green | Applied Biosystems | Cat#4309155 |
| G-Actin/F-actin *In Vivo* Assay Biochem Kit | Cytoskeleton | Cat#BK037 |
| Deposited Data | | |
| RNaseq data | This paper | GEO: GSE120514 |
| Mass spectrometry data | Table S2 | N/A |

(*Continued on next page*)

*Continued*

| REAGENT or RESOURCE | SOURCE | IDENTIFIER |
|---|---|---|
| Experimental Models: Cell Lines | | |
| WT and Cav1KO Mouse embryonic fibroblasts (MEF) | Razani et al., 2001 | N/A |
| WT and Cav1KO Neonatal Hepatocytes (mouse) | Mayoral et al., 2010 | N/A |
| MDA-MB-231 HTB-26 breast adenocarcinoma (human) | ATCC | Cat# HTB-26, RRID: CVCL_0062 |
| HeLa CCL-2 | ATCC | Cat# CCL-2, RRID: CVCL_0030 |
| HeLa Cav1–GFP | Lukas Pelkmans laboratory | N/A |
| Primary pancreatic cancer associated fibroblasts (PanCAF) | Manuel Hidalgo Laboratory | N/A |
| Experimental Models: Organisms/Strains | | |
| Mouse: Cav1KO C57BL/6 | Drab et al., 2001 | N/A |
| Oligonucleotides | | |
| siRNA targeting sequence: Cav1 #1: GAGCUUCCUGAUUGAGAUU (sense sequence) | This paper | N/A |
| ON-Target Plus Smart-pool siRNAs; see Table S3 | Dharmacon | N/A |
| Primers for qRT-PCR | Table S4 | N/A |
| Recombinant DNA | | |
| p2xFlag CMV2-YAP2 | Oka et al., 2008 | Addgene #19045 |
| pCMV-flag YAP2 5SA | Zhao et al., 2007 | Addgene #27371 |
| 8xGTIIC-luciferase | Dupont et al., 2011 | Addgene #34615 |
| pLVX-CMV-CherryFP-P2A-MetLuc | This paper | N/A |
| pLVX_shRNA2 | Clontech | Cat#632179 |
| CMVCherryFPP2A | Viral vectors Unit (CNIC) | N/A |
| pMetLuc reporter | Viral vectors Unit (CNIC) | N/A |
| pEGFP–mDia1(DeltaN3) | Ishizaki et al., 2001 | N/A |
| pEGFP-RHO(V14) | del Pozo et al., 1999 | N/A |
| Software and Algorithms | | |
| qBase plus | Biogazelle | N/A |
| MATLAB (R2015a) | MATLAB | N/A |
| ImageJ | National Institutes of Health | https://imagej.nih.gov/ij/download.html |
| FibrilTool plug-in | Boudaoud et al., 2014 | https://media.nature.com/original/nature-assets/nprot/journal/v9/n2/extref/nprot.2014.024-S3.txt |
| Ingenuity Pathway Analysis software | QIAGEN | N/A |
| Enrichr | Enrichr - Ma'ayan Laboratory - Computational Systems Biology | http://amp.pharm.mssm.edu/Enrichr/ |
| Columbus Image Data Storage and Analysis System | PerkinElmer | http://www.perkinelmer.com/es/product/image-data-storage-and-analysis-system-columbus |
| Search-Based Exploration of Expression Compendium (SEEK) | Troyanskaya Functional Genomics Laboratory at Princeton University, 2014 | http://seek.princeton.edu/ |
| GraphPad Prism | GraphPad Software | https://www.graphpad.com/company/ |
| Fisher exact test calculator | Social Science Statistics web site | http://www.socscistatistics.com/tests/fisher/Default2.aspx |
| Other | | |
| pre-printed micropatterns | CYTOO | N/A |
| Fibronectin-coated 6-well plates for uniaxial stretching | FlexCell | Cat# UF-4001P |
| Flexcell FX-5000 Tension System | FlexCell | N/A |
| Protein G Sepharose 4 Fast Flow | GE Healthcare | Cat# GE17-0618-01 |

## CONTACT FOR REAGENT AND RESOURCE SHARING

Further information and requests for reagents may be directed to, and will be fulfilled by the Lead Contact, Miguel Ángel del Pozo (madelpozo@cnic.es).

## EXPERIMENTAL MODEL AND SUBJECT DETAILS

### *In vivo* animal studies

Cav1KO C57BL/6 mice (Drab et al., 2001) were bred under specific pathogen-free conditions at the CNIC. Experiments were performed with 8-12-week-old males (Cav1KO and age-matched control littermates). All animal protocols (PROEX 097/18) were in accordance with Spanish animal protection law and were authorized by the corresponding local authority.

### Cell culture

MEFs were isolated from WT and Cav1KO littermate mice, immortalized, and cultured as described (Razani et al., 2001). Neonatal hepatocytes from WT and Cav1KO littermates were isolated, phenotyped (Mayoral et al., 2010), and kindly provided by Dr. Martín-Sanz (IIBM Alberto Sols, Spain). The human MDA-MB-231 breast carcinoma and HeLa cell lines were obtained from ATCC. HeLa cells expressing CAV1–GFP were kindly provided by Lukas Pelkmans (ETH, Zürich, Switzerland). MEFs, hepatocytes and HeLa cells were grown in Dulbecco's modified Eagle's medium (DMEM) and MDA-MB231 cells were grown in DMEM/F-12 (GIBCO, Thermo Fisher Scientific [Waltham; Massachusetts, United States]); growth media were supplemented with 10% fetal bovine serum (FBS; GIBCO, Thermo Fisher Scientific and GE Healthcare Life Science HyClone [Little Chalfont, United Kingdom]) and 100 μg/ml penicillin and streptomycin (GIBCO, Thermo Fisher Scientific). Primary pancreatic cancer associated fibroblasts (PanCAF) were a gift from Manuel Hidalgo (Centro Nacional de Investigaciones Oncológicas, Spain). PanCAFs were grown in Roswell Park Memorial Institute medium (RPMI) supplemented with 20% FBS, 100 μg/ml penicillin and streptomycin, and 5% glutamine. All cells were maintained in a humidified atmosphere at 37 °C and 5% CO2.

## METHOD DETAILS

### Polyacrylamide matrices

Polyacrylamide gels with tuneable stiffness were prepared on glass coverslips as previously described (Tse and Engler, 2010). 3-aminopropyltrimethoxysilane (Sigma-Aldrich [St. Louis, Missouri, United States]) was applied over the surface of a coverslip using a cotton-tipped swab and another coverslip was treated with Sigmacote® (Sigma-Aldrich). The coverslips were then washed thoroughly with sterilized water and dried. Acrylamide/bis-acrylamide solutions were prepared using appropriate concentrations to obtain stiff matrices (Young's modulus ~55 KPa) and soft matrices (~200 Pa) as previously defined (Fischer et al., 2012). Polymerization initiators (0.05% w/v ammonium persulfate and 0.0005% v/v N,N,N′,N′-tetramethylethylenediamine [TEMED], final concentrations) were added to the bis-acrylamide mixture. A drop of this mixture was deposited on top of the silanized glass and covered with the sigmacote-treated coverslip; 183 μL was deposited for round coverslips (40mm diameter) and 50 μL for square coverslips (24x24mm). After polymerization, the upper coverslip was removed and the polyacrylamide surface was photo-activated by exposing the sulfo-SANPAH crosslinker (Sigma-Aldrich) to UV light. Finally, the surface was coated with fibronectin (5 μg/ml) for 1 h at 37°C. Fibronectin was then removed and cells were seeded at low confluence. Experiments were performed 24h after seeding.

### Micropatterns

Glass slides with pre-printed micropatterns were purchased from Cytoo (Grenoble, France). Designs for customized patterns with specific grid sizes were described by Dr. Piccolo and colleagues (Dupont et al., 2011). Fibronectin coating was performed as specified by the supplier. Cells were plated, and 24 h later, fixed and stained following standard protocols.

### Cell strain

24h after plating on fibronectin-coated 6-well plates (FlexCell [Burlington, North Carolina, United States]), cells were subjected to uniaxial cyclic stretching (0.7Hz, 8%–9% amplitude) for 24h on a programmable Flexcell® FX-5000™Tension System (FlexCell) under standard culture conditions.

### Reagents and transfections

The ON-TARGET plus SMARTpool siRNAs were purchased from Dharmacon (Table S3; Lafayette, Colorado, United States), siRNA targeting human CAV1 was custom made (sense sequence: GAGCUUCCUGAUUGAGAUU. Cells were transfected with siRNAs at 20pmol/1000 cells using Lipofectamine® RNAiMAX (Invitrogene; Carlsbad, California, United States). Silencing was allowed to proceed for 48h before terminating the experiment.

p2xFlag CMV2-YAP2 was a gift from Dr. Sudol (Addgene plasmid # 19045; Cambridge, Massachusetts, USA) (Oka et al., 2008). pCMV-flag YAP2 5SA (Addgene plasmid # 27371) was a gift from Dr. Guan. 8xGTIIC-luciferase (Addgene plasmid # 34615) was a gift from Dr. Piccolo. The lentiviral backbone for pLVX-CMV-CherryFP-P2A-MetLuc was derived from pLVX_shRNA2 (Clontech;

Mountain View, California, United States) and was provided by the CNIC Viral Vectors (VV) Unit. CMVCherryFPP2A was obtained from pRRL_CMV_CherryFP_P2A (provided by the CNIC VV Unit and cloned into pLVX). The Metridia luciferase (secretable form) was amplified from pMetLuc reporter (Clontech; also provided by the CNIC VV Unit) and cloned in-frame with the CherryFP-P2A peptide. pEGFP–mDia1(DeltaN3) and pcDNA3-HA-RHO(V14) were as described (del Pozo et al., 1999; Ishizaki et al., 2001). All transient transfections were by electroporation with 5 μg plasmid DNA and 35 μg UltraPure salmon sperm DNA solution (Sigma-Aldrich) at 350V and 550ohms for 10msec.

Drugs were added to cells 3h after plating, followed by incubation for a further 21h. The ROCK inhibitor Y27632 (Y0503) and cytochalasinD (C8273) were from Sigma-Aldrich. Jasplakinolide (sc-202191) was from Santa Cruz Biotechnology (Dallas, Texas, United States).

### Antibodies

Monoclonal antibodies were sourced as follows: anti-YAP (sc-101199) and anti-TEF-1 (sc-376113) from Santa Cruz Biotechnology; anti-CAV1 XP (#3267) and anti-YWHAH (14-3-3 η (D23B7); #5521) from Cell Signaling (Danvers, Massachusetts, United States); anti-Flag M2 (F-3167) from Sigma-Aldrich; and anti-glyceraldehyde-3-phosphate dehydrogenase (MAB374) and anti-cortactin (p80/85, clone 4F11) from Millipore (Burlington, Massachusetts, United States). Polyclonal antibodies to phospho-YAP (Ser127) (#9411), LATS1 (#9153), and LATS2 (#13646) were from Cell Signaling; anti-Histone H3 (ab1791) was from Abcam (Cambridge, United Kingdom); and anti-RHO GDI (sc-360) was from Santa Cruz Biotechnology. For immunohistochemistry, we used anti-YAP (D8H1X) XP from Cell Signaling (#14074) and αSMA from Thermo Fisher Scientific (RB-9010-P0).

### Immunofluorescence microscopy

For immunofluorescence procedures, cells were fixed in paraformaldehyde 4% (w/v) at 37°C for 10 minutes, permeabilized, blocked with 0.2% Triton X-100 in BSA 1% (w/v) for 10 min, and then immunostained with specific antibodies for 1h. Alexa647 phalloidin and Alexa546- and Alexa488-labeled secondary antibodies were from Invitrogen. Images were acquired either on a Zeiss LSM700 confocal microscope or an Opera automated confocal microscope (PerkinElmer; Waltham, Massachusetts, United States).

### Subcellular fractionation

For subcellular fractionation the cells were lysed (10 mM HEPES, pH 7.6, 10 mM KCl, 0.1 mM EDTA, 0.1 mM EGTA, 0.5 mM DTT, 100 mM phenylmethylsulfonyl fluoride, protease inhibitor cocktail [Roche], and 0.05% NP-40). Nuclear and cytoplasmic fractions were separated by centrifugation. The cytosolic fraction was precipitated with acetone and nuclei were lysed (20 mM HEPES, pH 7.6, 0.4 M NaCl, 1 mM EDTA, 1 mM EGTA, 1 mM DTT, 0.75 mM spermidine, 0.15mM spermine,100 mM phenylmethylsulfonyl fluoride, and protease inhibitor cocktail [Roche]) and centrifuged at 13000 rpm to remove the DNA. Both fractions were eluted with sample buffer and analyzed by western blotting.

### Immunoprecipitation

For immunoprecipitation, cells were lysed (50mM Tris-HCl at pH8, 100mM NaCl, 1% Triton X-100 [Sigma-Aldrich], 10% glycerol, 1mMMgCl2, 2mM PMSF, protease inhibitor cocktail [Roche]). Cell lysates were centrifuged for 10 min at 4°C. Supernatants were mixed with the specific antibody or control IgG for 2h, and protein G–agarose beads were added for a further 2h. Beads were washed with washing buffer (50mM Tris-HCl pH7.5, 150mM NaCl, 1mM EDTA, 0.25% gelatin, 0.1% NP-40 [Sigma-Aldrich]) and processed for mass spectrometry or western blotting.

### Immunoblotting

For western blotting, immunoprecipitated proteins were eluted with sample buffer and analyzed by western blotting on nitrocellulose membranes (Amersham Pharmacia Biotech, UK) with primary and secondary HRP-conjugated antibodies using standard protocols. Proteins were detected by enhanced chemiluminescence (Amersham Life Sciences; Arlington Hts, Illinois, United States). Nuclear and cytosolic subcellular fractions were prepared as described (Navarro-Lérida et al., 2015).

### Image analysis

YAP subcellular distribution was analyzed with the Columbus Image Data Storage and Analysis System (Perkin Elmer) or imageJ. Nuclei were segmented using the Hoechst signal (Figure S4B). Mitotic and aberrant nuclei were then eliminated based on Hoechst intensity and nuclear roundness and area. Cells located at the image borders were also eliminated. The cytosol was segmented growing the nuclear segmentation (Figure S4C). The cytosolic ROI for cytosolic YAP intensity calculation was built as a 4 pixel ring of cytoplasm grown radially from the segmented nuclear border (Figure S4C). Finally, the ratio between nuclear and cytosolic YAP was calculated.

### Second harmonic generation (SHG) imaging

Fibrillary collagen in non-fixed cell-embedded collagen gels was imaged using the SHG technique (Chen et al., 2012) with a Zeiss LSM780 multiphoton microscope (Carl Zeiss Microscopy; Jena, Germany) fitted with a short pulse laser.

## Luciferase assay

Luciferase assays to monitor TEAD transcriptional activity with the 8xGTIIC-luciferase reporter were as described (Mahoney et al., 2005). Cells were transiently co-transfected with 8xGTIIC-luciferase (product: firefly luciferase) and pLVX-CMV-CherryFP-P2A-MetLuc (product: Metridia luciferase [MelLuc], which is secreted to the medium). Luciferase activity was monitored with the Dual-Luciferase® Reporter Assay System (Promega; Madison, Wisconsin, United States) in an ORION II microplate luminometer (Titertek Berthold; Bad Wildbad, Germany). Firefly luciferase was quantified in cell lysates by adding Luciferase Assay Reagent II (LARII), and MetLuc was quantified in culture medium by adding Stop & Glo reagent. Firefly luciferase activity was normalized to MetLuc activity to control for variability in transfection efficiency across samples.

## Collagen contraction assay

Contraction assays to monitor matrix remodeling were as described (Goetz et al., 2011; Orimo et al., 2005). Briefly, $1.5 \times 10^5$ MEFs were included in a collagen type I gel (PureCol, Sigma-Aldrich; 1.5mg/ml, 500 $\mu$L total volume) in an Ultra-Low Attachment 24-well plate (Corning; Corning, New York, United States). After gel polymerization, normal culture medium was added, and collagen gel borders were detached from the border of the plate. Gels were cultured at 37 °C, 5% $CO_2$ for 48h. Gel contraction was monitored by quantifying the gel surface area on photographs with ImageJ. The fold-change with respect to the contraction observed in a control condition was calculated for each sample.

## Acute pancreatitis induction

Acute pancreatitis was induced by caerulein treatment as described (Guerra et al., 2007). Before the experiment, mice were starved for 12h with unrestricted access to drinking water. Acute pancreatitis was induced by 7 intraperitoneal injections of caerulein (Sigma-Aldrich) dissolved in PBS; injections were given at 1-h intervals on 2 consecutive days at a dose of 50 $\mu$g caerulein /kg body weight per injection. Control animals received injections of PBS only. At defined intervals, animals were sacrificed and the pancreas excised for immunohistochemical analysis. Immunostained preparations were scanned with Hamamatsu Nanozoomer 2.0 RS (Hamamatsu, Japan) and digitized with NDP.scan 2.5. Images were viewed and quantified with NDP.analyzer and NDP.view2 (Hamamatsu; Hamamatsu City, Japan).

## Real-time quantitative PCR

RNA was extracted from cell samples with the RNAeasy micro kit (QIAGEN; Hilden, Germany). For each sample, 1 $\mu$g RNA was reverse transcribed using the Omniscript RT kit (QIAGEN) and random primers (Promega). qPCR was performed with SYBR green (Applied Biosystems; Foster City, California, USA) Appropriate negative and positive controls were used (Hellemans et al., 2007). Results were normalized to endogenous GAPDH and HPRT1 expression using qBase plus. Primer sequences were summarized in Table S4.

## RNA-Seq analysis

Next generation sequencing experiments were performed at the CNIC Genomics Unit. Total RNA was extracted as for qRT-PCR (see above). RNA integrity was determined with an Agilent 2100 Bioanalyzer (Agilent Technologies; Santa Clara, California, United States). Two RNA samples per condition were analyzed by single read (SR) sequencing in an Illumina HiSeq 2500 System (Illumina; San Diego, California, United States). Data were analyzed in the CNIC Bioinformatics Unit. Enrichment analysis was conducted using Ingenuity Pathway Analysis software (IPA, QIAGEN) and the Enrichr web tool.

## Actin fiber organization analysis

Actin fiber organization was imaged and quantified in MATLAB (R2015a) and ImageJ (1.51a x64). Fiber order/parallelism in a ROI was measured with a custom multiscale anisotropy analysis script based on the FibrilTool plug-in for ImageJ (Boudaoud et al., 2014); this tool analyzes the structural information embodied by the eigenvalues and eigenvectors of a nematic tensor. Iterative performance of this analysis in a collection of image patches of different sizes gives information about the organization of structures at multiple scales and locations. The anisotropy value of each pixel in the original image is set to the mean anisotropy score obtained for each processed patch containing the pixel. The patch collection is created by dividing the image into NxN nonoverlapping subimages, with $N \in \{2^i | i = 0, 1, 2\ldots\} \cup \{3^i | i = 0, 1, 2\ldots\}$ defining each iteration. The final multiscale anisotropy score is the mean of all the anisotropy values of the pixels in the image. This measurement can be constrained to a specific area of interest by using only specific pixels determined by prior segmentation, a useful way of avoiding interference from background or other structures that could bias the results. In micropattern experiments, the inputs were cropped images of the cytoplasmic area of cells (241x261 pixels), avoiding background and membranes as much as possible; no prior area restriction was defined, and the minimum size of patches was fixed at 10x10 pixels.

## Mass spectrometry analysis

Protein G–agarose beads bound to immunoprecipitated proteins were incubated at room temperature for 2h in a 60 $\mu$L volume of 2 M urea, 50 mM Tris-HCl pH 8.5, and 10 mM TCEP with gentle vortexing. Iodoacetamide (7 $\mu$L, 500 mM) was then added and the incubation continued in the dark. After dilution to 0.5 M urea with ammonium bicarbonate, 3 $\mu$g of trypsin were added and samples were

incubated for 6-8 h at 37°C. Samples were then acidified to 1% TFA, and the supernatants were desalted on C18 minispin columns (The Nest Group; Southborough, MA, USA) and dried down for further analysis. Experiments were performed with 5 independent replicates. Peptides were analyzed by LC-MS/MS using a C-18 reversed phase nano-column (75 μm I.D. x 50 cm, 2 μm particle size, Acclaim PepMap RSLC, 100 C18; Thermo Fisher Scientific) in a continuous acetonitrile gradient consisting of 0%–32% B over 80 min, 50%–90% B over 3 min at 50°C (A = 0.1% formic acid; B = 80% acetonitrile, 0.1% formic acid). Peptides were eluted from the nanocolumn at a flow rate of 200 nL/min to an emitter nanospray needle for real-time ionization and peptide fragmentation in a QExactive HF mass spectrometer (Thermo Fisher Scientific). The chromatographic run analyzed an enhanced FT-resolution spectrum (70,000 resolution) followed by the MS/MS spectra from the 15 most intense parent ions. Dynamic exclusion was set at 40 s. For peptide identification, all spectra were analyzed with Proteome Discoverer (version 2.1.0.81, Thermo Fisher Scientific) using SEQUEST-HT (Thermo Fisher Scientific). For searching the Uniprot proteome database containing all sequences from mouse and frequently observed contaminants (April 27, 2016; 48644 entries), the following parameters were selected: trypsin digestion with 2 maximum missed cleavage sites; precursor and fragment mass tolerances of 2 Da and 0.02 Da, respectively; carbamidomethyl cysteine as a fixed modification; and methionine oxidation as a dynamic modification. Peptides were identified by the probability ratio method (Martínez-Bartolomé et al., 2008), and false discovery rate (FDR) was calculated using inverted databases and the refined method (Navarro and Vázquez, 2009) with an additional filtering for a precursor mass tolerance of 15 ppm (Bonzon-Kulichenko et al., 2015). Proteins were quantified for each condition based on the number of scans/peptides identified at 1% FDR.

### Image-based siRNA screening

The smart-pool siRNA library for selected YAP interactors detected by mass spectrometry was purchased from Dharmacon. Four different sequences per each gene were used. SiRNAs were transfected by reverse transfection in 384-well plates. Cells were fixed and stained for YAP detection 48h post transfection as described previously. Immunofluorescence images were acquired with an Opera automated confocal microscope (PerkinElmer). Three replicates were performed, with four wells per siRNA in each replicate. Two different ON-traget nontargeting siRNA controls were used. Transfection efficiency was validated by transfection with INCENP siRNA, which promotes the appearance of multinucleated cells and cells with aberrant nuclei (Figure S4A). YAP nucleo:cytosolic ratios were calculated using Columbus as described above (Image analysis section), and Z-scores were calculated as Z = (x – control mean)/control standard deviation. The mean Z-score of the three replicates was calculated.

### SEEK analysis

SEEK (http://seek.princeton.edu) is a computational coexpression gene search tool (Zhu et al., 2015). We queried this web tool with the list of previously published YAP target genes (Dupont et al., 2011) and used all human expression datasets from tissue samples and cell lines included in SEEK for coexpression analysis. The program gives a ranked list of genes ordered from the strongest positive correlation with the query to the weakest. With Enrichr (Chen et al., 2013; Kuleshov et al., 2016), we analyzed the enrichment in KEGG annotated pathways and gene ontology (GO) terms of the 200 genes showing the highest positive coexpression with YAP targets.

## QUANTIFICATION AND STATISTICAL ANALYSIS

Statistical details of experiments are reported in Figure Legends. Significance was evaluated by paired Student's t test, using GraphPad Prism. Differences were considered statistically significant at *p < 0.05, **p < 0.01, ***p < 0.005, and ****p < 0.0005. YAP-target gene enrichment on stiff versus soft substrates in the RNA-Seq analysis was compared by the Fisher exact test using an online Fisher exact test calculator (http://www.socscistatistics.com/tests/fisher/Default2.aspx).

## DATA AND SOFTWARE AVAILABILITY

The accession number for the RNA-seq data reported in this paper is GEO: GSE120514.

Cell Reports, Volume *25*

## Supplemental Information

# Caveolin-1 Modulates Mechanotransduction

# Responses to Substrate Stiffness

# through Actin-Dependent Control of YAP

**Roberto Moreno-Vicente, Dácil María Pavón, Inés Martín-Padura, Mauro Català-Montoro, Alberto Díez-Sánchez, Antonio Quílez-Álvarez, Juan Antonio López, Miguel Sánchez-Álvarez, Jesús Vázquez, Raffaele Strippoli, and Miguel A. del Pozo**

# Figure S1

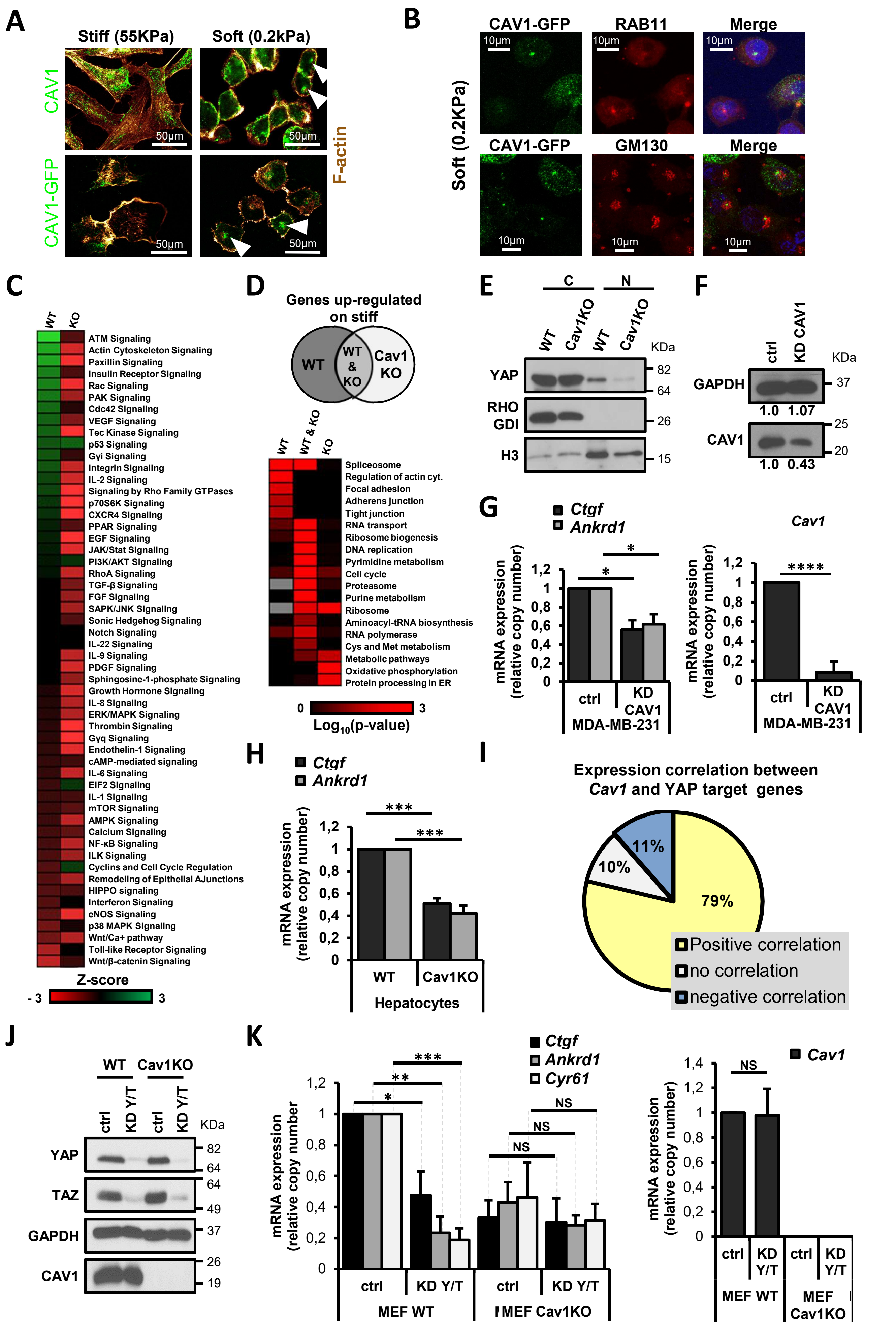

A
Stiff (55KPa)
Soft (0.2kPa)
CAV1
CAV1-GFP
F-actin
50µm
B
Soft (0.2KPa)
CAV1-GFP
RAB11
Merge
GM130
10µm
C
WT
KO
ATM Signaling
Actin Cytoskeleton Signaling
Paxillin Signaling
Insulin Receptor Signaling
Rac Signaling
PAK Signaling
Cdc42 Signaling
VEGF Signaling
Tec Kinase Signaling
p53 Signaling
Gγi Signaling
Integrin Signaling
IL-2 Signaling
Signaling by Rho Family GTPases
p70S6K Signaling
CXCR4 Signaling
PPAR Signaling
EGF Signaling
JAK/Stat Signaling
PI3K/AKT Signaling
RhoA Signaling
TGF-β Signaling
FGF Signaling
SAPK/JNK Signaling
Sonic Hedgehog Signaling
Notch Signaling
IL-22 Signaling
IL-9 Signaling
PDGF Signaling
Sphingosine-1-phosphate Signaling
Growth Hormone Signaling
IL-8 Signaling
ERK/MAPK Signaling
Thrombin Signaling
Gγq Signaling
Endothelin-1 Signaling
cAMP-mediated signaling
IL-6 Signaling
EIF2 Signaling
IL-1 Signaling
mTOR Signaling
AMPK Signaling
Calcium Signaling
NF-κB Signaling
ILK Signaling
Cyclins and Cell Cycle Regulation
Remodeling of Epithelial AJunctions
HIPPO signaling
Interferon Signaling
eNOS Signaling
p38 MAPK Signaling
Wnt/Ca+ pathway
Toll-like Receptor Signaling
Wnt/β-catenin Signaling
Z-score
-3
3
D
Genes up-regulated on stiff
WT
WT & KO
Cav1 KO
WT
WT & KO
KO
Spliceosome
Regulation of actin cyt.
Focal adhesion
Adherens junction
Tight junction
RNA transport
Ribosome biogenesis
DNA replication
Pyrimidine metabolism
Cell cycle
Proteasome
Purine metabolism
Ribosome
Aminoacyl-tRNA biosynthesis
RNA polymerase
Cys and Met metabolism
Metabolic pathways
Oxidative phosphorylation
Protein processing in ER
0
3
Log10(p-value)
E
C
N
WT
Cav1KO
WT
Cav1KO
KDa
YAP
RHO GDI
H3
82
64
26
15
F
ctrl
KD CAV1
KDa
GAPDH
1.0 1.07
37
CAV1
1.0 0.43
25
20
G
Ctgf
Ankrd1
Cav1
mRNA expression (relative copy number)
1,2
1
0,8
0,6
0,4
0,2
0
*
*
****
ctrl
KD CAV1
MDA-MB-231
H
Ctgf
Ankrd1
***
***
mRNA expression (relative copy number)
WT
Cav1KO
Hepatocytes
I
Expression correlation between Cav1 and YAP target genes
11%
10%
79%
Positive correlation
no correlation
negative correlation
J
WT
Cav1KO
ctrl
KD Y/T
KDa
YAP
TAZ
GAPDH
CAV1
82
64
64
49
37
26
19
K
Ctgf
Ankrd1
Cyr61
***
**
*
NS
NS
NS
mRNA expression (relative copy number
1,4
ctrl
KD Y/T
MEF WT
MEF Cav1KO
Cav1
mRNA expression (relative copy number)

**Figure S1. Cav1 controls the expression of cytoskeletal regulatory genes and YAP targets. (Related to Figure 1) (A)** Conflocal immunofluorencence images of CAV1 and phalloidine staining in HeLa cells (upper panels) and CAV1-GFP expressing cells (lower panels) grown on stiff or soft substrates for 24 hours. Arrowheads mark the intracellular accumulation of CAV1 in soft conditions. **(B)** Conflocal immunofluorencence images of CAV1-GFP, RAB11 (recycling endosome marker) and GM130 (Golgi marker). **(C)** Computational analysis using IPA software to detect canonical pathways regulated by matrix stiffness. mRNA expression in cells on rigid (55KPa) or compliant (0.22KPa) polyacrylamide hydrogels was assessed by RNA-Seq. Genes significantly up- or down-regulated by matrix stiffness in WT or Cav1KO MEFs were analyzed. Activation Z-scores are color-coded for each genotype. Positive values (green) indicate activation after exposure to rigid substrate, whereas negative values (red) indicate pathway inhibition. **(D)** *Top,* Venn diagram showing genes up-regulated in WT and Cav1KO MEFs grown on stiff substrates in the RNA-Seq study. *Bottom,* KEGG-term enrichment between genes up-regulated in stiff conditions in WT MEFs, Cav1KO MEFS, and both genotypes (bottom). **(E)** YAP western blot in nuclear and cytosolic fractions of WT and Cav1KO MEFs grown on stiff substrates. RHO-GDI and Histone H3 were used as cytosolic and nuclear internal controls. **(F)** Western blot of CAV1 in MDA-MB-231 cells transfected with CAV1 or control siRNAs. **(G)** qRT-PCR analysis of *Ctgf*, *Ankrd1*, and *Cav1* mRNA expression in MDA-MB-231 cells transfected with control or CAV1 siRNAs. Data are normalized to the control condition (n=4). **(H)** qRT-PCR analysis of *Ctgf* and *Ankrd1* mRNA expression in WT and Cav1KO mouse neonatal hepatocytes. Data are normalized to values in WT cells for each experiment. n = 4. **(I)** Relative distributions of YAP target genes showing positive, negative, or zero expression correlation with *Cav1* across 300 cell lines (p-value > 0.05). **(J and K)** Western blot **(J)** and qRT-PCR **(K)** analysis of YAP targets (*Ctgf*, *Ankrd1*, and *Cyr61*) and *Cav1* expression in cells transfected with control or YAP/TAZ-targeting siRNAs. For qRT-PCR analysis, data are normalized to WT cells grown on stiff substrate. n=3. Data are presented as means ±SEM; *P<0.05, **P<0.01, ***P<0.005.

# Figure S2

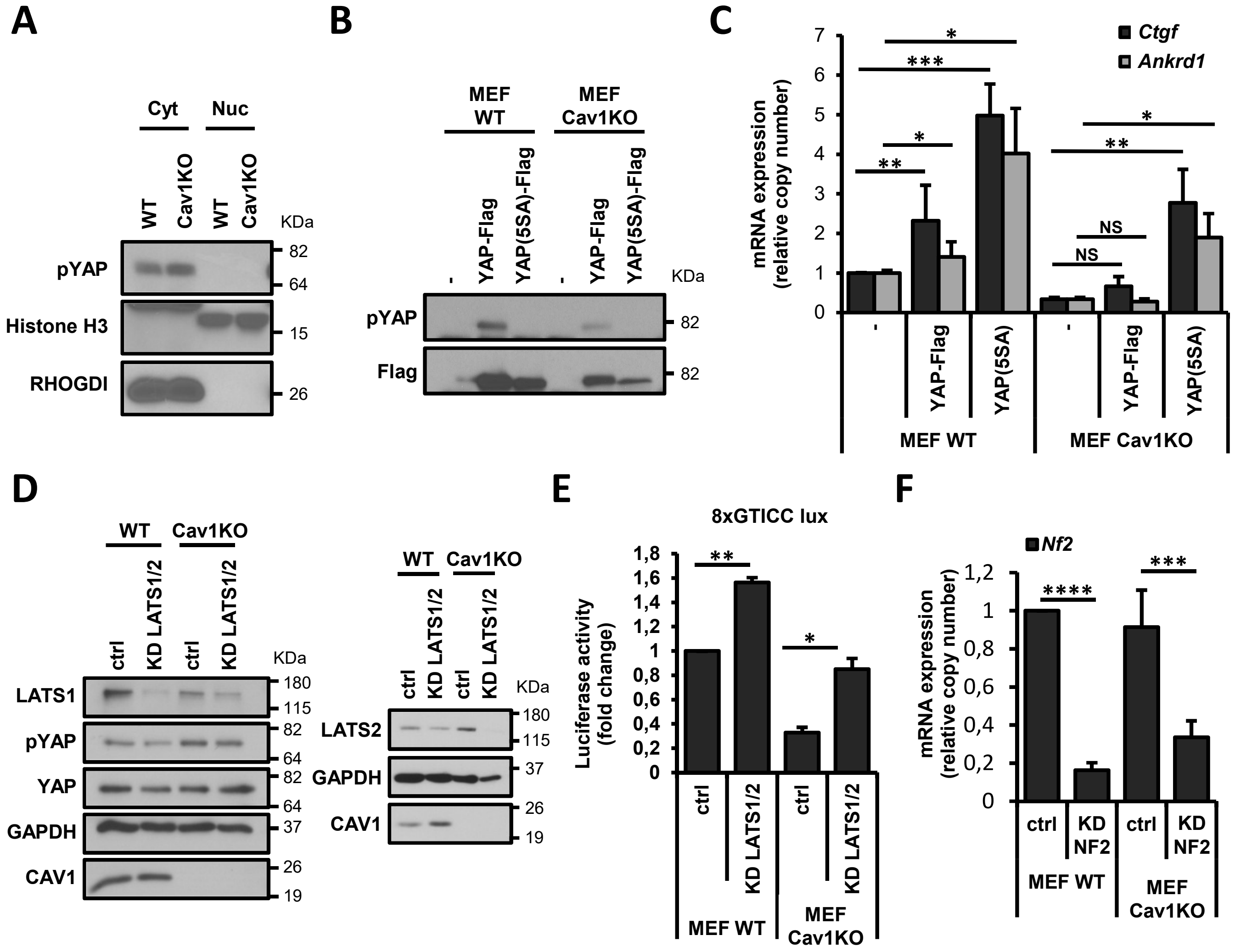


**Figure S2. Hippo-kinase-independent YAP phosphorylation determines deficient YAP activity in Cav1KO cells. (Related to Figure 2) (A)** Western blot analysis of the subcellular distribution of YAP phosphorylated on Ser112. Nuclear and cytosolic fractions were separated by biochemical fractionation. RHO-GDI and Histone H3 were used as cytosolic and nuclear internal controls. **(B)** Western blot for Flag in WT and Cav1KO MEFS transfected with YAP-Flag or YAP(5SA)-Flag. YAP phosphorylated on Ser112 migrated to the same position as the YAP constructs (which are larger than endogenous YAP) **(C)** qRT-PCR analysis of *Ctgf* and *Ankrd1* expression in cells transfected with YAP-Flag or YAP(5SA)-Flag. Data are normalized to WT control. n=4. **(D)** Western blot for LATS1 and 2, CAV1, S112-phosphorylated YAP, and total YAP in MEFs transfected with control or LATS1/2 siRNAs. GAPDH was used as an internal control. **(E)** TEAD transcriptional activity measured by 8xGTICC-luciferase reporter assay in cells transfected with control or LATS1/2 siRNAs. Data are normalized to WT control. n=3. **(F)** qRT-PCR analysis of *Nf2* expression in WT and Cav1 KO MEFs transfected with control or NF2 siRNAs. Data are normalized to WT control. n=4. Data are presented as means ±SEM. *P<0.05, **P<0.01, ***P<0.005, ****P<0.0005.

# Figure S3

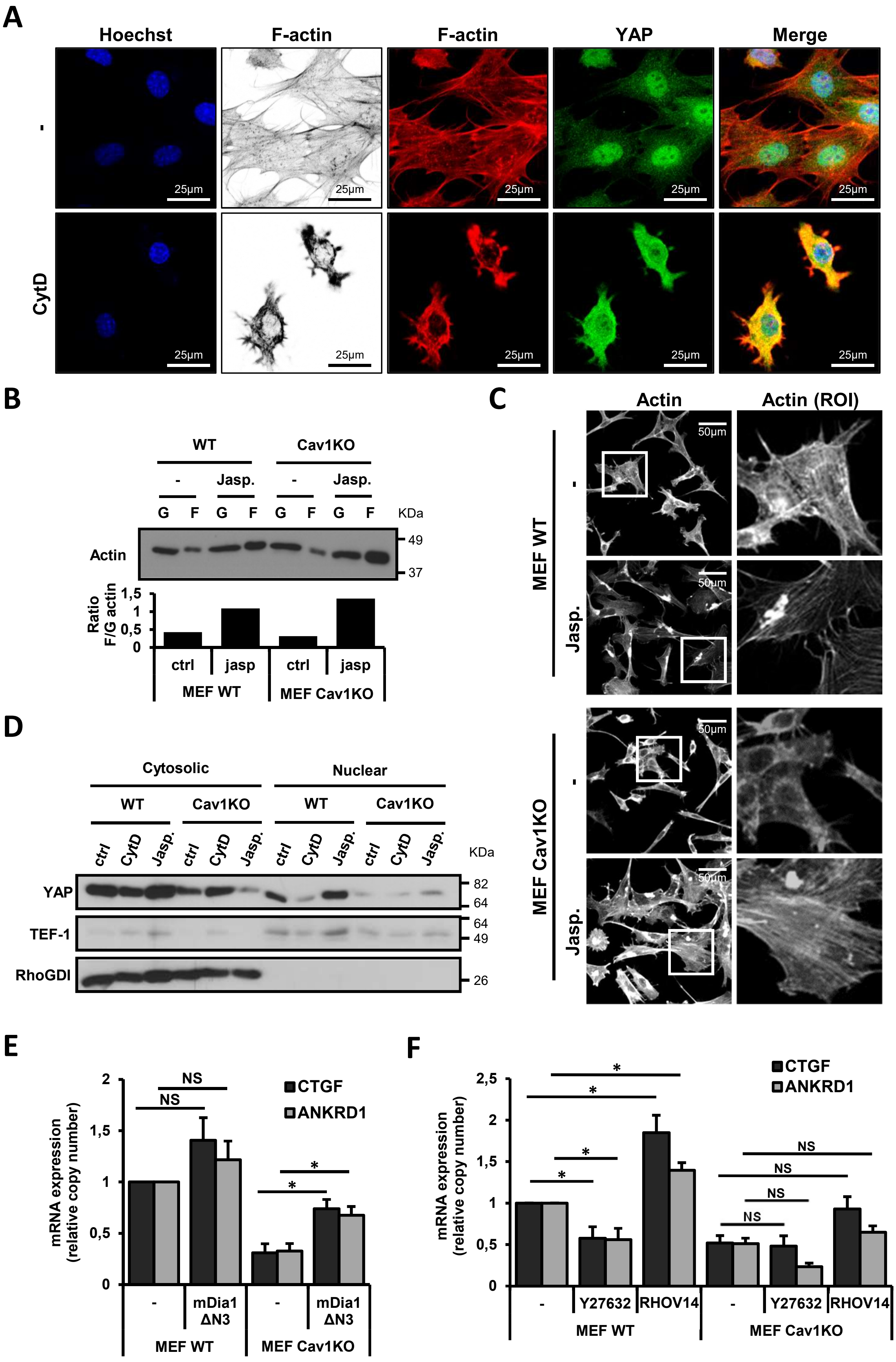

A
Hoechst
F-actin
F-actin
YAP
Merge
-
CytD
25µm
B
WT
Cav1KO
-
Jasp.
G F G F G F G F
KDa
Actin
49
37
Ratio F/G actin
1,5
1
0,5
0
ctrl
jasp
MEF WT
MEF Cav1KO
C
Actin
Actin (ROI)
MEF WT
MEF Cav1KO
Jasp.
50µm
D
Cytosolic
Nuclear
ctrl
CytD
Jasp.
YAP
TEF-1
RhoGDI
82
64
49
26
E
mRNA expression (relative copy number)
NS
*
CTGF
ANKRD1
mDia1 ΔN3
F
2,5
Y27632
RHOV14

**Figure S3. Altered actin dynamics, but not RhoA function, are a direct cause of the reduced YAP activity in Cav1KO MEFs (Related to Figure 3) (A**) Conflocal immunofluorencence images of YAP and phalloidin staining in cells treated for 24 hours with CytD (1μM) or DMSO. **(B)** Western blot for filamentous (F) and globular (G) actin in MEFs treated for 24 hours with 0.05μM jasplakinolide (Jasp.) or DMSO. F and G-actin were separated using an in vitro assay kit. Bottom graph showing the ratio between filamentous and globular actin. **(C)** Phalloidin staining in cells treated for 24 hours with 0.05μM jasplakinolide (Jasp.) or DMSO. Zoomed views of the selected ROIs (white squares) are shown on the right. YAP immunostaining is shown in **Figure 3E**. **(D)** YAP subcellular distribution in WT and Cav1KO MEFs treated for 24 hours with 1 μM Cytochalasin D (CytD) or 0.05μM jasplakinolide (jasp). Nuclear and cytosolic fractions were separated by biochemical fractionation. RHO-GDI and TEF-1 were used as cytosolic and nuclear markers, respectively. **(E)** qRT-PCR analysis of *Ctgf* and *Ankrd1* in cells transfected with a constitutively active form of *DIAPH1* (mDia1ΔN3) or mock-transfected. Data are normalized to WT mock-transfected. n =5. **(F)** qRT-PCR analysis of *Ctgf* and *Ankrd1* in cells transfected with a constitutively active RHOA mutant (RHOV14) or treated with the ROCK inhibitor Y27632 for 24 hours. Data are normalized to WT control. n=4. Data in **E** and **F** are presented as means ±SEM. *P<0.05.

# Figure S4

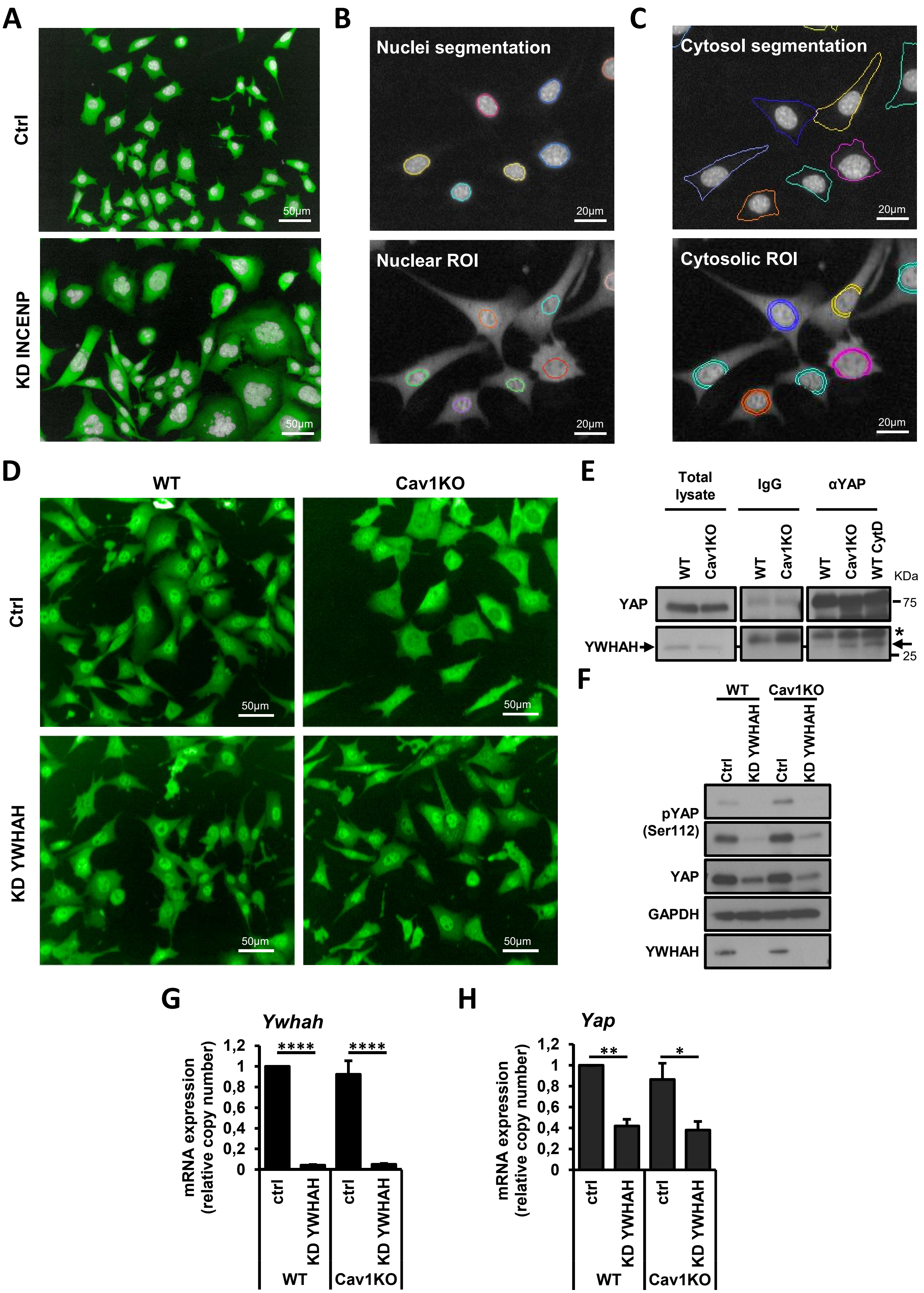

A
Ctrl
KD INCENP
50µm
B
Nuclei segmentation
Nuclear ROI
20µm
C
Cytosol segmentation
Cytosolic ROI
20µm
D
WT
Cav1KO
Ctrl
KD YWHAH
50µm
E
Total lysate
IgG
αYAP
WT
Cav1KO
WT CytD
KDa
YAP
YWHAH
75
25
*
F
WT
Cav1KO
Ctrl
KD YWHAH
pYAP (Ser112)
YAP
GAPDH
YWHAH
G
Ywhah
mRNA expression (relative copy number)
1,2
1
0,8
0,6
0,4
0,2
0
****
ctrl
KD YWHAH
WT
Cav1KO
H
Yap
**
*

**Figure S4. Functional analysis of YAP interactors by image-based screening. (Related to Figure 4) (A)** Representative images of YAP staining in cells transfected with control or INCENP siRNAs from the image-based screen. **(B)** Example of nuclear segmentation with Columbus™ using Hoechst staining (top) and the ROI used for YAP nuclear intensity calculation based on that segmentation (bottom). **(C)** Cytosolic segmentation with Columbus (top) and the ROI around the nuclei used for YAP cytosolic intensity calculation (bottom). **(D)** Representative images of YAP staining in WT and Cav1KO MEFs transfected with control siRNAs or YWHAH siRNAs identified in the image-based screen. **(E)** Co-immunoprecipitation of YWHAH with anti-YAP in WT and Cav1KO MEFs treated for 24 h with or without 1μM CytD. Negative controls (IgG) were performed in parallel, using a nonspecific IgG instead of anti-YAP antibody. The asterisk marks a nonspecific band. **(F)** Western blot analysis of WT and Cav1KO cells transfected with control or YWHAH siRNAs. **(G and H)** qRT-PCR analysis of *Ywhah* **(G)** and *Yap* **(H)** in WT and Cav1KO MEFs transfected with control or YWHAH siRNAs. n=5. Data are represented as means ±SEM. *P<0.05, **P<0.01, ****P<0.0005.

# Figure S5

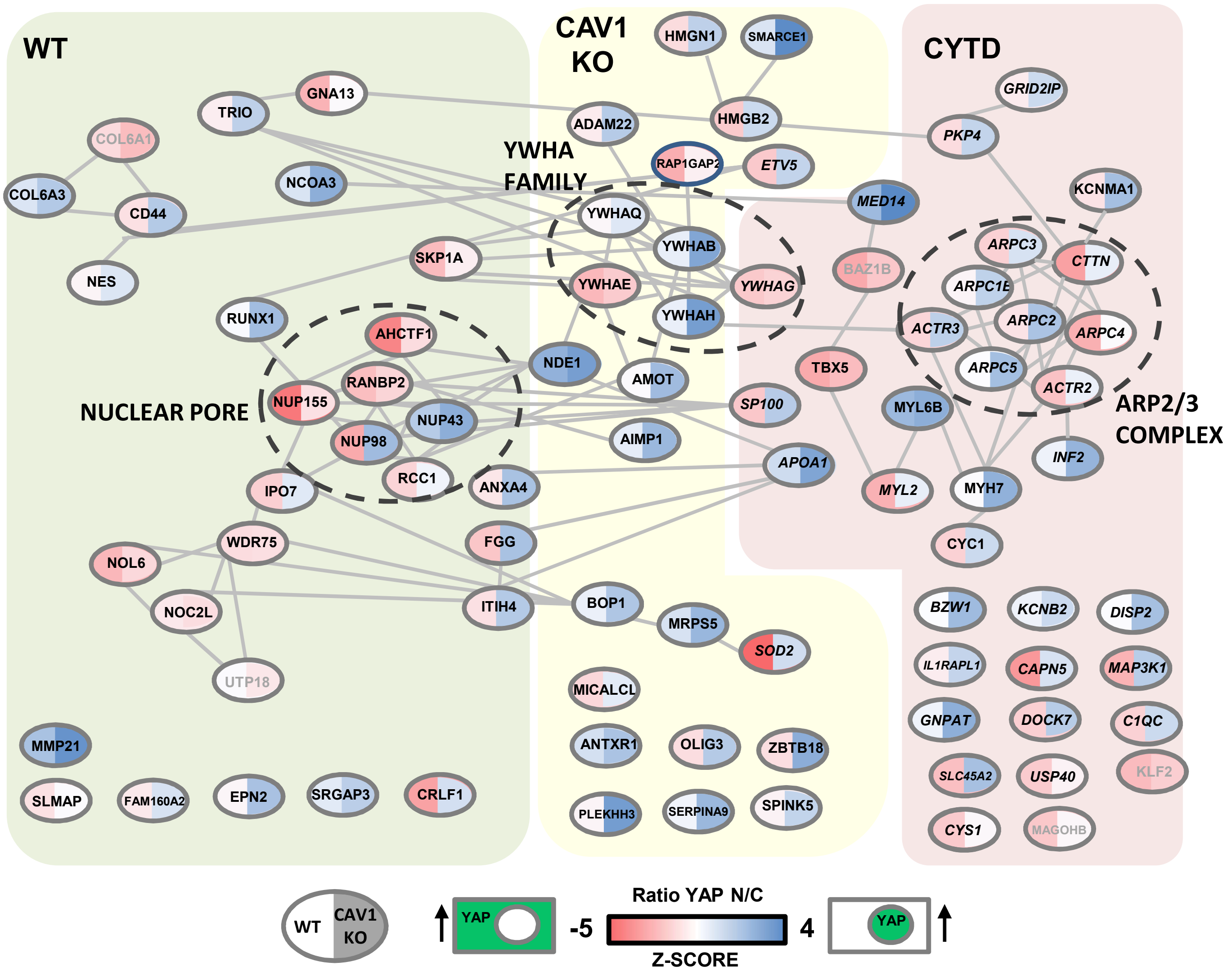


**Figure S5. YAP interactome characterization in WT and Cav1KO MEFs. (Related to Figure 4)** Functional protein association network based on data from STRING (Szklarczyk et al., 2017), including YAP interactors identified by mass spectrometry in untreated WT MEFs (green region) and Cav1KO MEFs (yellow region) and WT or Cav1KO MEFs treated with CytD (red region). For each gene, the image-based siRNA screen results are indicated as a color code representing the mean Z-score of the YAP nuclear:cytosolic ratio in WT (left side of the node) and Cav1KO cells (right side of the node). n=3.

# Figure S6

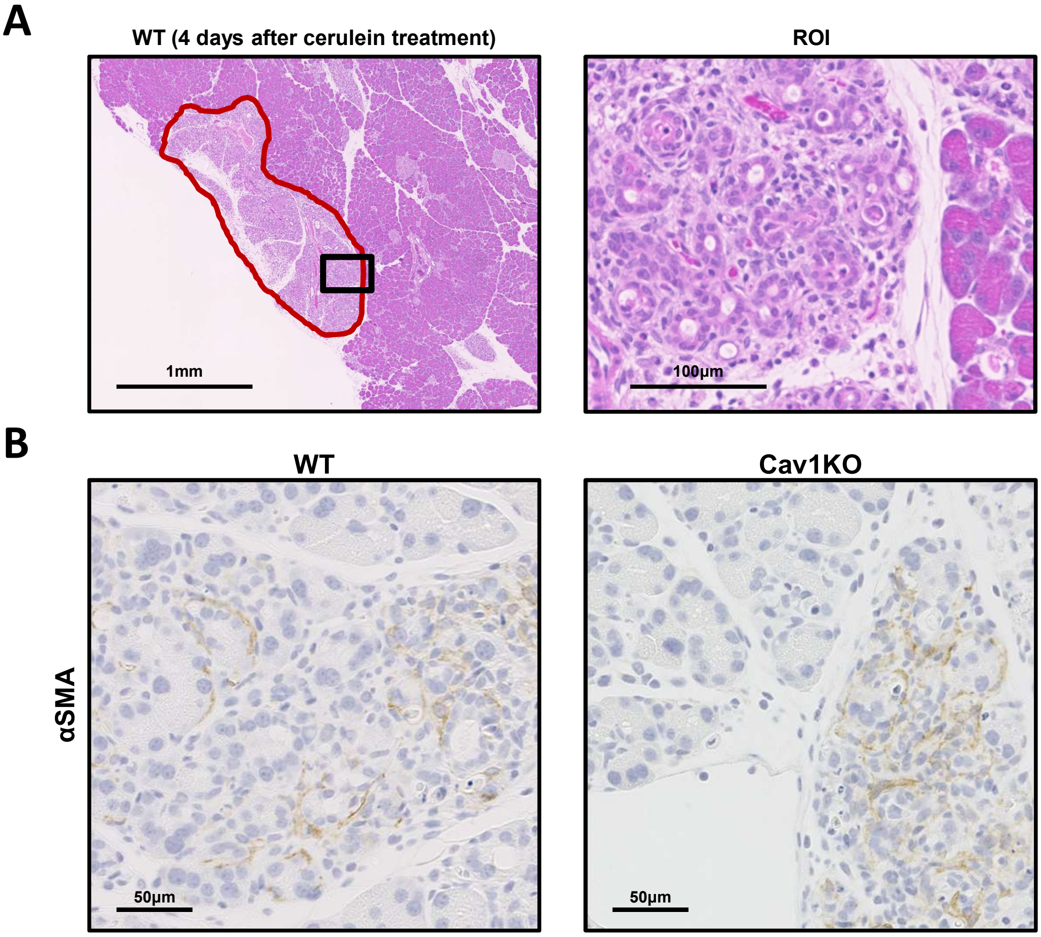


**Figure S6. Mouse pancreatic regions presenting extensive ADM and fibrosis after caerulein treatment. (Related to Figure 6) (A)** H&E staining. Damaged regions observed 4 days after caerulein administration in WT mice. The red line encloses a region presenting extensive acinar-to-ductal metaplasia (ADM). The right panel shows a zoomed view of the boxed ROI in the left image. **(B)** Immunohistochemistry analysis of αSMA expression in pancreatic tissue of WT and Cav1KO mice 4 days after caerulein treatment.

**Table S3. List of siRNAs used. (Related to STAR Methods)**

| ON-Target Plus Smart-pool siRNAs | |
|---|---|
| siRNA | IDENTIFIER |
| ON-Target Plus Smart-pool siRNA targeting YAP | Cat#L-046247-01 |
| ON-Target Plus Smart-pool siRNA targeting TAZ | Cat#L-041057-01 |
| ON-Target Plus Smart-pool siRNA targeting LATS1 | Cat#L-063467-01 |
| ON-Target Plus Smart-pool siRNA targeting LATS2 | Cat#L-044602-01 |
| ON-Target Plus Smart-pool siRNA targeting Nf2 | Cat#L-061566-00 |
| ON-Target Plus Smart-pool siRNA targeting Ywhah | Cat#L-043652-01 |
| ON-Target Plus Smart-pool siRNA targeting Nes | Cat#L-057300-01 |
| ON-Target Plus Smart-pool siRNA targeting Ywhae | Cat#L-062431-01 |
| ON-Target Plus Smart-pool siRNA targeting Baz1b | Cat#L-047645-01 |
| ON-Target Plus Smart-pool siRNA targeting Ywhag | Cat#L-059307-01 |
| ON-Target Plus Smart-pool siRNA targeting Col6a1 | Cat#L-043147-01 |
| ON-Target Plus Smart-pool siRNA targeting Noc2l | Cat#L-047536-02 |
| ON-Target Plus Smart-pool siRNA targeting Wdr75 | Cat#L-045131-01 |
| ON-Target Plus Smart-pool siRNA targeting Utp18 | Cat#L-064167-01 |
| ON-Target Plus Smart-pool siRNA targeting Klf2 | Cat#L-046974-01 |
| ON-Target Plus Smart-pool siRNA targeting Nol6 | Cat#L-052204-01 |
| ON-Target Plus Smart-pool siRNA targeting Gna13 | Cat#L-041077-00 |
| ON-Target Plus Smart-pool siRNA targeting Itih4 | Cat#L-046212-01 |
| ON-Target Plus Smart-pool siRNA targeting Slmap | Cat#L-059668-01 |
| ON-Target Plus Smart-pool siRNA targeting Magohb | Cat#L-047702-01 |
| ON-Target Plus Smart-pool siRNA targeting Nup155 | Cat#L-059426-01 |
| ON-Target Plus Smart-pool siRNA targeting C1qc | Cat#L-043163-01 |
| ON-Target Plus Smart-pool siRNA targeting Ranbp2 | Cat#L-042297-01 |
| ON-Target Plus Smart-pool siRNA targeting Bop1 | Cat#L-058593-01 |
| ON-Target Plus Smart-pool siRNA targeting Ahctf1 | Cat#L-051465-02 |
| ON-Target Plus Smart-pool siRNA targeting Fam160a2 | Cat#L-042848-01 |
| ON-Target Plus Smart-pool siRNA targeting Skp1a | Cat#L-060546-00 |
| ON-Target Plus Smart-pool siRNA targeting Ipo7 | Cat#L-058357-01 |
| ON-Target Plus Smart-pool siRNA targeting Rcc1 | Cat#L-051808-01 |
| ON-Target Plus Smart-pool siRNA targeting Nup98 | Cat#L-060137-01 |
| ON-Target Plus Smart-pool siRNA targeting Fgg | Cat#L-051775-01 |
| ON-Target Plus Smart-pool siRNA targeting Actr3 | Cat#L-046642-01 |
| ON-Target Plus Smart-pool siRNA targeting Ywhaq | Cat#L-048160-01 |
| ON-Target Plus Smart-pool siRNA targeting Arpc1b | Cat#L-047321-01 |
| ON-Target Plus Smart-pool siRNA targeting Actr2 | Cat#L-053600-01 |
| ON-Target Plus Smart-pool siRNA targeting Arpc4 | Cat#L-044870-01 |
| ON-Target Plus Smart-pool siRNA targeting Ywhah | Cat#L-043652-01 |
| ON-Target Plus Smart-pool siRNA targeting Hmgn1 | Cat#L-054969-00 |
| ON-Target Plus Smart-pool siRNA targeting Il1rapl1 | Cat#L-066757-01 |
| ON-Target Plus Smart-pool siRNA targeting Nup43 | Cat#L-052646-01 |
| ON-Target Plus Smart-pool siRNA targeting Trio | Cat#L-065649-00 |
| ON-Target Plus Smart-pool siRNA targeting Cd44 | Cat#L-041132-01 |
| ON-Target Plus Smart-pool siRNA targeting Col6a3 | Cat#L-065699-01 |
| ON-Target Plus Smart-pool siRNA targeting Runx1 | Cat#L-048982-00 |
| ON-Target Plus Smart-pool siRNA targeting Anxa4 | Cat#L-057375-00 |
| ON-Target Plus Smart-pool siRNA targeting Srgap3 | Cat#L-058941-01 |
| ON-Target Plus Smart-pool siRNA targeting Crlf1 | Cat#L-042918-01 |
| ON-Target Plus Smart-pool siRNA targeting Epn2 | Cat#L-043345-01 |
| ON-Target Plus Smart-pool siRNA targeting Mmp21 | Cat#L-054472-01 |
| ON-Target Plus Smart-pool siRNA targeting Ncoa3 | Cat#L-047722-02 |
| ON-Target Plus Smart-pool siRNA targeting Ywhab | Cat#L-059298-01 |
| ON-Target Plus Smart-pool siRNA targeting Inf2 | Cat#L-042811-01 |
| ON-Target Plus Smart-pool siRNA targeting Mrps5 | Cat#L-046026-01 |
| ON-Target Plus Smart-pool siRNA targeting Myl6b | Cat#L-054158-01 |
| ON-Target Plus Smart-pool siRNA targeting Disp2 | Cat#L-059002-01 |
| ON-Target Plus Smart-pool siRNA targeting Arpc5 | Cat#L-041235-01 |
| ON-Target Plus Smart-pool siRNA targeting Cttn | Cat#L-044721-00 |
| ON-Target Plus Smart-pool siRNA targeting Hmgb2 | Cat#L-066145-01 |
| ON-Target Plus Smart-pool siRNA targeting Smarce1 | Cat#L-051327-01 |
| ON-Target Plus Smart-pool siRNA targeting Aimp1 | Cat#L-045099-01 |
| ON-Target Plus Smart-pool siRNA targeting Arpc2 | Cat#L-043464-01 |

| ON-Target Plus Smart-pool siRNAs | |
|---|---|
| siRNA | IDENTIFIER |
| ON-Target Plus Smart-pool siRNA targeting Bzw1 | Cat#L-058621-01 |
| ON-Target Plus Smart-pool siRNA targeting Cyc1 | Cat#L-047759-01 |
| ON-Target Plus Smart-pool siRNA targeting Med14 | Cat#L-057957-01 |
| ON-Target Plus Smart-pool siRNA targeting Gnpat | Cat#L-040695-01 |
| ON-Target Plus Smart-pool siRNA targeting Kcnb2 | Cat#L-043145-00 |
| ON-Target Plus Smart-pool siRNA targeting Zbtb18 | Cat#L-043562-01 |
| ON-Target Plus Smart-pool siRNA targeting Dock7 | Cat#L-065434-01 |
| ON-Target Plus Smart-pool siRNA targeting Amot | Cat#L-058986-02 |
| ON-Target Plus Smart-pool siRNA targeting Arpc3 | Cat#L-040765-01 |
| ON-Target Plus Smart-pool siRNA targeting Myh7 | Cat#L-048355-01 |
| ON-Target Plus Smart-pool siRNA targeting Sp100 | Cat#L-043934-01 |
| ON-Target Plus Smart-pool siRNA targeting Pkp4 | Cat#L-045923-01 |
| ON-Target Plus Smart-pool siRNA targeting Apoa1 | Cat#L-042942-01 |
| ON-Target Plus Smart-pool siRNA targeting Nde1 | Cat#L-050911-01 |
| ON-Target Plus Smart-pool siRNA targeting Kcnma1 | Cat#L-041156-00 |
| ON-Target Plus Smart-pool siRNA targeting Sod2 | Cat#L-062893-00 |
| ON-Target Plus Smart-pool siRNA targeting Adam22 | Cat#L-054425-01 |
| ON-Target Plus Smart-pool siRNA targeting Grid2ip | Cat#L-053712-01 |
| ON-Target Plus Smart-pool siRNA targeting Myl2 | Cat#L-045013-01 |
| ON-Target Plus Smart-pool siRNA targeting Olig3 | Cat#L-048661-01 |
| ON-Target Plus Smart-pool siRNA targeting Plekhh3 | Cat#L-062851-01 |
| ON-Target Plus Smart-pool siRNA targeting Spink5 | Cat#L-055789-01 |
| ON-Target Plus Smart-pool siRNA targeting Antxr1 | Cat#L-048413-01 |
| ON-Target Plus Smart-pool siRNA targeting Micalcl | Cat#L-172668-00 |
| ON-Target Plus Smart-pool siRNA targeting Serpina9 | Cat#L-041728-01 |
| ON-Target Plus Smart-pool siRNA targeting Tbx5 | Cat#L-044182-01 |
| ON-Target Plus Smart-pool siRNA targeting Cys1 | Cat#L-053758-01 |
| ON-Target Plus Smart-pool siRNA targeting Rap1gap2 | Cat#L-040366-01 |
| ON-Target Plus Smart-pool siRNA targeting Usp40 | Cat#L-043598-01 |
| ON-Target Plus Smart-pool siRNA targeting Slc45a2 | Cat#L-061072-01 |
| ON-Target Plus Smart-pool siRNA targeting Capn5 | Cat#L-042053-01 |
| ON-Target Plus Smart-pool siRNA targeting Etv5 | Cat#L-062952-01 |
| ON-Target Plus Smart-pool siRNA targeting Map3k1 | Cat#L-041090-00 |
| ON-Target Plus Smart-pool siRNA targeting Incenp | Cat#L-049298-01 |
| ON-Target Plus Smart-pool siRNA Control | Cat#D-001810-01 |
| ON-Target Plus Smart-pool siRNA Control | Cat#D-001810-02 |

**Table S4. List of qRT-PCR primers used. (Related to STAR Methods)**

| Human Primers | | |
|---|---|---|
| Gene | | Sequence (5' -> 3') |
| *HPRT1* | Forward Primer | CCTGGCGTCGTGATTAGTGAT |
| | Reverse Primer | AGACGTTCAGTCCTGTCCATAA |
| *Beta-actin* | Forward Primer | CACCTTCCAGCAGATGTCGA |
| | Reverse Primer | AGCATTTGCGGTGGACGATGG |
| *CTGF* | Forward Primer | ACCGACTGGAAGACACGTTTG |
| | Reverse Primer | CCAGGTCAGCTTCGCAAGG |
| *ANKRD1* | Forward Primer | AGTAGAGGAACTGGTCACTGG |
| | Reverse Primer | TGTTTCTCGCTTTTCCACTGTT |
| Mouse primers | | |
| Gene | | Sequence (5' -> 3') |
| *Hprt1* | Forward Primer | GCTGGTGAAAAGGACCTCT |
| | Reverse Primer | CACAGGACTAGAACACCTGC |
| *Beta-actin* | Forward Primer | AGATGACCCAGATCATGTTTGA |
| | Reverse Primer | CACAGCCTGGATGGCTACGT |
| *Ctgf* | Forward Primer | GGACACCTAAAATCGCCAAGC |
| | Reverse Primer | ACTTAGCCCTGTATGTCTTCACA |
| *Ankrd1* | Forward Primer | GCTGGTAACAGGCAAAAAGAAC |
| | Reverse Primer | CCTCTCGCAGTTTCTCGCT |
| *Cyr61* | Forward Primer | CTGCGCTAAACAACTCAACGA |
| | Reverse Primer | GCAGATCCCTTTCAGAGCGG |
| *Cav1* | Forward Primer | CCGCGACCCCAAGCA |
| | Reverse Primer | CTGCAATCACATCTTCAAAGTC |
